# A systematic review and meta-analysis of the association between cardiovascular health determined by life's essential 8 and risk of mortality and major non-communicable diseases

**DOI:** 10.3389/fcvm.2025.1612056

**Published:** 2025-10-23

**Authors:** Guangkai Li, Yanfang Zhang, Qingxu Wu, Beibei Shi, Dexu Chen

**Affiliations:** ^1^ School of Physical Education, Shandong University, Jinan, China; ^2^ Department of Rheumatology, The Affiliated Hospital of Jining Medical University, Jining, China

**Keywords:** CVH, life's essential 8, mortality, cardiovascular disease, cancer, diabetes, dementia

## Abstract

**Objectives:**

This study aimed to explore the associations between cardiovascular health (CVH) and the risk of mortality and major non-communicable diseases by conducting a meta-analysis.

**Methods:**

Several databases including Pubmed, Embase, Web of science, Scopus were searched for studies exploring the prospective associations between ideal CVH and health outcomes compared with the poor CVH status and published up to January 20, 2025. Adjusted relative risks (RRs) were used to calculate pooled effect size using random-effect models.

**Results:**

This study included a total of 46 eligible studies. When comparing the ideal CVH score category to the poor CVH score category, the pooled RRs were 0.44 (95% CI: 0.40–0.48) for all-cause mortality, 0.33 (95% CI: 0.29–0.39) for CVD mortality, 0.51 (95% CI: 0.46–0.57) for total cancer mortality, 0.36 (95% CI: 0.33–0.39) for CVD, 0.75 (95% CI: 0.69–0.81) for total cancer and 0.65(95% CI: 0.55–0.96) for all-cause dementia, respectively. We also observed significant reduction of risk of diabetes, NAFLD, depression, anxiety, chronic kidney diseases, etc. Due to limited literatures and high heterogeneity, some of these results required further validation. Dose-response meta-analysis showed a linear reduction in the risk of all-cause mortality, total cancer mortality and a nonlinear reduction of CVD mortality and incident stroke, myocardial infarction.

**Conclusions:**

This study finds that ideal CVH score is strongly inversely associated with the risk of all-cause, CVD and total cancer mortality, as well as incident several common NCDs. There's a linear dose-response reduction of risk of all-cause mortality, total cancer mortality and a nonlinear dose-response reduction of risk of CVD mortality, incident CVD, stroke, myocardial infarction with the increase of CVH score.

**Systematic Review Registration:**

PROSPERO, identifier CRD42024494354.

## Introduction

Non-communicable diseases (NCDs) are the leading causes of global death. It was estimated that 40.5 million (71% of all deaths) of the 56.9 million deaths globally were from NCDs (1). And among NCDs, cardiovascular disease (CVD), cancers, respiratory diseases and diabetes are the top four killers (2). Population-based prevention strategies are critical for mitigating the global prevalence of NCDs, notably CVD, cancer, diabetes and its associated burdens. Key modifiable risk factors including obesity, sedentary lifestyle, poor diet, smoking, hypertension, hyperglycemia, dyslipidemia are strongly linked to the development of CVD and its related mortality. Addressing these determinants through systematic interventions is essential for reducing disease incidence, improving management outcomes, and alleviating the socioeconomic costs tied to NCDs.

In 2010, the American Heart Association (AHA) proposed the conception of cardiovascular health (CVH) based on seven health behaviors and factors. The seven components[diet, physical activity, smoking, body mass index (BMI), fasting blood glucose, total cholesterol and blood pressure] were subsequently called Life's Simple 7(LS7) (3). Each metric was classified as poor, intermediate, or ideal according to the thresholds provided by AHA. During the past decade, studies suggested strong, stepwise, inverse associations between the number of ideal CVH metrics with incident CVD, cancers, dementia, diabetes and mortality (4–15). However, there are several limitations of LS7. For example, some features of CVH component(i.e., diet) do not cover the full scope and the current quantification of metrics is less sensitive to interindividual differences (16). To overcome these limitations, the AHA introduced Life's Essential 8(LE8). LE8 added sleep as new metric and updated four metrics (diet, smoking exposure, blood lipids and blood glucose). A major difference between LE8 and LS7 is the scoring system of the components. Whereas each component in the LS7 score system ranged from 0 to 2, the new LE8 scoring system for each component ranges from 0 to 100 points, allowing generation of a new composite CVH score. In addition, compared with LS7, LE8 is more sensitive to changes in individual or population CVH when behaviors change (16).

According to a previous meta-analysis, people with the greatest number of ideal CVH metrics have a 45% lower risk of all-cause mortality, a 75% decline in CVD mortality and 80% lower risk of incident CVD compared those with the least number of ideal CVH metrics (17). Similar results were identified by another meta-analysis (18). Further exploration of the dose-response relationships is essential to determine optimal CVH thresholds that can inform evidence-based public health recommendations. Both Guo et al (18) and Aneni et al (19) suggested a strong inverse linear dose-response relationship between the number of ideal CVH metrics and all-cause, CVD mortality. Even one unit increase in ideal CVH metrics can result in 11% decline of all-cause mortality, 19% lower risk of CVD mortality (18). In addition, previous meta-analysis also identified an inverse linear dose-response relationship between the number of ideal CVH metrics and incident type 2 diabetes (20). However, the association between LE8 and the risk of major NCDs or mortality remains underexplored. A systematic evaluation of the associations between CVH assessed by LE8 score with mortality and major NCDs will aid in the promotion of CVH for public health.

To address this gap, we performed a systematic review and meta-analysis synthesizing evidence from prospective cohort studies on the association between Life's Essential 8 (LE8) scores and risks of mortality and major NCDs in adults ≥18 years. Beyond comparative assessments of health benefits between optimal LE8 and poor CVH, we further conducted dose-response analysis to quantify gradient relationships between LE8 scores and these outcomes.

## Methods

This systematic review was performed following the PRISMA 2020 guidelines (21) and was registered a priority in the PROSPERO database (CRD42024494354).

### Search strategy

We performed a systematic literature across four major databases (Pubmed, Embase, Scopus, Web of Science) up to 20 January 2025, using three key search domains: (1) cardiovascular health concepts (“cardiovascular health metrics”, “ideal cardiovascular health”, “ CVH” OR “life's essential 8”, “LE8”); (2) health outcomes (“mortality”, “all-cause mortality”, “death”, “cardiovascular disease*”, “stroke”, “cerebrovascular disease*”, “coronary heart disease*”, “cancer*”, “dementia”, “chronic kidney disease”, “frailty”, “depression”, “diabetes”, “non-alcoholic fatty liver disease”); (3) study design(“prospective”, “cohort”, “longitudinal”, “follow-up”). Boolean operators were strategically employed to combine search terms across these domains.

### Study selection

We implemented a dual-blind screening protocol to ensure methodological rigor. Two investigators (G.L. and Q.W.) independently conducted title/abstract screening in EndNote after duplicates removal, followed by full-text evaluation and manual inspection of reference lists in relevant reviews. Inter-rater discrepancies were adjudicated through consensus meetings with the research team.

The systematic review focused on examining relationships between cardiovascular health (CVH) as quantified by Life's Essential 8 (LE8) and clinical outcomes in adults ≥18 years without baseline severe comorbidities. Eligibility criteria required studies to: (1) employ prospective observational designs; (2) maintain ≥2-year follow-up duration; (3) report quantitative associations between CVH and ≥1 predefined endpoint, including all-cause/cardiovascular/cancer mortality, or incident non-communicable diseases (NCDs). Cardiovascular endpoints specifically encompassed myocardial infarction, atrial fibrillation, heart failure, stroke, and peripheral arterial disease.

### Data extraction

Two authors (G.L and Q.W) independently extracted information using a pre-designed spreadsheet, including first author, study location, publication year, cohort name, sex, age of participant, sample size, years of follow-up, person-years, number of deaths, cause of death, number of incident outcomes, assessment details for outcomes, and effect estimates, 95% confidence intervals(CIs) of mortality or incidence of non-communicable diseases. When methodological details regarding outcome ascertainment or exposure measurement were unavailable in selected articles, we cross-referenced supplementary publications from the same cohort studies to retrieve missing parameters. To improve the analytical consistency, the maximally adjusted effect size estimates (incorporating all available covariates) were extracted in the main analyses.

### Quality assessment

The Newcastle-Ottawa Scale (NOS) for Quality Assessment of Prospective Cohort Studies was used. Two investigators (G.L. and Q.W.) conducted parallel quality assessments, with discordant ratings resolved through structured consensus-building sessions. The refined NOS criteria emphasized: (1) cohort selection rigor, (2) exposure-outcome ascertainment validity, and (3) analytical completeness. Each criterion's fulfillment contributed to a granular quality stratification system, enabling precise differentiation between studies with optimal vs. suboptimal methodological characteristics.

### Data synthesis and analysis

We performed quantitative synthesis following prespecified meta-analysis protocols. Pooled relative risks (RRs) with 95% confidence intervals (CIs) were derived as primary effect size measures, incorporating hazard ratios (HRs) as RR equivalents per epidemiological convention. For studies reporting odds ratios (ORs), we implemented validated conversion algorithms (RR = OR/[(1 - P₀) + (P₀ × OR)]; P₀ = baseline outcome incidence in unexposed groups) to ensure metric comparability, referencing established methodology (22). We assessed the sensitive analysis by performing a leave-one-out analysis. The DerSimonian-Laird random-effects model was systematically applied to synthesize comparisons between optimal (highest CVH score category) and suboptimal (lowest CVH score category) cardiovascular health status. Meta-analytic thresholds required ≥2 methodologically comparable studies per clinical endpoint. Meta-regression was conducted to explore the source of heterogeneity. Subgroup analyses were performed according to age, sample size, region, duration of follow-up, CVH classification, economic status, exclusion of existence of major diseases related to mortality.

A dose-response meta-analysis was also conducted to examine the influence of ideal CVH on health outcomes using the method described elsewhere (23). This method allows estimating linear trends

Publication bias was assessed by the funnel plots and Egger's test. Stata 17.0 software was used to finish all these analyses. Statistical significance was set at *P* < 0.05.

## Results

**Figure 1:**
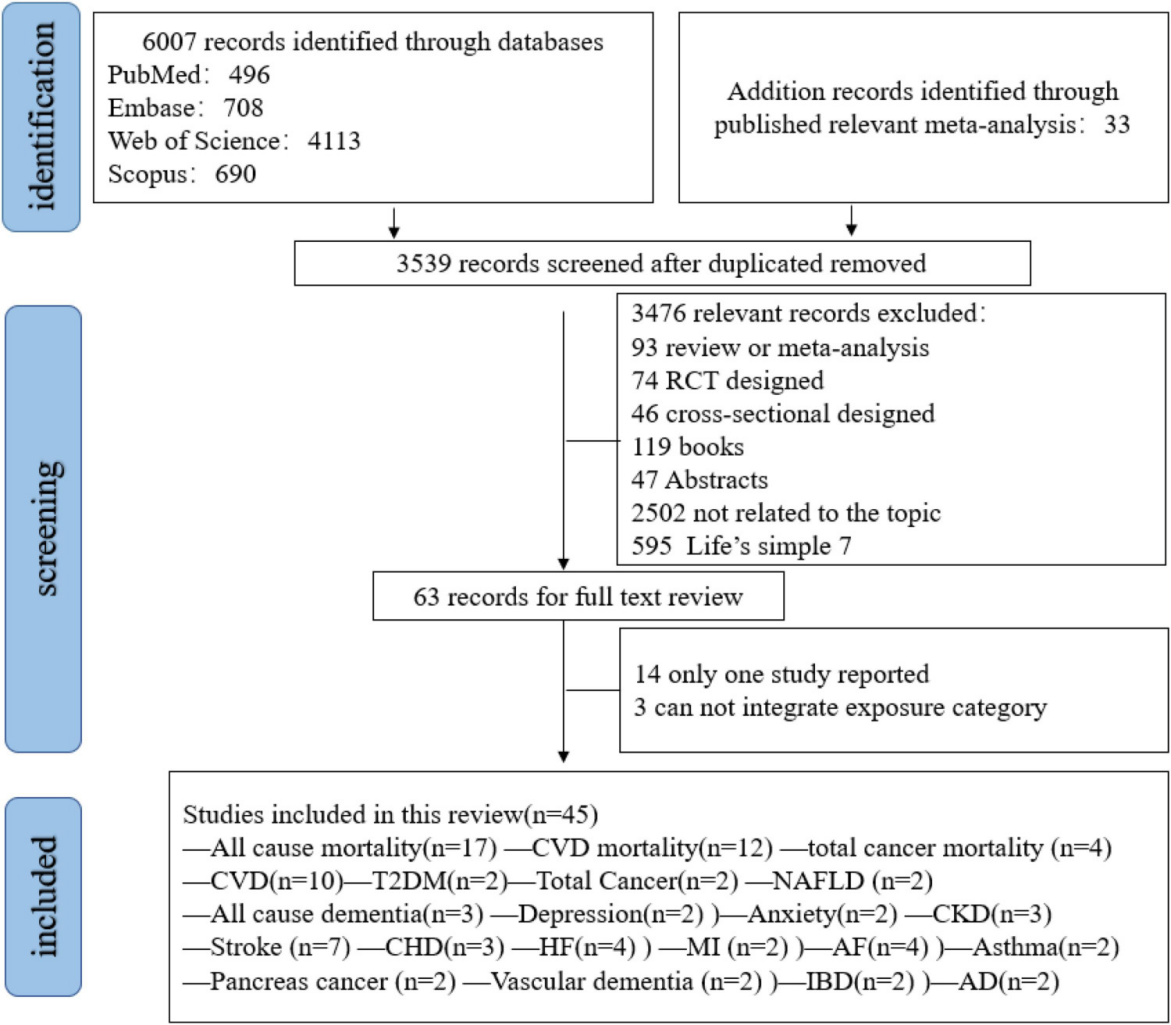
Flowchart of study selection.

Figure 1 shows the systematic search and study selection process. A total of 6,007 records were identified (Supplementary Table S1). And another 33 studies were retrieved by other sources. After removing 2,468 duplicates and an additional 3,572 records were screened through title and abstracts. Finally, 45 studies met the inclusion criteria. Among them, 22 outcomes (all-cause mortality, CVD mortality, total cancer mortality, CVD, total cancer, stroke, heart failure, myocardial infarction, coronary heart disease, atrial fibrillation, venous thromboembolism, pancreas cancer, diabetes, NAFLD, all-cause dementia, Alzheimer's disease, vascular dementia, CKD, asthma, depression and anxiety, inflammatory bowel disease) were eligible for meta-analysis synthesis (Figure 1). Some outcomes were excluded for analysis due to limited literatures (Supplementary Table S3).

### Study characteristics

The summarized characteristics of included studies can be found in Supplementary Table S4. These studies were published from 2023 to 2025 year (24–69). Sample size varied from 1,662 to 316,669. Among these studies, most were conducted in China and UK, while other studies were performed in the USA, Finland, Spain (Supplementary Table S3). The median follow-up duration ranged from 2.3 to 33 years (62). Most studies included both men and women, while two study included men only (51, 64). Most studies used CVH score ranging from 0 to 100 for each metric and the overall CVH score was calculated as the unweighted average of all 8 components scores, except two studies which did not calculated the average of all 8 component scores (51, 64). In addition, most studies used a 3 level category of CVH score using 0–49 as low, 50–79 as moderate and 80–100 as high level while 5 studies used four category level (33, 39, 48, 51, 64) and one study used five categories (50) by quartile and quintile, respectively.

### Risk of bias

The risk of bias was systematically evaluated through the Newcastle–Ottawa scale (NOS) framework with full assessment metrics summarized in Supplementary Table S2. Most studies were assigned over 7 stars, with only one study were assigned 5 stars (Supplementary Table S2).

### All-cause mortality

**Figure 2:**
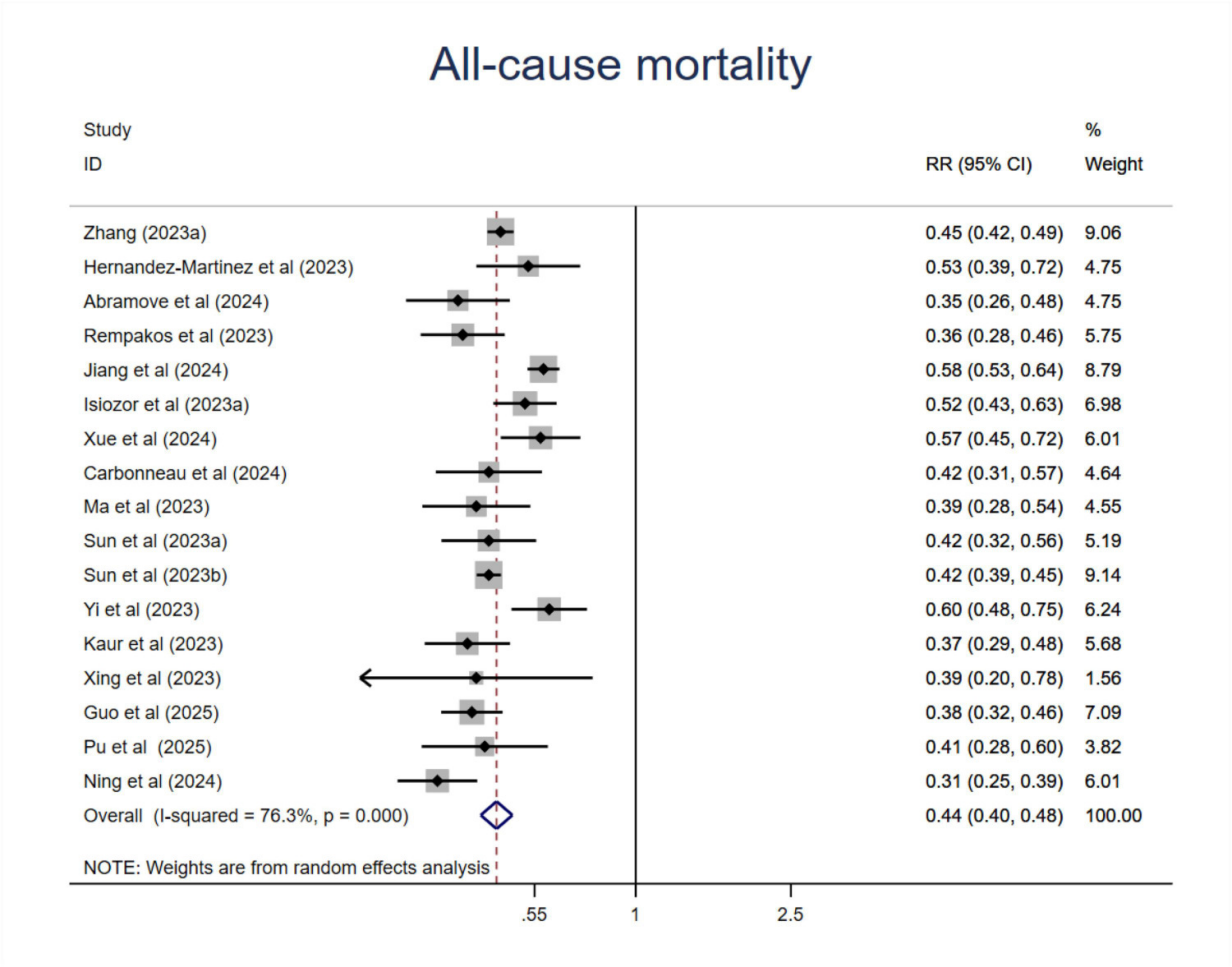
The associations between ideal CVH and all-cause mortality.

As shown in Figure 2, compared with those at the lowest level of CVH, participants at the highest CVH category had a 56% lower risk of all-cause mortality (RR = 0.44; 95%CI 0.40–0.48; P < 0.001) (Figure 2). Although the heterogeneity was high(I^2^ = 76.3%, P < 0.001), the association was all in the same direction, with an RR < 1 in all studies. Sensitive analysis by the exclusion of any other individual study did not substantially change this result (Supplementary Table S5). We then conducted meta-regression analysis of potential moderators including mean age, sample size, publication year, region where the study was conducted(country), length of follow-up, sex ratio (female proportion), events which did not find the source of heterogeneity (Supplementary Table S6, Figures S1–S7). Then we performed subgroup analysis of moderators like mean age, sample size, region where the study was conducted (continent), length of follow-up, CVH classification (3 or 4 levels), economic status, exclusions of major diseases related to mortality. To directly compare the difference between subgroups, we used Review Manger software, and we did not observe any significant differences among subgroups in mean age, sample size, length of follow-up. However, we found that studies conducted in Asia or developing countries showed less reduction of risk compared with studies conducted in Europe and North America or developed countries. In addition, we found that compared with those studies used traditional 3 level CVH categories, those used 4 level (quartiles) CVH categories had less reduction of risk (Supplementary Figures S8–S13).

**Figure 3:**
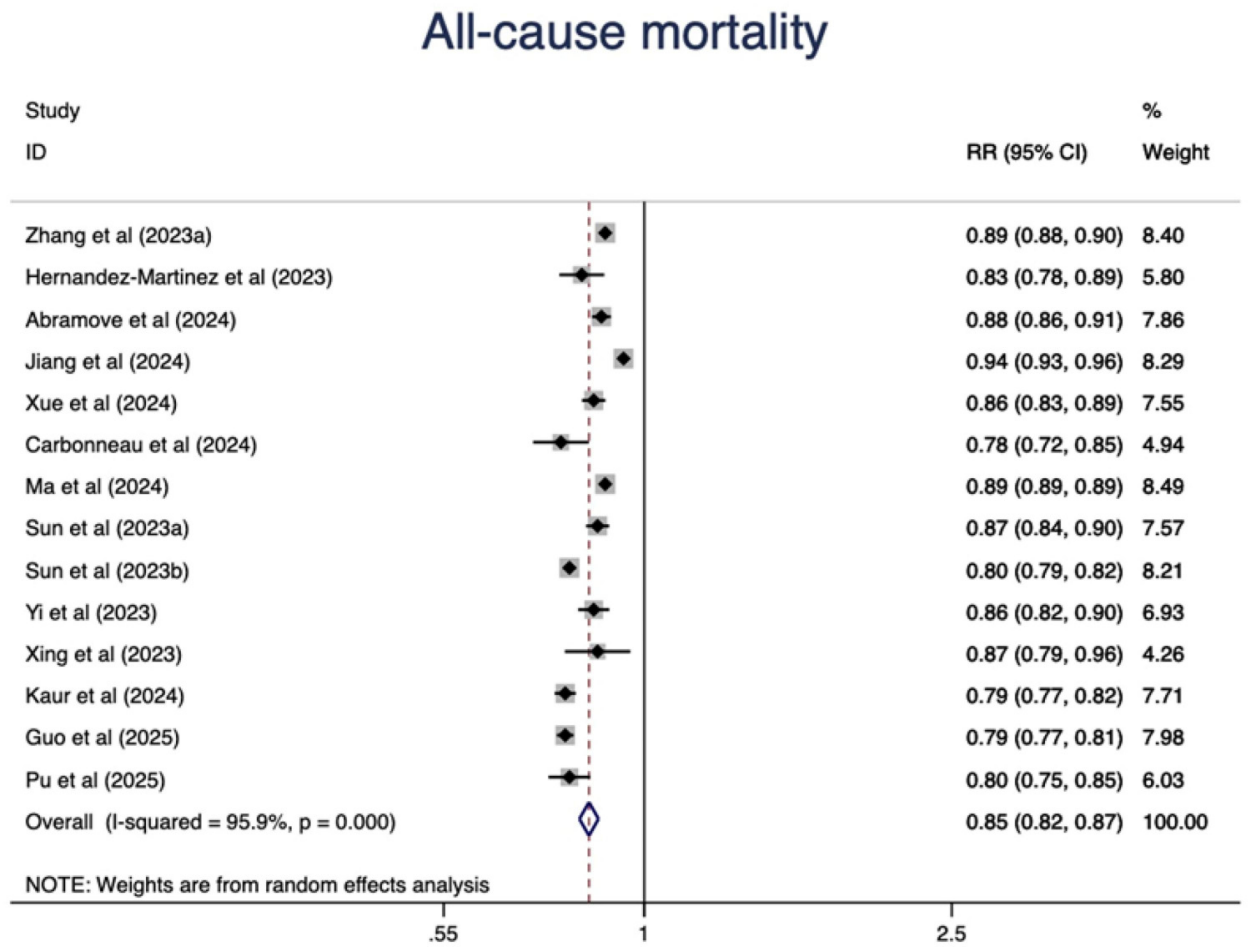
The association between CVH (per 10 points increase) and all-cause mortality.

**Figure 4:**
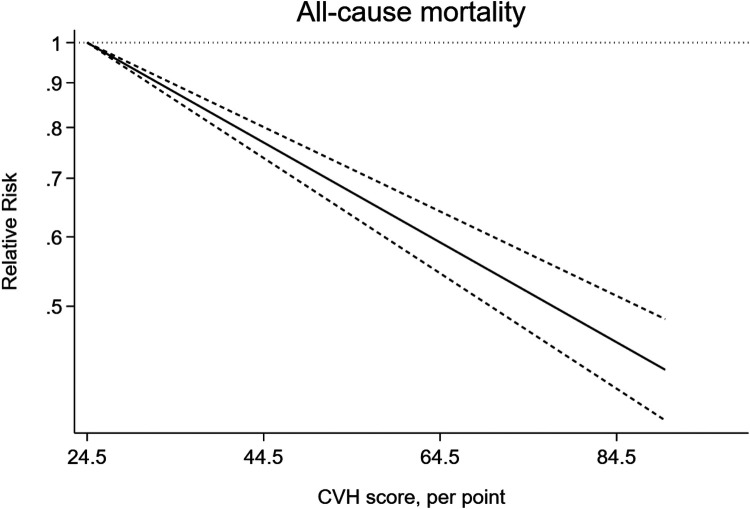
Linear dose-response meta-analysis of the association between CVH and all-cause mortality.

In addition, we observed a 15% lower risk of all-cause mortality per 10 points increase of CVH score (RR = 0.85, 95%CI 0.82–0.87) (Figure 3). The test for nonlinearity (P for nonlinearity = 0.056) supported a linear association with 1% reduction per point increase (RR = 0.99) (Figure 4).

### CVD mortality

**Figure 5:**
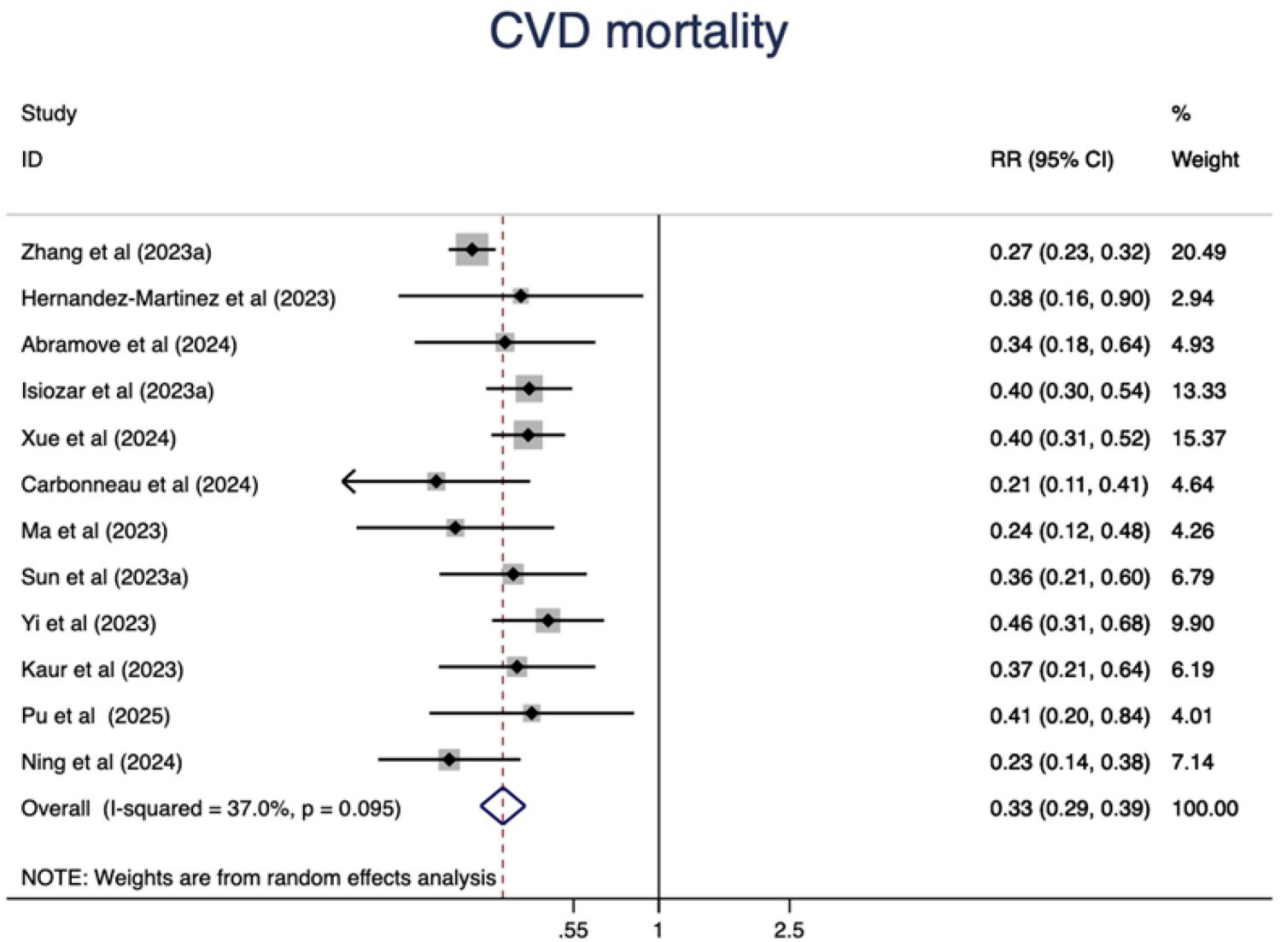
The associations between ideal CVH and CVD mortality.

Higher CVH status were associated with a 67% lower risk of CVD mortality(RR = 0.33, 95%CI 0.29–0.39, P < 0.001) (Figure 5). The heterogeneity was moderate (I^2^ = 37.0%, *P* = 0.095) and was not substantially changed by the leave-one-out analysis (Supplementary Table S5).

**Figure 6:**
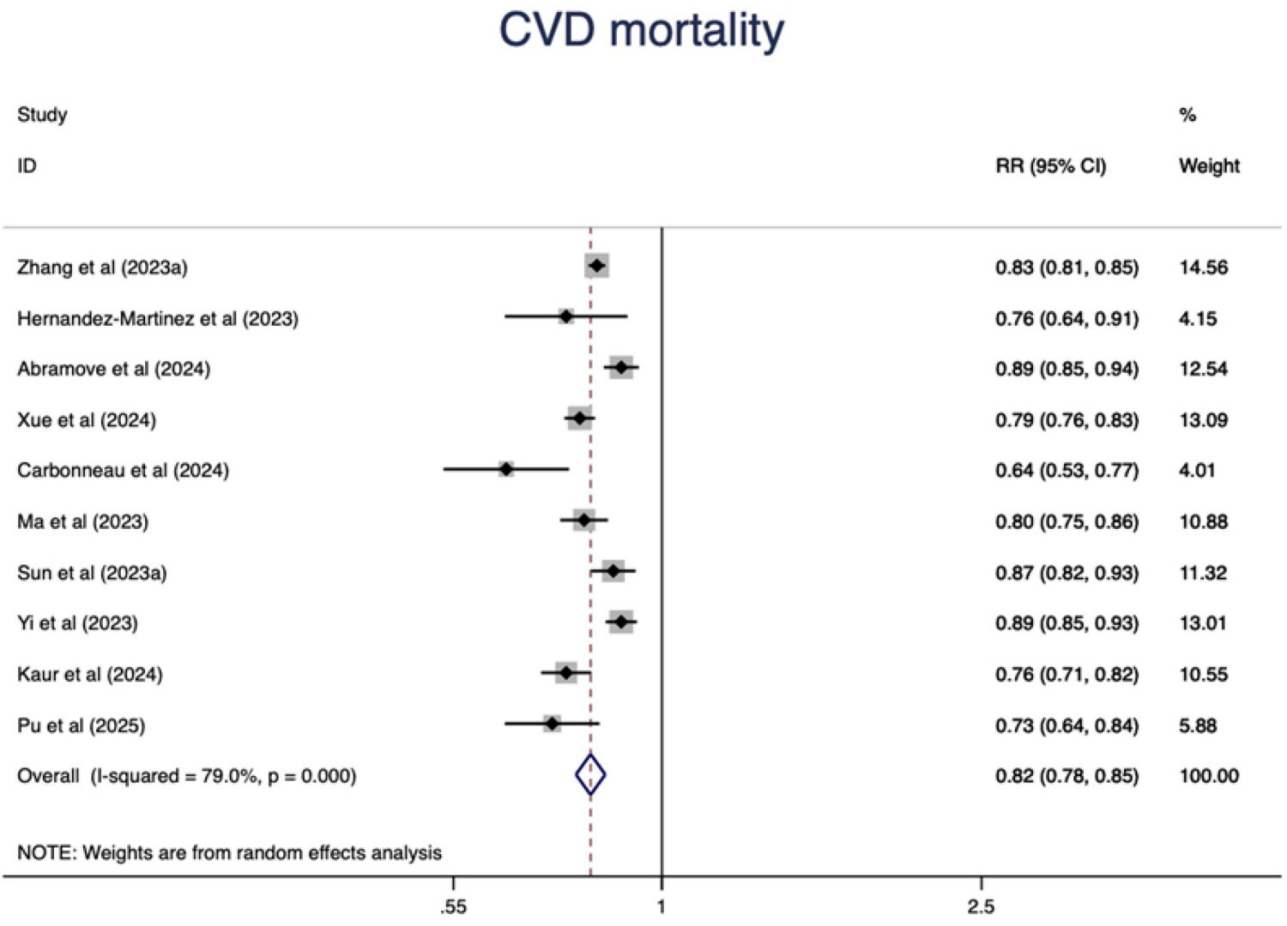
Linear dose-response meta-analysis of the associations between CVH (per 10 points increase) and CVD mortality.

**Figure 7:**
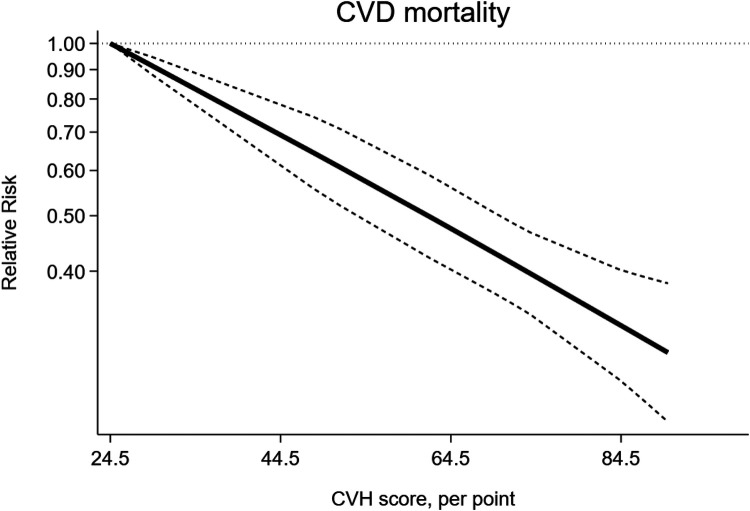
Non-linear dose-response meta-analysis of the association between CVH and CVD mortality.

The dose-response analysis of CVH per 10 points increase identified a 18% lower risk of CVD mortality (RR = 0.82, 95%CI 0.78–0.85) (Figure 6). The test for nonlinearity (P for nonlinearity = 0.03) supported a non-linear association (Figure 7).

### Total cancer mortality

**Figure 8:**
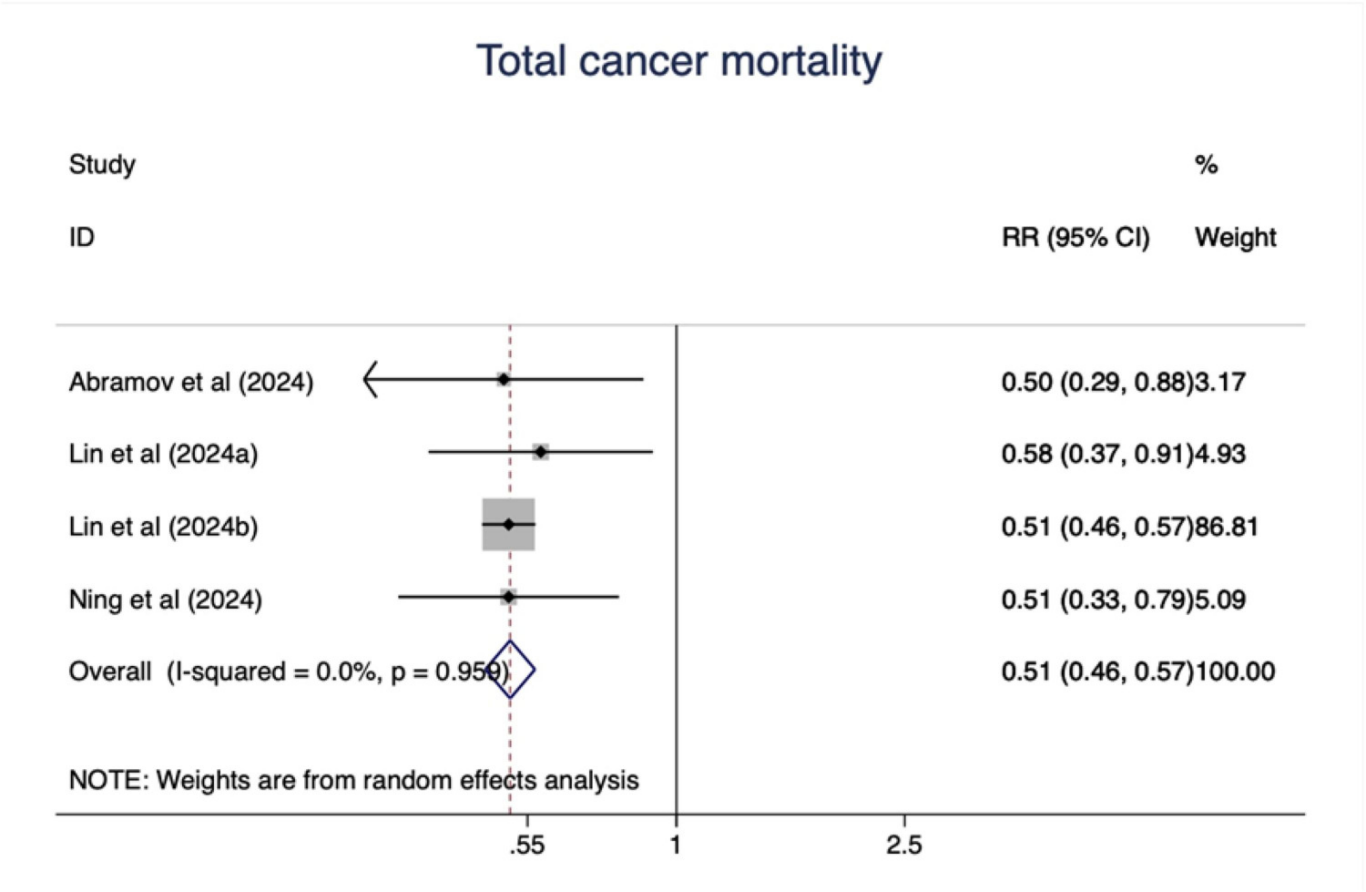
The associations between low and ideal CVH and total cancer mortality.

**Figure 9:**
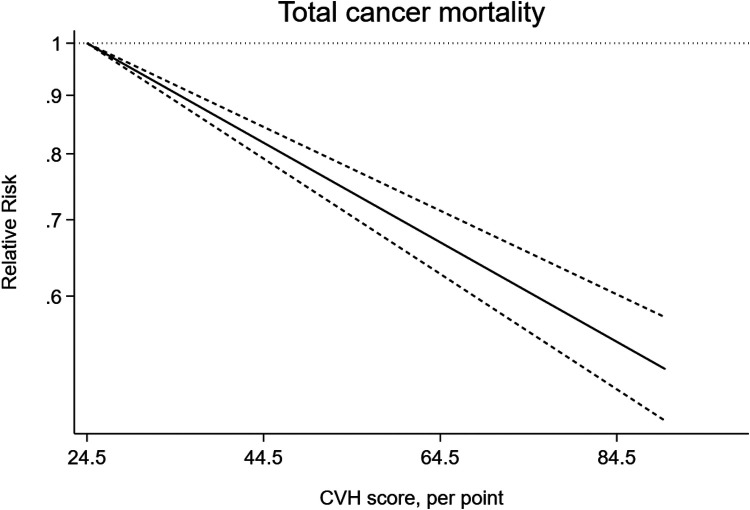
Linear dose-response meta-analysis of the association between CVH and total cancer mortality.

As shown in Figure 8, ideal CVH status was associated with significant decline of total cancer mortality (RR = 0.51; 95%CI 0.46–0.57) (Figure 8). The test for nonlinearity (P for nonlinearity = 0.65) supported a linear association with 1% reduction per points increase (R = 0.99) (Figure 9).

### CVD incidence

**Figure 10:**
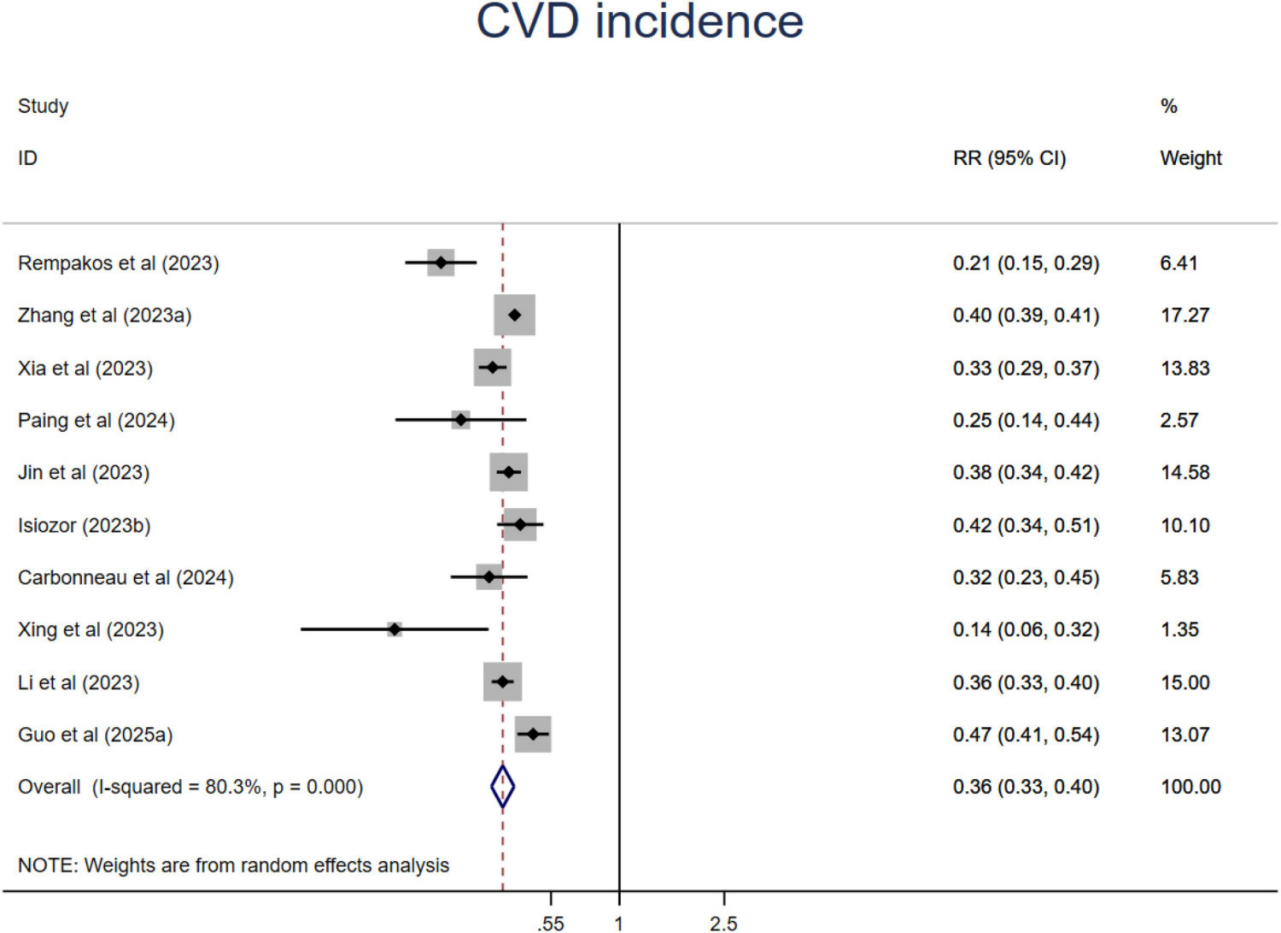
The associations between low and ideal CVH and CVD.

A synthesis of ten studies with 11 comparisons suggested that people at the ideal CVH category had a 64% significant lower risk of CVD (RR = 0.36, 95%CI 0.33–0.40, P < 0.001) than those at the least CVH category (Figure 10). The heterogeneity was high (I^2^ = 80.3%, P < 0.001). The leave-one-out analysis did not identify substantial change (Supplementary Table S5). Meta-regression analysis was conducted to examine potential moderators including mean age, sample size, events, publication year, region where the study was conducted (country), length of follow-up, sex ratio (female proportion) which suggested that mean age might be the source of high heterogeneity (Supplementary Table S6, Figures S14–S20). This finding was supported by the subsequent subgroup analysis that relative younger aged subgroup showed much lower risk of all-cause mortality. In addition, studies from North America and studies with small sample size also showed significant lower risk of CVD incidence. There are no significant differences among subgroups in length of follow-up, CVH classification, economic status (Supplementary Figures S21–S26).

**Figure 11:**
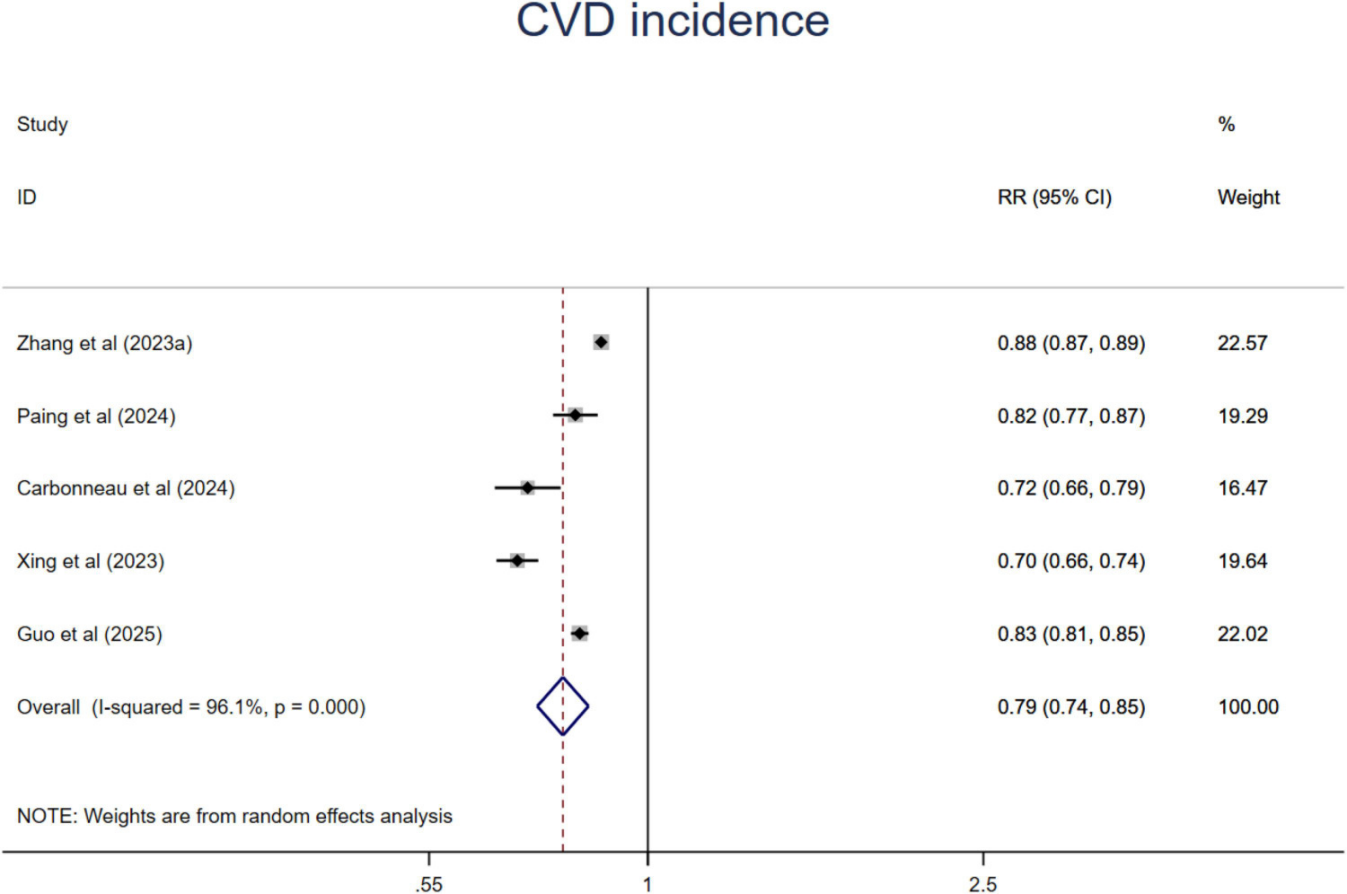
Linear dose-response meta-analysis of the associations between CVH (per 10 points increase) and CVD.

**Figure 12:**
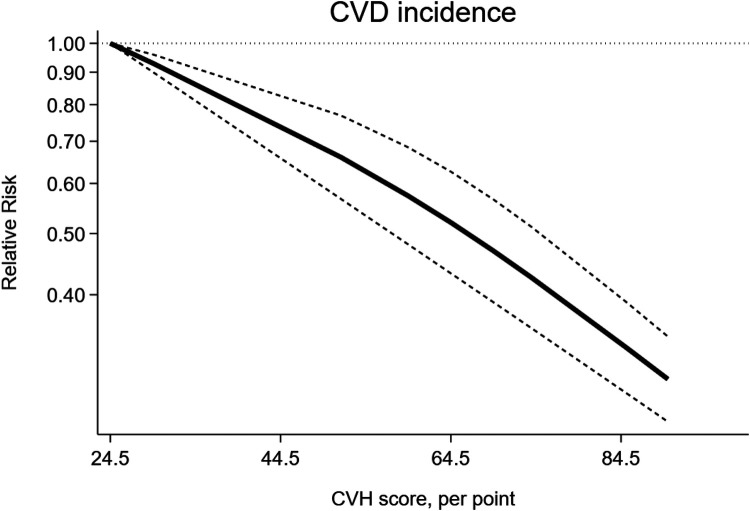
Non-linear dose-response meta-analysis of the association between CVH and CVD incidence.

The dose-response analysis of CVH per 10 points increase demonstrated a 21% lower risk of CVD mortality (RR = 0.79, 95%CI 0.74–0.85) (Figure 11). The test for nonlinearity (P for nonlinearity <0.001) supported a non-linear association (Figure 12).

### NAFLD incidence

**Figure 13:**
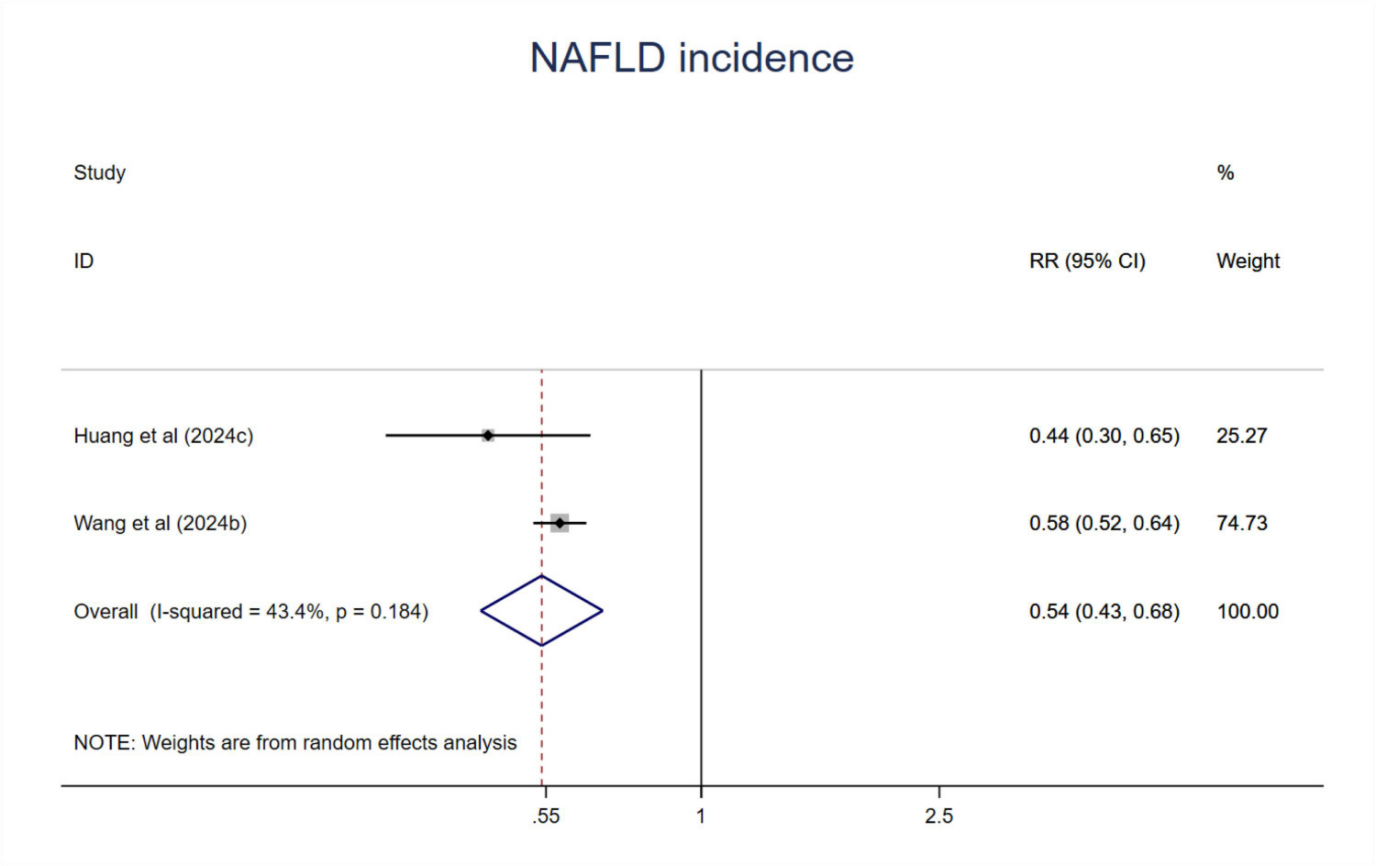
The associations between ideal CVH and NAFLD.

Two studies were included in the meta-analysis. Participants at the ideal CVH had a 46% lower risk of NAFLD (RR = 0.54; 95%CI 0.43–0.68; P < 0.001) (Figure 13) compared with poor or the least CVH groups. The heterogeneity was moderate (I^2^ = 43.4%, P > 0.1) (61).

### All-cause dementia incidence

**Figure 14:**
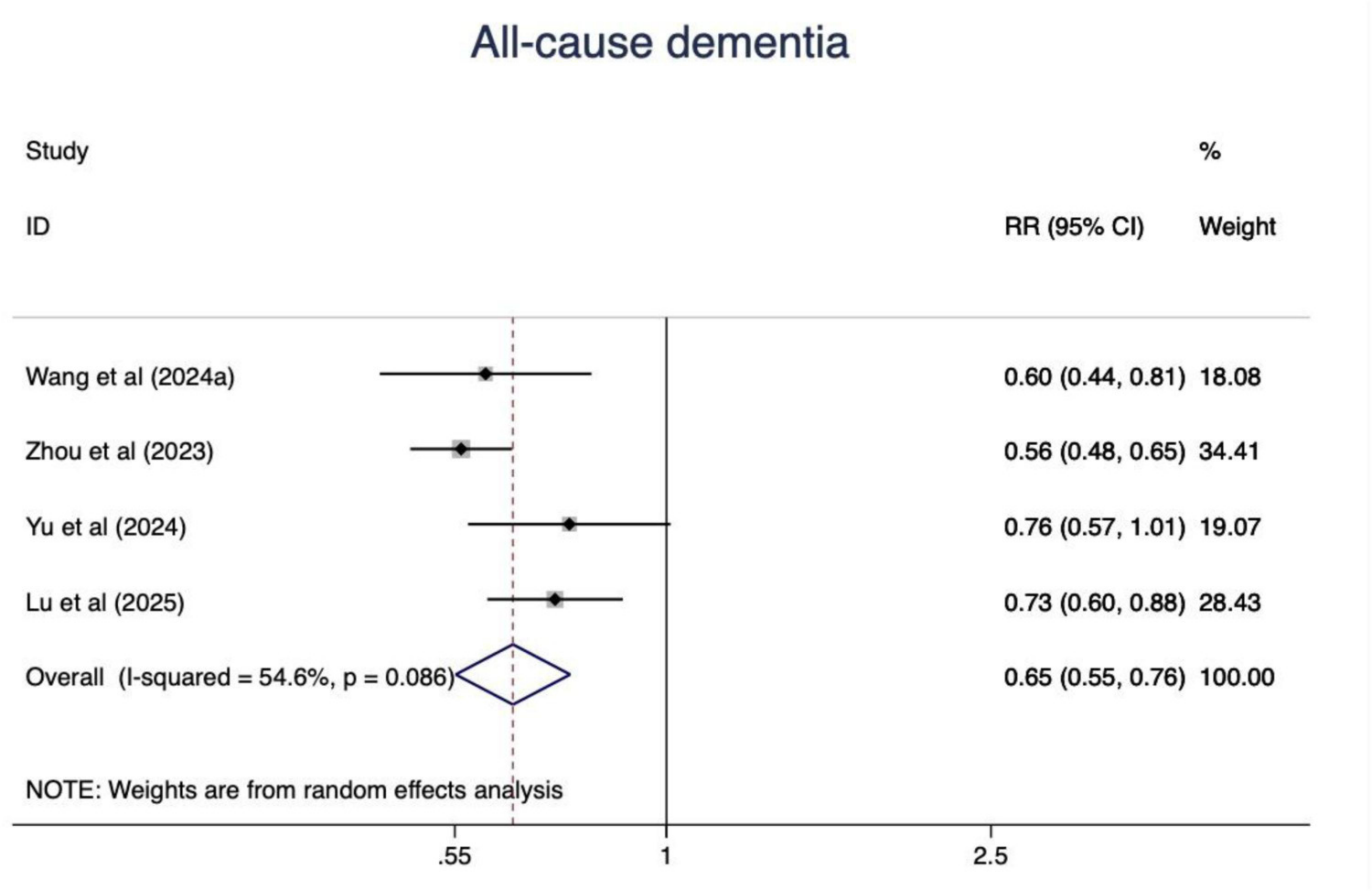
The associations between ideal CVH and all-cause dementia.

Four studies were synthesized and people with the ideal CVH status had a 35% lower risk of dementia (RR = 0.65, 95%CI 0.55–0.96, P < 0.001) (Figure 14). The heterogeneity was moderate(I^2^ = 54.6%, *P* = 0.086). The leave-one-out analysis did not show any substantial change.

### Other chronic conditions

In addition, people with ideal CVH status had a 25% lower risk of total cancer (RR = 0.75; 95%CI 0.69–0.81, P < 0.001), 88% lower risk of diabetes (RR = 0.12, 95%CI 0.02–0.61, *P* = 0.011), 70% lower risk of chronic kidney disease (RR = 0.30; 95%CI 0.10–0.87; *P* = 0.026), 56% lower risk of depression (RR = 0.44; 95%CI 0.38–0.52; P < 0.001), 40% lower risk of anxiety (RR = 0.60; 95%CI 0.46–0.77; P < 0.001), 54% lower risk of stroke (RR = 0.46; 95%CI 0.40–0.53; P < 0.001), 67% lower risk of coronary heart disease (RR = 0.33; 95%CI 0.30–0.36; P < 0.001), 64% lower risk of heart failure (RR = 0.36; 95%CI 0.29–0.45; P < 0.001), 80% lower risk of myocardial infarction (RR = 0.20; 95%CI 0.06–0.63; P < 0.001), 34% lower risk of atrial fibrillation (RR = 0.66; 95%CI 0.61–0.72; P < 0.001), 62% lower risk of hypertension(RR = 0.38; 95%CI 0.18–0.77; P < 0.001), 64% lower risk of pancreas cancer (RR = 0.36; 95%CI 0.23–0.57; P < 0.001), 69% lower risk of vascular dementia (RR = 0.31; 95%CI 0.23–0.41; P < 0.001), 48% lower risk of asthma (RR = 0.52; 95%CI 0.48–0.47; P < 0.001), 40% lower risk of inflammatory bowel disease (RR = 0.40; 95%CI 0.45–0.79; P < 0.001) (Supplementary Figures S27–S42), compared with poor or the least CVH groups. And there was no significant association between high CVH and Alzheimer's disease (RR = 0.84; 95%CI 0.68–1.05; P < 0.001) (Supplementary Figure S42). However, due to limited literatures in most above outcomes, the reliability of these results requires further validation with more prospective cohort studies in future, particularly for those with high heterogeneity.

Moreover, the test for nonlinearity supported a non-linear association between CVH and CKD (P for nonlinearity<0.001), stroke (P for nonlinearity <0.001), myocardial infarction (P for nonlinearity <0.001) (Supplementary Figures S43–S45).

### Publication bias

The publication bias was assessed by funnel plot and Egger's test if necessary. Egger's test suggested that there is no publication bias in all-cause mortality(*P* = 0.466), CVD mortality(*P* = 0.573), CVD incidence(*P* = 0.058) (Supplementary Figures S46–S51). For outcomes with less than ten studies, the funnel plot analysis can not rule out the possibility of publication bias and need more future studies to further analyze the publication bias (Supplementary Figures S52–S70).

## Discussion

This systematic review demonstrates significant cardiometabolic and other health benefits associated with optimal CVH as quantified by the LE8 metric. Our results indicate that participants in the highest CVH score category experienced a 56% lower risk of all-cause mortality, a 67% lower risk of CVD mortality, a 49% lower risk of total cancer mortality, a 64% lower risk of CVD, a 25% lower risk of total cancer incidence, 46% lower risk of NAFLD and 88% lower risk of type 2 diabetes and a 35% lower risk of all-cause dementia compared to those in the lowest CVH score category. Most of these findings were consistent across sensitivity analyses. However, the reliability of some results requires further validation with more prospective cohort studies in future, particularly for those with high heterogeneity. In addition, A clear linear association was observed between CVH scores and the risk of all-cause mortality, total cancer mortality. In contrast, a non-linear relationship was identified between CVH scores and the risk of CVD mortality, CVD, stroke, myocardial infarction, and CKD.

The association between CVH and the risk of all-cause mortality, CVD mortality, and incident CVD has been previously reviewed using old definition of CVH, namely the LS7 metrics (17–19, 70). For instance, Fang et al. demonstrated that achieving a highest number of ideal CVH metrics (5–7) was associated with a significantly lower risk of all-cause mortality (RR = 0.55), CVD mortality (RR = 0.25), and incident CVD (RR = 0.20) compared to achieving the lowest number of ideal CVH metrics(0–2) (17). Guo et al. reported similar findings, with overall RRs of 0.54 for all-cause mortality, 0.30 for CVD mortality, and 0.22 for incident CVD when comparing the greatest to the lowest categories of ideal CVH metrics (18). The newly proposed LE8 framework not only incorporates sleep health into the CVH construct based on strong evidence linking sleep duration and cardiometabolic health (71), but also introduces a novel scoring algorithm to assess CVH. The present study systematically examined the association between CVH scores rather than the number of ideal metrics achieved, and the risk of all-cause, CVD, and total cancer mortality. We found that participants in the highest CVH score category exhibited significantly lower risks of all-cause mortality (RR = 0.44), CVD mortality (RR = 0.33), and CVD (RR = 0.36). And we for the first time revealed that high CVH score is associated with lower risk of total cancer mortality (RR = 0.51). These findings highlighted the fact that participants with higher CVH scores experience a substantial reduction in mortality and CVD incidence risk. These results are consistent with those of a recently published systematic review, which also reported that higher CVH was associated with significantly lower risks of all-cause mortality, CVD mortality, and incident CVD (72).

However, the high heterogeneity undermined the reliability of the results of all-cause mortality and incident CVD analysis. It also compromised the generalizability and translational impact of this study. Meta-regression did not identify any source of heterogeneity for the all-cause mortality. The variability in covariate adjustments across included studies might bias the polled estimates. For instance, the study led by Rempakos (62) only adjusted age and sex while other important covariates were not considered. In addition, most of the eligible 17 studies adjusted educational level (*n* = 12), race or ethnicity (*n* = 11), economic-related covariates (*n* = 11) while only 7 adjusted drinking status, CVD or cancer history. Marital status was adjusted only in four studies while it has been proved to be related to all-cause and cause-specific mortality (73). Moreover, in addition to these environmental exposures, genetics are known to play important roles in shaping health and mortality. There are only one studies that adjusted the polygenic risk scores (65). These unadjusted covariates residual confounding could bias the estimates. In addition, the subgroup analysis stratified by key study characteristics demonstrated no significant differences among different age, sample size, length of follow-up subgroups. It's noteworthy that studies conducted in Asia and developing countries showed less reduction of all-cause mortality risk. Meta-regression suggested that age was the source of heterogeneity for the CVD outcome. And there are significant differences among age subgroups with younger aged subgroups having larger reduction of risk. It's possible that younger participants have better health status that having small number of events. Moreover, subgroup analysis suggested that studies conduced in North America have the largest reduction of CVD risk. Because studies in subgroup shares same studies in the younger age subgroups.

How high CVH contribute to lower risk of mortality and CVD should be deeply understood. Previous studies have suggested that adults with high (12–14 points) or even moderate (8–11 points) LS7 scores exhibit significantly lower odds of coronary artery calcium, reduced carotid intima-media thickness, and lower left ventricular mass compare to adults with low LS7 scores (74). Biologically mechanism investigations have identified several potential pathways involving inflammation, endothelial function, atherosclerosis, cardiac stress, and epigenetics (75, 76). In fact, all four health behaviors (smoking, diet, physical activity and sleep) and the four health factors (BMI, cholesterol, glucose and blood pressure) contribute to the risks of health outcomes and have been jointly or independently associated with cardiometabolic health (77–80). Furthermore, these factors are also recognized as common risk factors for the global disease burden. In 2021, high systolic blood pressure, smoking, high fasting plasma glucose, and high BMI contributed 7.8%, 5.7%, 5.4%, and 4.5% to the total disability-adjusted life-years (DALYs), respectively (81). Therefore, achieving a high score in CVH metrics would significantly reduce the risk of all-cause mortality, CVD mortality, and CVD events.

Additionally, our study is the first to identify a linear dose-response relationship between LE8 score and all-cause mortality. This finding aligns with the linear dose-response relationship between CVH metrics and all-cause mortality reported in previous reviews base on the number of ideal CVH metrics (18, 19). However, unlike those reviews which found a linear dose-response relationship between the number of ideal CVH metrics and outcomes (18, 19), we observed a non-linear dose-response relationship between LE8 score and CVD mortality. We also analyzed the association between CVH and individual CVD events, including stroke, myocardial infarction, coronary heart disease (CHD), heart failure(HF), and atrial fibrillation(AF). Similar to Sebastian's study (72), which found that a high LE8 score was associated with a 48% lower risk of stroke and a 56% lower risk of CHD, our analysis identified a 54% lower risk of stroke and a 67% lower risk of CHD, respectively. Our study also identified a 64% and 80% lower risk of HF and AF, respectively.

Moreover, several studies have explored the association between the LE8 metrics and risk of type 2 diabetes. A previous meta-analysis indicated that individuals with the highest number of ideal CVH metrics had a 64% lower risk of developing diabetes compared to those in the lowest category. Additionally, a nonlinear dose-response meta-analysis suggested a monotonic reduction in the risk of diabetes (20). Similarly, our finding revealed that participants in the highest LE8 score category exhibited a significant lower risk of diabetes. It's rational that high CVH has a positive effect on risk of diabetes. It is reasonable to infer that high CVH scores have a positive effect on reducing the risk of diabetes, given that CVD and diabetes share considerable common risk factors, such as physical inactivity, obesity and unhealthy diet. However, due to the limited availability of eligible literatures, the heterogeneity is very high and hard to explore source of heterogeneity.

In addition, no meta-analyses have assessed the relationship between the ideal CVH status and the risk of cancer. A previous prospective cohort study reported that after a median follow-up of 13 years, individuals with the lowest number of ideal CVH metrics had a 52% greater risk of incident cancer compared to those with highest number of CVH metrics (15). Our finding similarly indicated a modestly lower risk of incident cancer among individuals with high CVH scores. Several components of LE8 are also recognized as risk factors of cancer. For instance, globally in 2019, the leading risk factors contributing to cancer deaths were smoking, followed by alcohol use, high BMI, high fasting plasma glucose, and unhealthy diet (82). Additionally, physical inactivity is a common risk factor for various cancers, including colon and lung cancer (83). The relationship between physical activity, sedentary behavior, and obesity with cancer incidence can be explained by the interaction involving endogenous sex steroids and metabolic hormones, insulin sensitivity, and chronic inflammation (84). However, due to the limited availability of relevant literatures, a dose-response meta-analysis was not feasible in our study.

Similarly, no meta-analyses have evaluated the relationship between ideal CVH status and the risk of NAFLD. NAFLD is a major cause of liver disease worldwide, with its global prevalence increasing rapidly. The estimated global incidence of NAFLD is 4,613 cases per 100,000 person years, and overweight/obese individuals are approximately threefold more likely to develop NAFLD compared to those with normal weight. Additionally, smokers had higher NAFLD incidence than non-smokers (85). NAFLD is associated with an increased risk of CVD (86) and T2DM (87). Numerous studies have demonstrated that lifestyle modification, such as healthy diet, physical activity, and weight loss, are effective strategies for the prevention and management of NAFLD in clinical practice (88). Therefore, it is reasonable to hypothesize that ideal CVH status have a positive effect on reducing the risk of NAFLD. Our results indicated that individuals in the highest CVH score category had a substantially lower risk of new incident NAFLD compared to those in the lowest CVH score category. Although there are only two studies eligible for NAFLD analysis, they consistently reported protective effects of ideal CVH status. Previous studies have shown that NAFLD is significantly associated with metabolic syndrome and healthy lifestyles which are intrinsic components of LE8 (89, 90). Obesity and related inflammation promote insulin resistance which induces inappropriate lipolysis and lead to elevated free fatty acid in the circulation. These fatty acids were uptaken by the liver and hepatic de-novo lipogenesis together contribute to the NAFLD.

The relationship between CVH and dementia has been previously analyzed. Wu et al. (91) suggested that following the LS7, individuals with the highest number of ideal CVH metrics had a 6% lower risk of dementia, showing a linear association with late-life dementia risk. However, a J-shaped association was observed between late-life CVH scores and dementia risk. Our study found a 35% lower risk of all-cause dementia. We also analyzed vascular dementia and AD, and high CVH scores were consistently negatively associated with incidence of both vascular dementia and AD. It is reasonable to conclude that all four health behaviors (smoking, diet, physical activity, and sleep) and the four health factors (BMI, cholesterol, glucose, and blood pressure) contribute to the risks of dementia. Previous studies have provided strong evidence that physical inactivity, smoking, unhealthy diet, obesity, and high blood pressure are independently or jointly associated with dementia (92, 93).

One of the strengths of this study is the quantification of the dose-response association between the newly proposed LE8 and various health outcomes. However, our meta-analysis has several limitations. First, there is significant heterogeneity among the included studies, with the sources of this heterogeneity remaining unclear. Second, considerable differences exist in the confounders adjusted across studies, which may compromise the reliability of the results. Some studies adjusted only for age and sex, while important potential risk factors such as educational level, history of CVD, alcohol intake were not considered. In addition, the small sample size in some studies might bias the pooled estimates. Finally, due to limited availability of eligible literatures, the robustness of some outcomes required further validation. In addition, dose-response meta-analysis could not be performed for some outcomes.

## Conclusion

This meta-analysis suggested that ideal CVH status is associated with a reduced risk of all-cause mortality, CVD mortality, total cancer mortality and incident CVD. It also demonstrated a trend of lower risk of several chronic diseases including NAFLD, all cause dementia, etc. And each 10 points increase in CVH can result in substantial reductions in risk of all-cause mortality, CVD mortality, incident CVD and all-cause dementia. There's a linear dose-response relationship between CVH score and all-cause mortality, total cancer mortality and a nonlinear dose-response relationship between CVH score and CVD mortality, incident CVD, NAFLD, stroke, myocardial infarction.

## Data Availability

The original contributions presented in the study are included in the article/Supplementary Material, further inquiries can be directed to the corresponding author.
