# Supplementary material for: A systematic review and meta-analysis of the association between cardiovascular health determined by life's essential 8 and risk of mortality and major non-communicable diseases
Source: Front Cardiovasc Med. 2025 Oct 23;12:1612056. doi: 10.3389/fcvm.2025.1612056 (PMC12590506; doi:10.3389/fcvm.2025.1612056)
Supplement: Supplementary file 1 [file Datasheet1.pdf]

Table S1. Search strategy for each database

No	Databases	Search terms	Search Results
1	PubMed	#1<br>((cardiovascular health metrics [Title/Abstract]) OR (ideal cardiovascular health[Title/Abstract]) OR (CVH [Title/Abstract]) OR (life’s essential 8 [Title/Abstract]) OR (LE8 [Title/Abstract]))<br>#2<br>((mortality [Title/Abstract]) OR (all-cause mortality[Title/Abstract]) OR (death [Title/Abstract]) OR (cardiovascular disease* [Title/Abstract]) OR (stroke [Title/Abstract]) OR (cerebrovascular disease*[Title/Abstract]) OR(coronary heart disease*[Title/Abstract]) OR (cancer*[Title/Abstract]) OR (dementia [Title/Abstract]) OR (chronic kidney disease [Title/Abstract]) OR (frailty [Title/Abstract]) OR (depression [Title/Abstract]) OR (diabetes[Title/Abstract]) OR(non-alcoholic fatty liver disease[Title/Abstract]))<br>#3<br>((prospective[Title/Abstract]) OR (cohort[Title/Abstract]) OR (longitudinal[Title/Abstract]) OR(follow-up[Title/Abstract]))<br>#1AND #2 AND#3	496
2	Embase	#1<br>('cardiovascular health metrics':ab,ti OR 'ideal cardiovascular health':ab,ti OR 'CVH':ab,ti OR 'life’s essential 8':ab,ti OR 'LE8':ab,ti)<br>**#2**<br>('mortality':ab,ti OR 'all-cause mortality':ab,ti OR 'death':ab,ti OR 'cardiovascular disease*':ab,ti OR 'stroke':ab,ti OR 'cerebrovascular disease*':ab,ti OR 'coronary heart disease*':ab,ti OR 'cancer*':ab,ti OR 'dementia':ab,ti OR 'chronic kidney disease':ab,ti OR 'frailty':ab,ti OR 'depression':ab,ti OR 'diabetes':ab,ti OR 'non-alcoholic fatty liver disease':ab,ti)<br>#3<br>('prospective':ab,ti OR 'cohort':ab,ti OR 'longitudinal':ab,ti OR 'follow-up':ab,ti)<br>#1AND #2 AND #3	708
3	Web of Science	#1<br>((((TS=(cardiovascular health metrics)) OR TS=(ideal cardiovascular health)) OR TS=(CVH)) OR TS=(life’s essential 8)) OR TS=(LE8)<br>#2<br>((((((((((((TS=(mortality)) OR TS=(all-cause mortality)) OR	4113

		TS=(death)) OR TS=(cardiovascular disease*)) OR TS=(stroke)) OR TS=(cerebrovascular disease*)) OR TS=(coronary heart disease*)) OR TS=(cancer*)) OR TS=(dementia)) OR TS=(chronic kidney disease)) OR TS=(frailty)) OR TS=(depression)) OR TS=(diabetes)) OR TS=(non-alcoholic fatty liver disease)<br>#3<br>(((TS=(prospective)) OR TS=(cohort)) OR TS=(longitudinal)) OR TS=(follow-up)<br>#1AND #2 AND #3	
4	Scopus	#1<br>TITLE-ABS-KEY("cardiovascular health metrics") OR TITLE-ABS-KEY("ideal cardiovascular health") OR TITLE-ABS-KEY("CVH") OR TITLE-ABS-KEY("life's essential 8") OR TITLE-ABS-KEY("LE8")<br>**#2**<br>TITLE-ABS-KEY("mortality") OR TITLE-ABS-KEY("all-cause mortality") OR TITLE-ABS-KEY("death") OR TITLE-ABS-KEY("cardiovascular disease*") OR TITLE-ABS-KEY("stroke") OR TITLE-ABS-KEY("cerebrovascular disease*") OR TITLE-ABS-KEY("coronary heart disease*") OR TITLE-ABS-KEY("cancer*") OR TITLE-ABS-KEY("dementia") OR TITLE-ABS-KEY("chronic kidney disease") OR TITLE-ABS-KEY("frailty") OR TITLE-ABS-KEY("depression") OR TITLE-ABS-KEY("diabetes") OR TITLE-ABS-KEY("non-alcoholic fatty liver disease")<br>**#3**<br>TITLE-ABS-KEY("prospective") OR TITLE-ABS-KEY("cohort") OR TITLE-ABS-KEY("longitudinal") OR TITLE-ABS-KEY("follow-up")<br>#1AND #2 AND #3	690

Table S2. Quality assessment of relevant studies according to Newcastle-Ottawa Scale

		Selection			Comparability	Outcome			Stars
First author, year	Respresentiveness of the exposed cohort	Selection of the non-exposed cohort	Ascertainment of exposure	Demonstration that outcome was not present at start of study	Comparability of cohorts on the basis of the design or analysis	Assessment of outcome	Length of follow-up	Adequacy of follow-up	
**All-cause mortality**									
Zhang et al., 2023a[1]	*	*	*	*	**	*	*	*	9
Hernandez-Martinez et al, 2024[2]	*	*	*	*	**	*	*		8
Abramove et al., 2024[3]	*	*	*	*	**	*	*	*	9
Rempakos et al.,2023[4]		*	*	*		*	*		5
Jiang et al., 2024[5]	*	*	*	*	**	*	*		8
Isiozor et al., 2023a [6]	*	*	*	*	**	*	*	*	9
Xue et al.,2024[7]	*	*	*	*	**	*	*	*	9
Carbonneau et al., 2024[8]		*	*	*	**	*	*		7
Ma et al., 2023[9]	*	*	*	*	**	*	*	*	9
Sun al., 2023a[10]	*	*	*	*	**	*	*	*	9
Sun et al.,2023b[11]	*	*	*	*	**	*	*	*	9
Yi et al., 2023[12]	*	*	*	*	**	*	*	*	9
Kaur et al., 2024 [13]	*	*	*	*	**	*			7
Xing et al., 2023[14]	*	*	*	*	**	*	*	*	9
Guo et al.,2025a[15]	*	*	*	*	**	*	*	*	9
Pu et al.,2025[16]	*	*	*	*	**	*	*	*	9
Ning et al.,2024[17]	*	*	*	*	**	*	*	*	9
**CVD mortality**									

Zhang et al., 2023a[1]	*	*	*	*	**	*	*	*	9
Hernandez-Martinez et al, 2023[2]	*	*	*	*	**	*	*		8
Abramov et al., 2024[3]	*	*	*	*	**	*	*	*	9
Isiozor et al.,2023a[6]	*	*	*	*	**	*	*	*	9
Xue et al.,2024[7]	*	*	*	*	**	*	*	*	9
Carbonneau et al., 2024[8]		*	*	*	**	*	*		7
Ma et al., 2023[9]	*	*	*	*	**	*	*	*	9
Sun al., 2023a[10]	*	*	*	*	**	*	*	*	9
Yi et al., 2023[12]	*	*	*	*	**	*	*	*	9
Kaur et al.,2024[13]	*	*	*	*	**	*			7
Pu et al.,2025[16]	*	*	*	*	**	*	*	*	9
Ning et al.,2024[17]	*	*	*	*	**	*	*	*	9
**Total cancer mortality**									
Abramove et al., 2024[3]	*	*	*	*	**	*	*	*	9
Lin et al.,2024[18]	*	*	*	*	**	*	*	*	9
Ning et al.,2024[17]	*	*	*	*	**	*	*	*	9
**Total cancer incidence**									
Yu et al., 2024[19]		*	*	*	**	*	*		7
Jiang et al., 2024[5]		*	*	*	**	*	*	*	8
**CVD incidence**									
Rempakos et al., 2023[4]		*	*	*		*	*		5
Zhang et al., 2023a[1]	*	*	*	*	**	*	*	*	9
Xia et al.,2023[20]	*	*	*	*	**	*	*	*	9
Paing et al., 2024[21]	*	*	*	*	**	*	*	*	9

Jin et al.,2023[22]	*	*	*	*	**	*	*	*	9
Isiozor et al.,2023b[23]	*	*	*	*	**	*	*	*	9
Carbonneau et al., 2024[8]		*	*	*	**	*	*		7
Li et al., 2023[24]	*	*	*	*	**	*	*		8
Xing et al., 2023[14]	*	*	*	*	**	*	*	*	9
Guo et al.,2025a[25]	*	*	*	*	**	*	*	*	9
**Stroke incidence**									
Paing et al., 2024[21]	*	*	*	*	**	*	*	*	9
Xia et al.,2023[20]	*	*	*	*	**	*	*	*	9
Zhang et al.,2023a[1]	*	*	*	*	**	*	*	*	9
Wu et al.,2023[26]	*	*	*	*	**	*	*		8
Xing et al2023[14]	*	*	*	*	**	*	*	*	9
Li et al.,2023[24]	*	*	*	*	**	*	*		8
Guo et al.,2025a[25]	*	*	*	*	**	*	*	*	9
**Myocardial infarction incidence**									
Paing et al., 2024[21]	*	*	*	*	**	*	*	*	9
Zhang et al.,2023a[1]	*	*	*	*	**	*	*	*	9
Xing et al2023[14]	*	*	*	*	**	*	*	*	9
**Heart Failure incidence**									
Cai et al.,2024[27]	*	*	*	*	**	*	*	*	9
Paing et al., 2024[21]	*	*	*	*	**	*	*	*	9
Xing et al2023[14]	*	*	*	*	**	*	*	*	9
Guo et al.,2025a[25]	*	*	*	*	**	*	*	*	9
**CHD incidence**									
Paing et al., 2024[21]	*	*	*	*	**	*	*	*	9

Li et al.,2023[24]	*	*	*	*	**	*	*		8
Guo et al.,2025a[25]	*	*	*	*	**	*	*	*	9
**Atrial fibrillation incidence**									
Xing et al2023[14]	*	*	*	*	**	*	*	*	9
Guo et al.,2025a[25]	*	*	*	*	**	*	*	*	9
Guo et al.,2025b[15]	*	*	*	*	**	*	*		8
Zhang et al.,2023b[28]	*	*	*	*	**	*	*		8
**Hypertension incidence**									
Tian et al.,2023[29]	*	*	*	*	**	*	*		8
Guo et al.,2025a[25]	*	*	*	*	**	*	*	*	9
**Diabetes incidence**									
Tian et al., 2024[30]	*	*	*	*	**	*	*	*	9
Yu et al., 2024[19]	*	*	*	*	**	*	*	*	9
**Non-alcoholic fatty liver disease incidence**									
He et al.,2023[31]	*	*	*	*	**	*	*		8
Huang et al.,2024c[32]	*	*	*	*	**	*			7
Wang et al.,2024b[33]	*	*	*	*	**	*			7
**CKD incidence**									
Ruan et al,2024[34]	*	*	*	*	**	*	*		8
Tang et al,2023[35]	*	*	*	*	**	*	*	*	9
Yu et al.,2024[19]	*	*	*	*	**	*	*	*	9
**Dementia incidence**									
Wang et al.,2024a[36]	*	*	*	*	**	*	*	*	9
Zhou et al.,2023[37]	*	*	*	*	**	*	*	*	9
Yu et al., 2024[19]	*	*	*	*	**	*	*	*	9

Lu et al.,2025[38]	*	*	*	*	**	*	*	*	9
**Alzheimer's disease incidence**									
Zhou et al.,2023[37]	*	*	*	*	**	*	*	*	9
Wang et al.,2024a[36]	*	*	*	*	**	*	*	*	9
**Vascular dementia incidence**									
Zhou et al.,2023[37]	*	*	*	*	**	*	*	*	9
Wang et al.,2024a[36]	*	*	*	*	**	*	*	*	9
**Depression incidence**									
Huang et al., 2024a[39]	*	*	*	*	**	*	*	*	9
Yu et al., 2024[19]	*	*	*	*	**	*	*	*	9
**Anxiety**									
Huang et al., 2024a[39]	*	*	*	*	**	*	*	*	9
Yu et al., 2024[19]	*	*	*	*	**	*	*	*	9
**Asthma incidence**									
Zhang et al., 2024[40]	*	*	*	*	**	*	*	*	9
Yu et al., 2024[19]	*	*	*	*	**	*	*	*	9
**Inflammatory bowel disease incidence**									
Yu et al., 2024[19]	*	*	*	*	**	*	*	*	9
Yang et al.,2024[41]	*	*	*	*	**	*	*	*	9
**Pancreas cancer incidence**									
Yu et al., 2024[19]	*	*	*	*	**	*	*	*	9
Wu et al.,2025[42]	*	*	*	*	**	*	*	*	9
**Venous Thrombeombolism incidence**									
Liang et al.,2024[43]	*	*	*	*	**	*	*	*	9
Isizor et al.,2023b[23]	*	*	*	*	**	*	*	*	9

Table S3 Outcomes excluded for analysis

Author and year	Country/Cohorts	Participant characteristic	Outcome; Case ascertainment; case/participants	Follow-up	Effect estimates （HR）	Covariates
**Myocardial infarction mortality**						
Zhang et al., 2023a[1]	UK, Biobank	Men and women, 40-69 years	MI mortality; NHS Central Register; 603/215675	12.53years	1<br>0.48(0.41-0.57)<br>0.23(0.16-0.34)	Age, sex, ethnicity, education, income, Townsend deprivation index, polygenic risk scores
**Stroke mortality**						
Zhang et al., 2023a[1]	UK, Biobank	Men and women, 40-69 years	Stroke mortality; NHS Central Register; 599/215675	12.53years	1<br>0.81(0.67-0.98)<br>0.54(0.40-0.75)	Age, sex, ethnicity, education, income, Townsend deprivation index, polygenic risk scores
**Breast cancer mortality**						
Zhao et al., 2024[44]	UK, Biobank	Women, 40-69 years	Breast cancer mortality; National cancer and death registries, 418/150566	13.81years	Premenopausal<br>1<br>0.86(0.46-1.60)<br>0.93(0.47-1.83)<br>Postmenopausal<br>1<br>0.90(0.70-1.16)<br>0.57(0.36-0.90)	Age, ethnicity, qualifications, Townsend deprivation index, alcohol consumption, oral contraceptive use, hormone replacement theraphy, number of births, age at menarche, ever had breast cancer mammogram, family history of breast cancer
**Diabetic kidney disease incidence**						
Gao et al., 2023[45]	China, Kailuan Cohort	Men and women, ≥ 18 years	DKD incidence; Medical records; 1798/7605	12.41 years	1<br>0.90(0.81-1.01)<br>0.77(0.69-0.87)	Age, sex, educational level, income per month, occupation, drinking
**Fragility fracture incidence**						
Hou et al., 2024[46]	China, Kailuan Cohort	Men and women, 51.68±12.4 years	Incident fragility fractures; Radiographs, computed tomography scans, or magnetic resonance images; 1534/89464	13.98 years	1<br>0.78(0.66-0.92)<br>0.65(0.51-0.83)	Age, sex, education, alcohol drinking, hemoglobin
**Arterial stiffness incidence**						
Huang et al., 2024b[47]	China, Jiading community cohort study	Men and women, ≥ 40 years	Incident arterial stiffness; Linkage with death register, primary care, and hospital inpatient records; 675/89464	4.3 years	2.89(1.63-5.13)<br>2.29(1.33-3.96)<br>1	Age, sex, alcohol consumption, education, hypoglycemic drugs, anti-hypertensive drugs, lipid-lowering drugs
**Chronic obstructive pulmonary disease incidence**						
Yu et al., 2024[19]	UK, Biobank	Men and women, 55.6±8.1 years	Incident COPD; Linkage with death register, primary care, and hospital inpatient records; 2564/170726	10.85 years	1<br>0.46(0.40-0.53)<br>0.29(0.04-0.06)	Age, sex, ethnicity, education level, Townsend index, alcohol consumption.
**Rheumatoid arthritis incidence**						
Yu et al., 2024[19]	UK, Biobank	Men and women, 55.6±8.1 years	Incident Rheumatoid arthritis; Linkage with death register, primary care, and hospital inpatient records; 1284/170726	10.85 years	1<br>0.61(0.48-0.77)<br>0.48(0.37-0.63)	Age, sex, ethnicity, education level, Townsend index, alcohol consumption.
**Gout incidence**						
Yu et al., 2024[19]	UK, Biobank	Men and women, 55.6±8.1years	Incident gout; Linkage with death register, primary care, and hospital inpatient records; 2461/170726	10.85 years	1<br>0.46(0.40-0.53)<br>0.18(0.15-0.22)	Age, sex, ethnicity, education level, Townsend index, alcohol consumption.
**Ovary cancer incidence**						
Yu et al., 2024[19]	UK, Biobank	Men and women, 55.6±8.1 years	Incident ovary cancer; Linkage with death register, primary care, and hospital inpatient records; 299/170726	10.85 years	1<br>1.16(0.57-2.35)<br>0.86(0.41-1.82)	Age, sex, ethnicity, education level, Townsend index, alcohol consumption.

**Prostate cancer incidence**						
Yu et al., 2024[19]	UK, Biobank	Men and women, 55.6±8.1 years, n=170726	Incident prostate cancer; Linkage with death register, primary care, and hospital inpatient records; 3664/170726	10.85 years	1<br>1.03(0.87-1.22)<br>1.00(0.83-1.21)	Age, sex, ethnicity, education level, Townsend index, alcohol consumption.
**Oropharyngeal cancer incidence**						
Yu et al., 2024[19]	UK, Biobank	Men and women, 55.6±8.1 years, n=170726	Incident oropharyngeal cancer; Linkage with death register, primary care, and hospital inpatient records; 126/170726	10.85 years	1<br>1.00(0.40-2.46)<br>0.77(0.28-2.11)	Age, sex, ethnicity, education level, Townsend index, alcohol consumption.
**Bladder cancer incidence**						
Yu et al., 2024[19]	UK, Biobank	Men and women, 55.6±8.1 years, n=170726	Incident bladder cancer; Linkage with death register, primary care, and hospital inpatient records; 289/170726	10.85 years	1<br>0.58(0.37-0.92)<br>0.48(0.27-0.83)	Age, sex, ethnicity, education level, Townsend index, alcohol consumption.
**Kidney cancer incidence**						
Yu et al., 2024[19]	UK, Biobank	Men and women, 55.6±8.1 years, n=170726	Incident kidney cancer; Linkage with death register, primary care, and hospital inpatient records; 386/170726	10.85 years	1<br>0.58(0.39-0.86)<br>0.41(0.25-0.66)	Age, sex, ethnicity, education level, Townsend index, alcohol consumption.
**Stomach cancer incidence**						
Yu et al., 2024[19]	UK, Biobank	Men and women, 55.6±8.1 years, n=170726	Incident stomach cancer; Linkage with death register, primary care, and hospital inpatient records; 177/170726	10.85 years	1<br>0.59(0.32-1.06)<br>0.36(0.17-0.75)	Age, sex, ethnicity, education level, Townsend index, alcohol consumption.
**Parkinson's disease incidence**						
Yu et al., 2024[19]	UK, Biobank	Men and women, 55.6±8.1 years, n=170726	Incident PD; Linkage with death register, primary care, and hospital inpatient records; 820/170726	10.85 years	1<br>1.35(0.88-2.06)<br>1.41(0.89-2.22)	Age, sex, ethnicity, education level, Townsend index, alcohol consumption.
**Schizophrenia incidence**						
Yu et al., 2024[19]	UK, Biobank	Men and women, 55.6±8.1 years, n=170726	Incident schizophrenia; Linkage with death register, primary care, and hospital inpatient records; 55/170726	10.85 years	1<br>0.73(0.23-2.37)<br>0.39(0.10-1.60)	Age, sex, ethnicity, education level, Townsend index, alcohol consumption.
**Lung cancer incidence**						
Yu et al., 2024[19]	UK, Biobank	Men and women, 55.6±8.1 years, n=170726	Incident lung cancer; Linkage with death register, primary care, and hospital inpatient records; 619/170726	10.85 years	1<br>0.52(0.38-0.70)<br>0.36(0.25-0.53)	Age, sex, ethnicity, education level, Townsend index, alcohol consumption.
**Colorectal cancer incidence**						
Yu et al., 2024[19]	UK, Biobank	Men and women, 55.6±8.1 years, n=170726	Incident colorectal cancer; Linkage with death register, primary care, and hospital inpatient records; 1746/170726	10.85 years	1<br>0.84(0.67-1.05)<br>0.68(0.53-0.88)	Age, sex, ethnicity, education level, Townsend index, alcohol consumption.
**Brain cancer incidence**						
Yu et al., 2024[19]	UK, Biobank	Men and women, 55.6±8.1 years, n=170726	Incident brain cancer; Linkage with death register, primary care, and hospital inpatient records; 254/170726	10.85 years	1<br>1.28(0.60-2.73)<br>1.55(0.70-3.43)	Age, sex, ethnicity, education level, Townsend index, alcohol consumption.
**Liver cancer incidence**						
Yu et al., 2024[19]	UK, Biobank	Men and women, 55.6±8.1 years, n=170726	Incident liver cancer; Linkage with death register, primary care, and hospital inpatient records; 119/170726	10.85 years	1<br>0.48(0.25-0.92)<br>0.36(0.16-0.81)	Age, sex, ethnicity, education level, Townsend index, alcohol consumption.
**Endometrium cancer incidence**						
Yu et al., 2024[19]	UK, Biobank	Men and women,	Incident endometrium cancer;	10.85 years	1	Age, sex, ethnicity, education level,

		55.6±8.1 years, n=170726	Linkage with death register, primary care, and hospital inpatient records; 429/170726		0.37(0.26-0.52)<br>0.25(0.16-0.38)	Townsend index, alcohol consumption.
**Thyroid cancer incidence**						
Yu et al., 2024[19]	UK, Biobank	Men and women, 55.6±8.1 years, n=170726	Incident thyroid cancer; Linkage with death register, primary care, and hospital inpatient records; 133/170726	10.85 years	1<br>0.72(0.31-1.64)<br>0.60(0.24-1.47)	Age, sex, ethnicity, education level, Townsend index, alcohol consumption.
**Mild cognitive impairment**						
Wang et al.,2024a[36]	UK, Biobank	Men and women, 40-69 years, n=126785	Mild cognitive impairment; ICD10., 1797/126785	13.0 years	1<br>0.70(0.61-0.81)<br>0.49(0.40-0.62)	Age, sex, race/ethnicity, years of education, income level, socioeconomic status and APOE e4 status, alcohol status, depression status
**Migraine**						
Lei et al., 2024[48]	UK, Biobank	Men and women, 37-73 years, n=259718	New-onset migraine., ICD10;3225/259718	13.58years	1<br>0.93(0.85-1.02)<br>0.73(0.58-0.92)	Age, sex. Ethnicity, Townsend deprivation, education, income, cardiovascular disease, cancer, other serious disease
**Chronic cardiovascular-kidney disorder**						
Huang et al.,2024d[49]	UK, Biobank	Men and women, 40-69 years	CCKD incidence; linkage from inpatient records, self-reported data, and death registry; 1054/125986	12.5years	1<br>0.46(0.40-0.54)<br>0.25(0.18-0.34)	Age, sex, ethnicity, Deprivation Index, education level, annual household income, number of morbidities, and drinking status.

Table S4. Characteristics of the studies included in the meta-analysis

Author and year	Country/Cohorts	Participant characteristic	Outcome; Case ascertainment; case/participants	Follow-up	Effect estimates （HR）	Covariates	Quality assessment
**All-cause mortality**							
Zhang et al., 2023a[1]	UK, Biobank	Men and women, 40-69 years	All-cause mortality; NHS Central Register; 10257/197473	12.53years	1<br>0.64(0.61-0.67)<br>0.45(0.42-0.49)	Age, sex, ethnicity, education, Townsend index, income, polygenic risk scores	9
Hernandez-Martinez et al, 2024[2]	Spain, ENRICA	Men and women, ≥18 years	All-cause mortality; NDI; 908/11616	12.9 years	1<br>0.68(0.56-0.83)<br>0.63(0.51-0.78)<br>0.53(0.39-0.72)	Age, sex, social class, educational level, cardiovascular disease and cancer	8
Abramov et al., 2024[3]	USA, NHANES	Men and women; >18 years	All-cause mortality; NDI; 1760/20215	10 years	1<br>0.63(0.57-0.70)<br>0.35(0.26-0.48)	Age, race and ethnicity, sex, ratio of family income to poverty	9
Rempakos et al., 2023[4]	USA, Framingham Heart Study	Men and women; 44±10 years	All-cause mortality; NDI; 1195/2888	33 years	1<br>0.51(0.44-0.60)<br>0.36(0.28-0.46)	Age, sex	5
Jiang et al.,2024[5]	China, Kailuan Cohort	Men and women, 51.42±12.46 years	All-cause mortality; Provincial vital statistics offices; 12807/94733	14years	1.71(1.56-1.87)<br>1.46(1.36-1.58)<br>1	Age, gender, education level, occupation, drinking, family history of cancer, history of cardiovascular diseases	8
Isiozor et al.,2023a[6]	Finland, Kuopio Ischaemic Heart Disease study	Men, 42-60 years	All-cause mortality; the national death registry; 987/1662	30years	1<br>0.733(0.622-0.863)<br>0.584(0.490-0.697)<br>0.520(0.431-0.626)	Age, alcohol consumption, socio-economic status,	9
Xue et al.,2024[7]	China, CCDRFS	Men and women, ≥18 years	All-cause mortality; the national death registry; 2936/134727	5.2years	1<br>0.68(0.56-0.84)<br>0.57(0.45-0.72)	Age, sex, education levels, marital status, ethnicity, household income per captia, residence, region, drinking	9
Carbonneau et al., 2024[8]	USA, FHS offspring	Men and women, 55.5±13.1	All-cause mortality; the national death registry; 676/5669	14years	2.38(1.73-3.28)<br>1.68(1.21-2.33)<br>1.29(0.92-1.82)<br>1	Age, sex, alcohol use, familial relatedness, prevalent cancer and CVD	7
Ma et al., 2023[9]	USA, NHANES	Men and women; 20-79 years	All-cause mortality; the national death registry; 1359/23003	7.8years	1<br>0.56(0.47-0.66)<br>0.39(0.28-0.53)	Age, sex, race and ethnicity, education, family income, health insurance	9
Sun al., 2023a[11]	USA, NHANES	Men and women; 30-79 years	All-cause mortality; the national death registry; 1805/19951	7.6years	1<br>0.60(0.51-0.71)<br>0.42(0.32-0.56)	Age, sex, race/ethnicity, education level, marital status, ratio of family income poverty, history of heart disease and stroke	9
Sun et al.,2023b[10]	UK, Biobank	Men and women, 37-73 years	Premature mortality; NHS Central Register; 13683/309789	12.7years	1<br>0.59(0.56-0.62)<br>0.42(0.39-0.45)	Sex, age, ethnicity, education, Townsend index, moderate drinking, cardiovascular disease, baseline cancer	9
Yi et al., 2023[12]	USA, NHANES	Men and women; ≥20 years	All-cause mortality; the national death registry; 2942/23110	9.4years	1<br>0.73(0.64-0.84)<br>0.60(0.48-0.75)	Age, sex, race/ethnicity, poverty ratio, education levels, marital status, CVD history, health status, PHQ-9 scores	9
Kaur et al., 2024[13]	USA, NHANES	Men and women; >18 years	All-cause mortality; the national death registry; NA/22761	NA	1<br>0.67(0.61-0.73)<br>0.37(0.29-0.48)	Age, race, poverty index	7
Xing et al., 2023[14]	USA, NHANES	Men and women;18-40 years	All-cause mortality; the national death registry; 219/16011	13years	2.54(1.27-5.06)<br>1.57(0.87-2.84)<br>1	Age, sex, education, income, drinking status	9
Guo et al., 2025a[15]	UK, Biobank	Men and women, 53.12±7.96	All-cause mortality; NHS Central Register; 1183/107682	12.2years	1<br>0.53(0.46-0.60)<br>0.37(0.32-0.43)	Age, sex, household income, ethnic, education levels and alcohol intake	9
Pu et al.,2025[16]	USA, NHANES	Men and women,	All-cause mortality; the NCHS	6.9years	1	Age, sex, race/ethnicity, educational	9

		≥20 years	through linkage with the National Death Index; 1184/18679		0.62(0.52-0.73)<br>0.41(0.28-0.59)	level, poverty-to-income ratio	
Ning et al.,2024[17]	USA, NHANES	Men and women, ≥30 years	All-cause mortality; the NCHS through linkage with the National Death Index; 2036/21062	87months	3.2(2.51-4.09)<br>1.9(1.54-2.34)<br>1	Age, sex, race/ethnicity, education levels, marital status, alcohol intake, diagnosis of CVD,	9
**CVD mortality**							
Zhang et al., 2023a[1]	UK, Biobank	Men and women, 40-69 years	CVD mortality; NHS Central Register; 2074/215675	12.53years	1<br>0.51(0.46-0.56)<br>0.27(0.23-0.32)	Age, sex, ethnicity, education, income, Townsend deprivation index, polygenic risk scores	9
Hernandez-Martinez et al, 2024[2]	Spain, ENRICA	Men and women, ≥18 years	CVD mortality; NDI; 207/11616	11.8 years	1<br>0.62(0.39-0.97)<br>0.55(0.32-0.93)<br>0.38(0.16-0.89)	Age, sex, social class, educational level, cardiovascular disease and cancer	8
Abramov et al., 2024[3]	USA, NHANES	Men and women, >18 years	CVD mortality; NDI; 459/20215	10years	1<br>0.67(0.54-0.82)<br>0.34(0.18-0.64)	Age, race and ethnicity, sex, ratio of family income to poverty	9
Isiozor et al.,2023a[50]	Finland, Kuopio Ischaemic Heart Disease study	Men, 42-60 years	CVD mortality; the national death registry; 402/1662	30years	1<br>0.667(0.518-0.858)<br>0.545(0.416-0.714)<br>0.403(0.298-0.545)	Age, alcohol consumption, socio-economic status,	9
Xue et al.,2024[7]	China, CCDRFS	Men and women, ≥18 years	CVD mortality; the national death registry; 1158/134727	5.2years	1<br>0.60(0.49-0.73)<br>0.40(0.31-0.52)	Age, sex, education levels, marital status, ethnicity, household income per captia, residence, region, drinking	9
Carbonneau., 2024[8]	USA, FHS offspring	Men and women, 55.5±13.1	CVD mortality; the national death registry; 153/5669	14years	4.68(2.42-9.05)<br>2.79(1.41-5.52)<br>2.98(1.49-5.95)<br>1	Age, sex, alcohol use, familial relatedness	7
Ma et al., 2023[9]	USA, NHANES	Men and women; 20-79 years	CVD mortality; the national death registry; 328/23003	7.8years	1<br>0.56(0.47-0.66)<br>0.39(0.28-0.53)	Age, sex, race and ethnicity, education, family income, health insurance	9
Sun al., 2023a[10]	USA, NHANES	Men and women; 30-79 years	CVD mortality; the national death registry;498 /19951	7.6years	1<br>0.60(0.51-0.71)<br>0.42(0.32-0.56)	Age, sex, race/ethnicity, education level, marital status, ratio of family income poverty, history of heart disease and stroke	9
Yi et al., 2023[12]	USA, NHANES	Men and women; ≥20 years	CVD mortality; the national death registry; 738/23110	9.4years	1<br>0.73(0.64-0.84)<br>0.60(0.48-0.75)	Age, sex, race/ethnicity, poverty ratio, education levels, marital status, CVD history, health status, PHQ-9 scores	9
Kaur et al., 2024[13]	USA, NHANES	Men and women; >18 years	CVD mortality; the national death registry; NA/22761	NA	1<br>0.64(0.54-0.77)<br>0.37(0.21-0.63)	Age, race, poverty index	7
Pu et al.,2025[16]	USA, NHANES	Men and women; ≥20 years	CVD mortality; the NCHS through linkage with the National Death Index; 276/18679	6.9years	1<br>0.58(0.39-0.86)<br>0.41(0.20-0.84)	Age, sex, race/ethnicity, educational level, poverty-to-income ratio	9
Ning et al.,2024[17]	USA, NHANES	Men and women, ≥30 years	CVD mortality; the NCHS through linkage with the National Death Index; 778/21062	87months	4.31(2.6-7.13)<br>2.37(1.64-3.42)<br>1	Age, sex, race/ethnicity, education levels, marital status, alcohol intake, diagnosis of CVD	9
**Total Cancer mortality**							
Abramov et al., 2024[3]	USA, NHANES	Men and women, >18 years	Cancer mortality; NDI; 353/ 20215	10years	1<br>0.72(0.56-0.91)<br>0.50(0.29-0.89)	Age, race and ethnicity, sex, ratio of family income to poverty	9
Lin et al.,2024[18]	USA, NHANES	Men and women, >18 years	Cancer mortality; NDI; 424/ 17076	8.3years	1<br>0.81(0.64-1.02)<br>0.58(0.37-0.91)	Age, sex, race/ethnicity, educational level, Townsend deprivation index, Charlson comorbidity index	9
Lin et al.,2024[18]	UK, Biobank	Men and women, >18 years	Cancer mortality; NDI; 8872/ 272727	13.5years	1<br>0.65(0.60-0.70)<br>0.51(0.46-0.57)	Age, sex, race/ethnicity, educational level, Townsend deprivation index, Charlson comorbidity index, the first ten principal components of ancestry	9

Ning et al.,2024[17]	USA, NHANES	Men and women, ≥30 years	Cancer mortality; the NCHS through linkage with the National Death Index;503/ 21062	87months	1.95(1.25-3.03)<br>1.63(1.09-2.43)<br>1	Age, sex, race/ethnicity, education levels, marital status, alcohol intake, diagnosis of CVD	9
**Total Cancer incidence**							
Yu et al., 2024[19]	UK, Biobank	Men and women, 55.6±8.1 years	Total cancer incidence; NDI;13906 /170726	10.85 years	1<br>0.82(0.75-0.89)<br>0.73(0.67-0.80)	Age, sex, ethnicity, education level, Townsend index, alcohol consumption	7
Jiang et al., 2024[5]	China, Kailuan	Men and women, 51.42±12.4 years	Total cancer incidence; Municipal Social Insurance Institution and Hospital Discharge Register; 5060/ 94733	14 years	1.27(1.11-1.45)<br>1.26(1.14-1.39)<br>1	Age, gender, education level, occupation, drinking, family history of cancer, history of cardiovascular diseases	8
**CVD incidence**							
Rempakos et al., 2023[4]	USA, Framingham Heart Study	Men and women; 44±10 years	CVD incidence; Medical records; 966/2888；	33 years	1<br>0.47(0.40-0.56)<br>0.21(0.15-0.28)	Age, sex	5
Zhang et al., 2023a[1]	UK, Biobank	Men and women, 40-69 years	CVD incidence; The primary care system, hospital inpatient records, and death registry; 71774/215675	12.53years	1<br>0.65(0.64-0.66)<br>0.40(0.39-0.41)	Age, sex, ethnicity, education, income, Townsend deprivation index, polygenic risk scores	9
Xia et al.,2023[20]	China, Kailuan Cohort	Men and women, 50.9±12.3 years	CVD incidence; The biennial face-to face interviews, medical record from the municipal social insurance institution, the discharge summaries and the death certificates; 9977/89755	NA	1<br>0.57(0.54-0.61)<br>0.33(0.29-0.37)	Age, sex	9
Paing et al., 2024[21]	USA, SHFS	Men and women, 34.6±16.3 years	CVD incidence; ECG and positive Rose Angina Questionnaire, Medical records; 274/2139	19.34 years	1<br>0.52(0.40-0.68)<br>0.25(0.14-0.44)	Age, sex, education, study site	9
Jin et al.,2023[22]	China, China-PAR cohort	Men and women, China- PAR 51.3±11.9 years	CVD incidence; Medical records; 4533/88665	10 years	1<br>0.83(0.77-0.90)<br>0.68(0.63-0.74)<br>0.50(0.46-0.55)<br>0.38(0.34-0.42)	Age, sex, family history of atherosclerotic cardiovascular disease, marital status, education levels, average family income	9
Isiozor et al.,2023b[23]	Finland, Kuopio Ischaemic Heart Disease study	Men, 42-61 years	ASCVD incidence; National Hospital Discharge Registry Data; 889/1899	24 years	1<br>0.69(0.58-0.83)<br>0.57(0.48-0.69)<br>0.42(0.34-0.51)	Age, alcohol consumption, socioeconomic status and family history of coronary heart disease	9
Carbonneau et al., 2024[8]	USA, FHS offspring	Men and women, 55.5±13.1years	CVD incidence; the national death registry; 405/5669	14 years	3.10(2.20-4.36)<br>2.02(1.42-2.88)<br>1.73(1.20-2.51)<br>1	Age, sex, alcohol use, familial relatedness	7
Xing et al., 2023[14]	USA, NHANES	Men and women; 18-40years	CVD incidence; the national death registry; 271/16011	13years	7.34(3.19-16.89)<br>4.17(1.93-9.02)<br>1	Age, sex, education, income, drinking status	9
Li et al.,2023[24]	UK, Biobank	Men and women; 37-73years	CVD incidence; the national death registry; 8595/137794	10years	1<br>0.60(0.56-0.64)<br>0.36(0.33-0.40)	Age, sex, race, assessment center, Townsend index, alcohol intake, sedentary behaviour, average household income, education	8
Guo et al.,2025a[25]	UK, Biobank	Men and women, 53.12±7.96	CVD incidence; death certificates provided by the National Health Service (NHS) Information Centre (England and Wales) and the NHS Central Register Scotland (Scotland);7243/107682	12.2years	1<br>0.57(0.51-0.63)<br>0.42(0.38-0.47)	Age, sex, household income, ethnic, education levels and alcohol intake	9
**Stroke incidence**							
Wu et al.,2023[26]	China, Kailuan Cohort	Men and women, NA years	Stroke incidence; biennial questionnaire, the Municipal Social Insurance Institution; 1750/68854	5.65years	1<br>0.68(0.61-0.76)<br>0.33(0.20-0.54)	Age, sex, family history of myocardial infarction and stroke, waist circumference, heart rate, high-	8

						sensitivity C-reactive protein, estimated glomerular filtration rate, alcohol consumption, educational level, occupation, monthly salary	
Paing et al.,2024[21]	USA, SHFS	Men and women, 34.6±16.3 years	Stroke incidence; Medical records and death certificates; 53/2139	19.34 years	1<br>0.59(0.33-1.04)<br>0.57(0.23-1.40)	Age, sex, education, study site	9
Xia et al.,2023[20]	China, Kailuan Cohort	Men and women, 50.9±12.3 years	Stroke incidence; The biennial face-to face interviews, medical record from the municipal social insurance institution, the discharge summaries and the death certificates; 6031/89755	NA	1<br>0.57(0.55-0.60)<br>0.43(0.40-0.46)	Age, sex	9
Zhang et al.,2023a[1]	UK, Biobank	Men and women, 40-69 years	Stroke incidence; The primary care system, hospital inpatient records, and death registry;5672 /215675	12.53years	1<br>0.66(0.62-0.70)<br>0.53(0.48-0.58)	Age, sex, ethnicity, education, income, Townsend deprivation index, polygenic risk scores	9
Li et al., 2023[24]	UK, Biobank	Men and women; 37-73years	Stroke incidence; the national death registry; 1948/137794	10years	1<br>0.65(0.57-0.75)<br>0.45(0.37-0.54)	Age, sex, race, assessment center, Townsend index, alcohol intake, sedentary behaviour, average household income, education	8
Xing et al.,2023[14]	USA, NHANES	Men and women; 18-40years	Stroke incidence; the national death registry; 185/16011	13years	15.6(3.59-67.78)<br>10.78(2.63-44.13)<br>1	Age, sex, education, income, drinking status	9
Guo et al.,2025a[25]	UK, Biobank	Men and women, 53.12±7.96	Stroke incidence; death certificates provided by the National Health Service (NHS) Information Centre (England and Wales) and the NHS Central Register Scotland (Scotland);NA/107682	12.2years	1<br>0.66(0.49-0.89)<br>0.53(0.39-0.73)	Age, sex, household income, ethnic, education levels and alcohol intake	9
**Venous Thrombeombolism incidence**							
Liang et al.,2024[43]	UK, Biobank	Men and women, 56.12±8.11 years	Venous thromboembolism incidence; hospital admission records, primary care records, self-reports, and death record; 7295/275149	12.56years	1<br>0.77(0.71-0.85)<br>0.59(0.52-0.66)	Age, sex, ethnicity, education, Townsend deprivation index, alcohol consumption, laboratory tests, medication intake, comorbidities, polygenic risk score	9
Isizor et al.,2023b[23]	Finland, KIHD prospective study	Men and women, 42-61 years	Venous thromboembolism incidence; linkages to National Hospital Discharge Registry Data maintained by the Finnish Institute for Health and Welfare; 127/1899	25years	1<br>0.94(0.55-1.60)<br>1.46(0.89-2.39)<br>1.01(0.59-1.72)	Age, alcohol consumption, socioeconomic status and family history of coronary heart disease	9
**Myocardial infarction incidence**							
Paing et al.,2024[21]	USA, SHFS	Men and women, 34.6±16.3 years	Myocardial infarction incidence; Medical records and death certificates; 144/2139	20 years	1<br>0.45(0.28-0.73)<br>0.09(0.03-0.37)	Age, sex, education, study site	9
Zhang et al.,2023a[1]	UK, Biobank	Men and women, 40-69 years	Myocardial infarction incidence; The primary care system, hospital inpatient records, and death registry;7893 /215675	12.53years	1<br>0.57(0.54-0.6)<br>0.31(0.28-0.34)	Age, sex, ethnicity, education, income, Townsend deprivation index, polygenic risk scores	9
Xing et al.,2023[14]	USA, NHANES	Men and women; 18-40years	Myocardial infarction incidence; the national death registry; 44/16011	13years	8.03(0.98-65.96)<br>2.86(0.38-21.61)<br>1	Age, sex, education, income, drinking status	9
**Heart Failure incidence**							
Paing et al.,2024[21]	USA, SHFS	Men and women, 34.6±16.3 years	Heart failure incidence; Medical records and death certificates; 164/2139	20 years	1<br>0.74(0.24-1.23)<br>0.26(0.08-0.87)	Age, sex, education, study site	9

Xing et al.,2023[14]	USA, NHANES	Men and women; 18-40years	Heart failure incidence; the national death registry; 36/16011	13years	3.39(0.58-19.83)<br>2.09(0.46-9.47)<br>1	Age, sex, education, income, drinking status	9
Cai et al.,2024[27]	China, PEACE Million Persons Project	Men and women; 35-75years	Heart failure incidence; linking hospital records with participants identity card number 291/38571	3.56years	1<br>0.52(0.43-0.61)<br>0.38(0.26-0.57)	Age, sex,drinking status, education, annual income, insurance status, urbanity and occupation	9
Guo et al.,2025a[25]	UK, Biobank	Men and women, 53.12±7.96	Stroke incidence; death certificates provided by the National Health Service (NHS) Information Centre (England and Wales) and the NHS Central Register Scotland (Scotland);NA/107682	12.2years	1<br>0.54(0.42-0.71)<br>0.36(0.27-0.48)	Age, sex, household income, ethnic, education levels and alcohol intake	9
**Coronary heart disease incidence**							
Paing et al.,2024[21]	USA, SHFS	Men and women, 34.6±16.3 years	Coronary heart disease incidence; Medical records and death certificates; 121/2139	Age, sex, education, study site	1<br>0.48(0.35-0.66)<br>0.21(0.11-0.42)	Age, sex, education, study site	9
Li et al., 2023[24]	UK, Biobank	Men and women; 37-73years	Coronary heart disease; the national death registry; 6968/137794	10years	1<br>0.52(0.49-0.56)<br>0.29(0.26-0.32)	Age, sex, race, assessment center, Townsend index, alcohol intake, sedentary behaviour, average household income, education	8
Guo et al.,2025a[25]	UK, Biobank	Men and women, 53.12±7.96	Coronary heart disease incidence; death certificates provided by the National Health Service (NHS) Information Centre (England and Wales) and the NHS Central Register Scotland (Scotland);NA/107682	12.2years	1<br>0.49(0.43-0.56)<br>0.32(0.27-0.36)	Age, sex, household income, ethnic, education levels and alcohol intake	9
**Atrial fibrillation incidence**							
Xing et al.,2023[14]	USA, NHANES	Men and women; 18-40years	Atrial fibrillation incidence; the national death registry; 14/16011	13years	3.25(0.42-25.04)<br>0.69(0.13-3.78)<br>1	Age, sex, education, income, drinking status	9
Guo et al.,2025a[25]	UK, Biobank	Men and women, 53.12±7.96	Coronary heart disease incidence; death certificates provided by the National Health Service (NHS) Information Centre (England and Wales) and the NHS Central Register Scotland (Scotland);NA/107682	12.2years	1<br>0.92(0.73-1.17)<br>0.85(0.67-1.09)	Age, sex, household income, ethnic, education levels and alcohol intake	9
Guo et al.,2025b[15]	UK, Biobank	Men and women; 40-69years	Atrial fibrillation incidence; information on arrhythmia-related death 40 from the National Health Service Information Centre; 16802/287264	12.8years	1<br>0.72(0.69-0.76)<br>0.63(0.59-0.68)	Age, sex, ethnicity, drinking status, education, and Townsend deprivation index	8
Zhang et al.,2023b[28]	UK, Biobank	Men and women; 40-69years	Atrial fibrillation incidence; iusing the primary care system, hospital inpatient records, and death registry; 14743/250898	12.46years	1<br>0.76(0.73-0.79)<br>0.66(0.62-0.70)	Age, sex, ethnicity, education, income, binge drinking, COPD, CRF, MI, stroke, HF, polygenic risk scores (PRS), LE8 and PM2.5	8
**Hypertension incidence**							
Tian et al., 2023[29]	China, Kailuan Cohort	Men and women, ≥ 18 years, n=52990	Hypertension incidence; A BP measurement ≥140/90 mm Hg, any use of antihypertensive medication, or a self-reported history of hypertension; 28380/52990	10.73years	1<br>0.82(0.79-0.86)<br>0.54(0.51-0.57)	Age, sex, education level, income, drinking status	8
Guo et al	UK, Biobank	Men and women,	Hypertension incidence; death	12.2years	1	Age, sex, household income, ethnic,	9

al.,2025a[25]		53.12±7.96	certificates provided by the National Health Service (NHS) Information Centre (England and Wales) and the NHS Central Register Scotland (Scotland);NA/107682		0.48(0.43-0.53)<br>0.26(0.23-0.29)	education levels and alcohol intake	
**Diabetes incidence**							
Tian et al., 2024[30]	China, Kailuan Cohort	Men and women, ≥18 years	Diabetes incidence; Using antidiabetic medication or having FBG≥7.0 mmol/L; 13097/86149	12.94years	1<br>0.67(0.64-0.70)<br>0.27(0.23-0.31)	Age, sex, education level, income, drinking status, estimated glomerular filtration rate, high sensitivity C-reactive protein	9
Yu et al., 2024[19]	UK, Biobank	Men and women, 55.6±8.1 years	Diabetes incidence; Linkage with death register, primary care, and hospital inpatient records; 4984 /170726	10.85 years	1<br>0.23(0.21-0.25)<br>0.05(0.04-0.06)	Age, sex, ethnicity, education level, Townsend deprivation index, alcohol consumption.	9
**CKD incidence**							
Ruan et al.,2024[34]	UK, Biobank	Men and women, 56.4(50-62) years	CKD incidence; cases were obtained from records linked to inpatients, death register, and primary care or presenting with decreased kidney function, defined by eGFR; 10124/251825	12.8years	1<br>0.560(0.603-0.724)<br>0.531(0.460-0.594)	Age, sex, ethnicity, education, Townsend deprivation index, average household income, alcohol frequency intake, history of CVD, hypertension and diabetes, CRP, albumin, low-density lipoprotein, triglyceride, history of medication for cholesterol lowering, insulin, antihypertensive and NSAIDS	8
Tang et al,2023[35]	UK, Biobank	Men and women, 37-73 years	CKD incidence; Linkage with death register, primary care, and hospital inpatient records; 1936/147988	10.0years	1<br>0.61(0.52-0.72)<br>0.43(0.35-0.53)	Age, ethnicity, sex, Townsend deprivation index, education, average household income, CRP, cancer at baseline	9
Yu et al.,2024[19]	UK, Biobank	Men and women, 55.6±8.1 years	CKD incidence; Linkage with death register, primary care, and hospital inpatient records; 10124/251825	10.85 years	1<br>0.52(0.46-0.58)<br>0.32(0.27-0.37)	Age, sex, ethnicity, education level, Townsend index, alcohol consumption.	9
**Non-alcoholic fatty liver disease incidence**							
He et al.,2023[31]	UK, Biobank	Men and women, 40-70 years	NAFLD incidence; An expert panel consensus statement about administrative coding in electronic health care record-based research of NAFLD; 2284/266645	11.9 years	1<br>0.43(0.39-0.48)<br>0.1(0.07-0.14)	Age, sex, race, education levels, Townsend deprivation index, alcohol intake, use of antidiabetic drugs and cholesterol-lowering drugs	8
Huang et al.,2024c[32]	China, Jiading community cohort study	Men and women, ≥40 years	NAFLD incidence; Ultrasonography; 623/3266	4.3 years	OR value<br>2.56(1.55-4.24)<br>1.83(1.17-2.85)<br>1	Age, sex, alcohol consumption, education, TG, LDL-C, HOMA-IR	7
Wang et al.,2024b[33]	China, NAFLD development cohort	Men and women, 39.4±12.6 years	NAFLD incidence; Ultrasonography; 3510/21844	2.3 years	1<br>0.87(0.80-0.96)<br>0.76(0.69-0.83)<br>0.58(0.53-0.65)	Age, sex, education level, drinking status, waist circumference, eGFR, ALT, antidiabetic medication, lipid-lowering, antihypertensive medication	7
**All-cause dementia incidence**							
Wang et al.,2024a[36]	UK, Biobank	Men and women, 40-69 years	Dementia incidence; linked data from hospital admissions and death registries; 1055/126785	13.0years	1<br>0.79 (0.65-0.96)<br>0.60 (0.44-0.80)	Age, sex, race/ethnicity, years of education, income level, socioeconomic status and APOE e4 status, alcohol status and depression status	9
Zhou et al.,2023[37]	UK, Biobank	Men and women, 37-73 years	Dementia incidence; a combination of primary/ secondary diagnosis (hospital inpatient records) or underlying/ contributory cause of death (death register) in accordance with the International Classification	12.6years	1<br>0.62(0.55-0.71)<br>0.56(0.48-0.64)	Age, sex, ethnicity, socioeconomic status, education, household income, alcohol consumption frequency, sedentary time, family history of dementia, depression, Charlson comorbidity index	9

			of Diseases coding system or self-report; 4238/316669				
Yu et al.,2024[19]	UK, Biobank	Men and women, 55.6±8.1 years	Dementia incidence; Linkage with death register, primary care, and hospital inpatient records; 1421/170726	10.85 years	1<br>0.80(0.62-1.03)<br>0.76(0.57-1.01)	Age, sex, ethnicity, education level, Townsend index, alcohol consumption	9
Lu et al.,2025[38]	UK, Biobank	Men and women, 40-69 years	Dementia incidence; Data from hospital admissions, primary care records, and/or death registries; 2762/238420	12.7years	Male<br>1<br>0.79 (0.65-0.96)<br>0.70 (0.53-0.92)<br>Female<br>1<br>0.88 (0.70-1.10)<br>0.75 (0.58-0.98)	Age, ethnicity, education level, employment status, total family income and Townsend deprivation index	9
**Alzheimer's disease incidence**							
Wang et al.,2024a[36]	UK, Biobank	Men and women, 40-69 years	Alzheimer's disease incidence; Linked data from hospital admissions and death registries; 441/126785	13.0years	1<br>0.99 (0.71-1.37)<br>0.79 (0.50-1.27)	Age, sex, race/ethnicity, years of education, income level, socioeconomic status and APOE e4 status, alcohol status and depression status	9
Zhou et al.,2023[37]	UK, Biobank	Men and women, 37-73 years	Alzheimer's disease incidence; A combination of primary/ secondary diagnosis (hospital inpatient records) or underlying/ contributory cause of death (death register) in accordance with the International Classification of Diseases coding system or self-report; 1797/316669	12.6years	1<br>0.84 (0.67-1.05)<br>0.84 (0.65-1.07)	Age, sex, ethnicity, socioeconomic status, education, household income, alcohol consumption frequency, sedentary time, family history of dementia, depression, Charlson comorbidity index	9
**Vascular dementia incidence**							
Wang et al.,2024a[36]	UK, Biobank	Men and women, 40-69 years	Vascular dementia incidence; linked data from hospital admissions and death registries; 192/126785	13.0years	1<br>0.78 (0.51-1.20)<br>0.44 (0.21- 0.94)	Age, sex, race/ethnicity, years of education, income level, socioeconomic status and APOE e4 status, alcohol status and depression status	9
Zhou et al.,2023[37]	UK, Biobank	Men and women, 37-73 years	Vascular dementia incidence; A combination of primary/ secondary diagnosis (hospital inpatient records) or underlying/ contributory cause of death (death register) in accordance with the International Classification of Diseases coding system or self-report; 939/316669	12.6years	1<br>0.38 (0.31-0.46)<br>0.29 (0.22-0.38)	Age, sex, ethnicity, socioeconomic status, education, household income, alcohol consumption frequency, sedentary time, family history of dementia, depression, Charlson comorbidity index	9
**Depression incidence**							
Huang et al., 2024a[39]	UK, Biobank	Men and women, 55.7±8.0 years	Depression incidence; Linked hospital admission data, primary care records, self-reported data and death registers;3194 /115855	12.4years	1<br>0.63 (0.57−0.70)<br>0.48 (0.41−0.55)	Age, sex, ethnicity, Deprivation Index, education level, annual household income, number of morbidities, and drinking status.	9
Yu et al., 2024[19]	UK, Biobank	Men and women, 55.6±8.1 years	Depression incidence; Linkage with death register, primary care, and hospital inpatient records; 4590/170726	10.85 years	1<br>0.58(0.51-0.66)<br>0.41(0.36-0.48)	Age, sex, ethnicity, education level, Townsend index, alcohol consumption	9
**Anxiety incidence**							
Huang et al., 2024a	UK, Biobank	Men and women, 55.7±8.0 years	Anxiety incidence; linked hospital admission data, primary care records, self-reported data and death registers;4005 /115855	12.4years	1<br>0.81 (0.73−0.89)<br>0.68 (0.60−0.78)	Age, sex, ethnicity, Deprivation Index, education level, annual household income, number of morbidities, and drinking status.	9
Yu et al., 2024[19]	UK, Biobank	Men and women,	Anxiety incidence; Linkage with	10.85 years	1	Age, sex, ethnicity, education level,	9

		55.6±8.1 years	death register, primary care, and hospital inpatient records; 5064/170726		0.66(0.58-0.74)<br>0.52(0.45-0.60)	Townsend index, alcohol consumption	
**Asthma incidence**							
Zhang et al., 2024[40]	UK, Biobank	Men and women, 40-69 years	Asthma incidence; linkage with hospital admission data and death registry records; 6180/249713	11.6years	1<br>0.72 (0.67- 0.78)<br>0.52 (0.47- 0.58)	Age ,sex, race, education level, family income, Townsend index, alcohol drinking status, PM2.5 exposure, self-reported depression and polygenic risk score	9
Yu et al., 2024[19]	UK, Biobank	Men and women, 55.6±8.1 years	Asthma incidence; Linkage with death register, primary care, and hospital inpatient records; 3870/170726	10.85 years	1<br>0.67(0.58-0.77)<br>0.52(0.45-0.61)	Age, sex, ethnicity, education level, Townsend index, alcohol consumption	9
**Pancreas cancer incidence**							
Yu et al.,2024[19]	UK, Biobank	Men and women, 55.6±8.1 years	Pancreas cancer incidence; Linkage with death register, primary care, and hospital inpatient records; 310/170726	10.85 years	1<br>0.69(0.42-1.14)<br>0.49(0.27-0.87)	Age, sex, ethnicity, education level, Townsend index, alcohol consumption	9
Wu et al.,2025[42]	UK, Biobank	Men and women, 37-73 years	Pancreas cancer incidence; The national cancer registry and hospital records; 905/234102	13.62years	1<br>0.47 (0.39–0.57)<br>0.30 (0.22–0.41)	Age, sex, education level, socioeconomic status, ethnicity and the 10 principal components of ancestry.	9
**Inflammatory bowel disease incidence**							
Yu et al.,2024[19]	UK, Biobank	Men and women, 55.6±8.1 years	Inflammatory bowel disease incidence; Linkage with death register, primary care, and hospital inpatient records; 802/170726	10.85 years	1<br>0.69(0.51-0.93)<br>0.50(0.35-0.70)	Age, sex, ethnicity, education level, Townsend index, alcohol consumption	9
Yang et al.,2024[41]	UK, Biobank	Men and women, 37-73 years	Inflammatory bowel disease incidence; Using inpatient hospital and death registry data; 802/170726	12.3years	1<br>0.82 (0.67, 0.99)<br>0.67 (0.52, 0.83)	Age, sex, Townsend Deprivation Index, drinking status, education levels, depression	9

*Odds ratio with 95%CI

Abbreviations: CVD, cardiovascular disease; CKD, chronic kidney disease; HF, heart failure; HOMA-IR, Homeostatic model assessment of insulin resistance; NAFLD, non-alcoholic fatty liver disease; NDI, national death index; NHS, national health service; TG, triglyceride; LDL-C, low density lipoprotein cholesterol

TableS5. Leave-one-out analysis .

Omitted study	Overall RR	95%CI	Heterogeneity	P for heterogeneity	P for Effect Size
**All-cause mortality**					
Zhang et al, 2023a	0.44	0.39-0.49	I^2=78.9%	＜0.001	＜0.001
Hernandez-Martinez et al, 2023	0.43	0.39-0.48	I^2=78.6%	＜0.001	＜0.001
Abramove et al.2024	0.44	0.40-0.49	I^2=78.1%	＜0.001	＜0.001
Rempakos et al,2023	0.44	0.40-0.49	I^2=77.9%	＜0.001	＜0.001
Jiang et al, 2023	0.43	0.39-0.46	I^2=61.2%	0.001	＜0.001
Isiozor et al, 2023a	0.43	0.39-0.48	I^2=78.2%	＜0.001	＜0.001
Xue et al., 2024	0.43	0.39-0.47	I^2=77.7%	＜0.001	＜0.001
Carbonneau et al.,2024	0.44	0.40-0.48	I^2=78.8%	＜0.001	＜0.001
Ma et al.,2023	0.44	0.40-0.49	I^2=78.6%	＜0.001	＜0.001
Sun et al.,2023	0.44	0.39-0.49	I^2=77.2%	＜0.001	＜0.001
Yi et al.,2023	0.43	0.39-0.47	I^2=76.8%	＜0.001	＜0.001
Kaur et al.,2023	0.44	0.40-0.49	I^2=78.1%	＜0.001	＜0.001
Xing et al.,2023	0.44	0.28-0.60	I^2=78.8%	＜0.001	＜0.001
Guo et al.,2025a	0.44	0.40-0.49	^{2}=76.4%	＜0.001	＜0.001
Pu et al.,2025	0.44	0.40-0.48	I^2=78.8%	＜0.001	＜0.001
Ning et al.,2024	0.45	0.41-0.49	I^2=75.4%	＜0.001	＜0.001
**CVD mortality**					
Zhang et al, 2023a	0.37	0.32-0.42	I^2=0%	0.459	＜0.001
Hernandez-Martinez et al, 2023	0.33	0.28-0.39	I^2=42.2%	0.057	＜0.001
Abramove et al.2024	0.33	0.28-0.40	I^2=42.6%	0.065	＜0.001
Isiozor et al, 2023a	0.33	0.28-0.38	I^2=38.8%	0.128	＜0.001
Xue et al., 2024	0.32	0.27-0.38	I^2=30.1%	0.159	＜0.001
Carbonneau et al.,2024	0.34	0.29-0.30	I^2=36.5%	0.107	＜0.001
Ma et al.,2023	0.34	0.29-0.40	I^2=40.2%	0.081	＜0.001
Sun et al.,2023a	0.33	0.28-0.39	I^2=42.1%	0.069	＜0.001
Yi et al.,2023	0.32	0.28-0.38	I^2=29.1%	0.168	＜0.001
Kaur et al.,2023	0.33	0.28-0.39	I^2=41.9%	0.070	＜0.001
Pu et al.,2025	0.33	0.28-0.39	I^2=41.2%	0.074	＜0.001
Ning et al.,2024	0.34	0.29-0.40	I^2=35.8%	0.112	＜0.001
**Total cancer mortality**					
Abramov et al.,2024	0.51	0.46-0.57	I^2=0.0%	0.862	＜0.001
Lin et al.,2024	0.51	0.46-0.56	I^2=0.0%	0.998	＜0.001
Lin et al.,2024	0.53	0.40-0.70	I^2=0.0%	0.894	＜0.001
Ning et al.,2024	0.51	0.46-0.57	I^2=0.0%	0.858	＜0.001
**CVD incidence**					
Rempakos et al,2023	0.37	0.35-0.40	I^2=68.2%	0.001	＜0.001
Zhang et al, 2023a	0.34	0.31-0.39	I^2=74.5%	＜0.001	＜0.001
Xia et al, 2023b	0.36	0.33-0.40	I^2=75.4%	＜0.001	＜0.001
Paing et al, 2024	0.36	0.33-0.40	I^2=79.1%	＜0.001	＜0.001

Jin et al,2023	0.35	0.32-0.39	I^2=80.2%	＜0.001	＜0.001
Isiozor et al, 2023b	0.35	0.32-0.39	I^2=80.1%	＜0.001	＜0.001
Carbonneau et al.,2024	0.36	0.33-0.40	I^2=79.6%	＜0.001	＜0.001
Xing et al.,2023	0.36	0.33-0.40	I^2=76.9%	＜0.001	＜0.001
Li et al2003	0.36	0.32-0.40	I^2=78.6%	＜0.001	＜0.001
Guo et al.,2025a	0.35	0.31-0.39	I^2=79.6%	＜0.001	＜0.001
**NAFLD incidence**					
He et al, 2023	**0.54**	**0.43-0.68**	**I^2=43.4%**	**0.184**	＜0.001
Huang et al, 2024c	0.24	0.04-1.36	I^2=98.9%	＜0.001	＜0.001
Wang et al, 2024b	0.21	0.06-0.89	I^2=96.7%	＜0.001	＜0.001
**Dementia incidence**					
Wang et al.,2024a	0.66	0.54-0.82	I^2=69.2%	0.039	＜0.001
Zhou et al.,2023	0.71	0.61-0.81	I^2=0.0%	0.470	＜0.001
Yu et al.,2024	0.62	0.52-0.75	I^2=57.7%	0.094	＜0.001
Lu et al.,2025	0.62	0.52-0.74	I^2=42.8%	0.174	＜0.001
**Stroke incidence**					
Paing et al, 2024	0.46	0.39-0.53	I^2=75.6%	0.001	＜0.001
Xia et al.,2023	0.47	0.39-0.57	I^2=57.2%	0.039	＜0.001
Zhang et al.,2023a	0.44	0.37-0.51	I^2=42.6%	0.121	＜0.001
Wu et al.,2023	0.47	0.40-0.55	I^2=73.6%	0.002	＜0.001
Li et al.,2023	0.46	0.38-0.55	I^2=75.7%	0.001	＜0.001
Xing et al.,2023	0.47	0.41-0.53	I^2=66.5%	0.011	＜0.001
Guo et al.,2025a	0.45	0.38-0.53	I^2=74.9%	0.001	＜0.001
**Myocardial infarction incidence**					
Paing et al, 2024	0.31	0.28-0.34	I^2=0.0%	0.345	＜0.001
Zhang et al.,2023a	0.10	0.03-0.28	I^2=0.0%	0.809	＜0.001
Xing et al.,2023	0.20	0.06-0.63	I^2=73.0%	0.054	＜0.001
**HF incidence**					
Paing et al, 2024	0.37	0.29-0.46	I^2=0.0%	0.945	＜0.001
Cai et al.,2024	0.27	0.13-0.54	I^2=41.0%	0.184	＜0.001
Xing et al.,2023	0.33	0.23-0.48	I^2=44.4%	0.165	＜0.001
Guo et al.,2025a	0.27	0.13-0.57	I^2=43.6%	0.170	0. 001
**CKD incidence**					
Ruan et al.,2024	0.37	0.28-0.49	I^2=79.8%	0.026	＜0.001
Tang et al.,2023	0.41	0.25-0.68	I^2=95.8%	＜0.001	＜0.001
Yu et al.,2024	0.49	0.40-0.60	I^2=65.3%	0.090	＜0.001
**Atrial fibrillation incidence**					
Xing et al.,2023	0.67	0.61-0.73	I^2=64.3%	0.061	＜0.001
Zhang et al.,2023b	0.70	0.54-0.92	I^2=65.9%	0.0.053	0. 011
Guo et al.,2025a	0.65	0.62-0.68	I^2=0.0%	0.484	＜0.001
Guo et al.,2025b	0.72	0.57-0.90	I^2=55.1%	0.108	0. 004
**CHD incidence**					
**Paing et al, 2024**	**0.33**	**0.30-0.36**	**I^2=0.0%**	**0.523**	＜0.001

Li et al.,2023	0.20	0.07-0.56	I^2=89.2%	0.002	0．002
Guo et al.,2025a	0.20	0.07-0.61	I^2=90.5%	0.001	0．005

Supplementary Figures

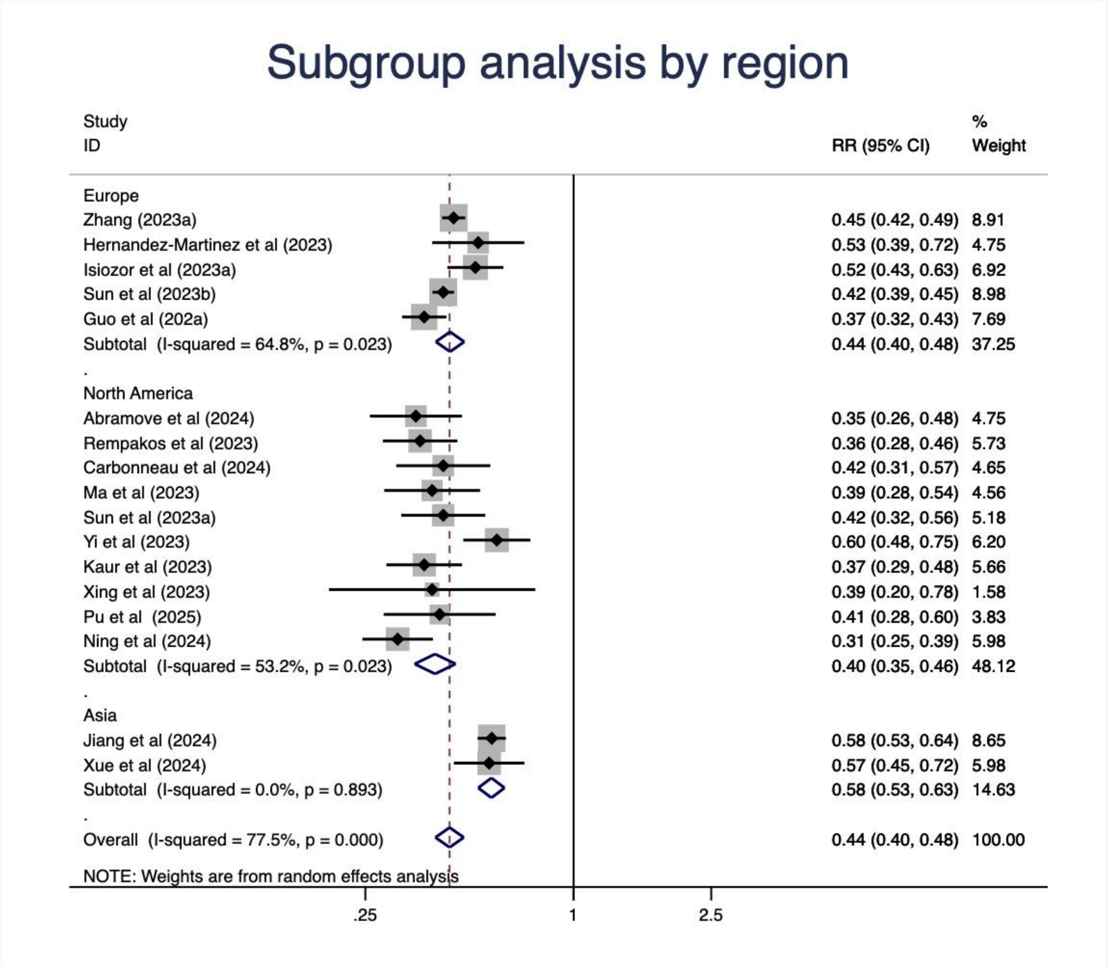

FigureS1. Forest plot of subgroup analysis by region for the association between low and ideal CVH with all-cause mortality.

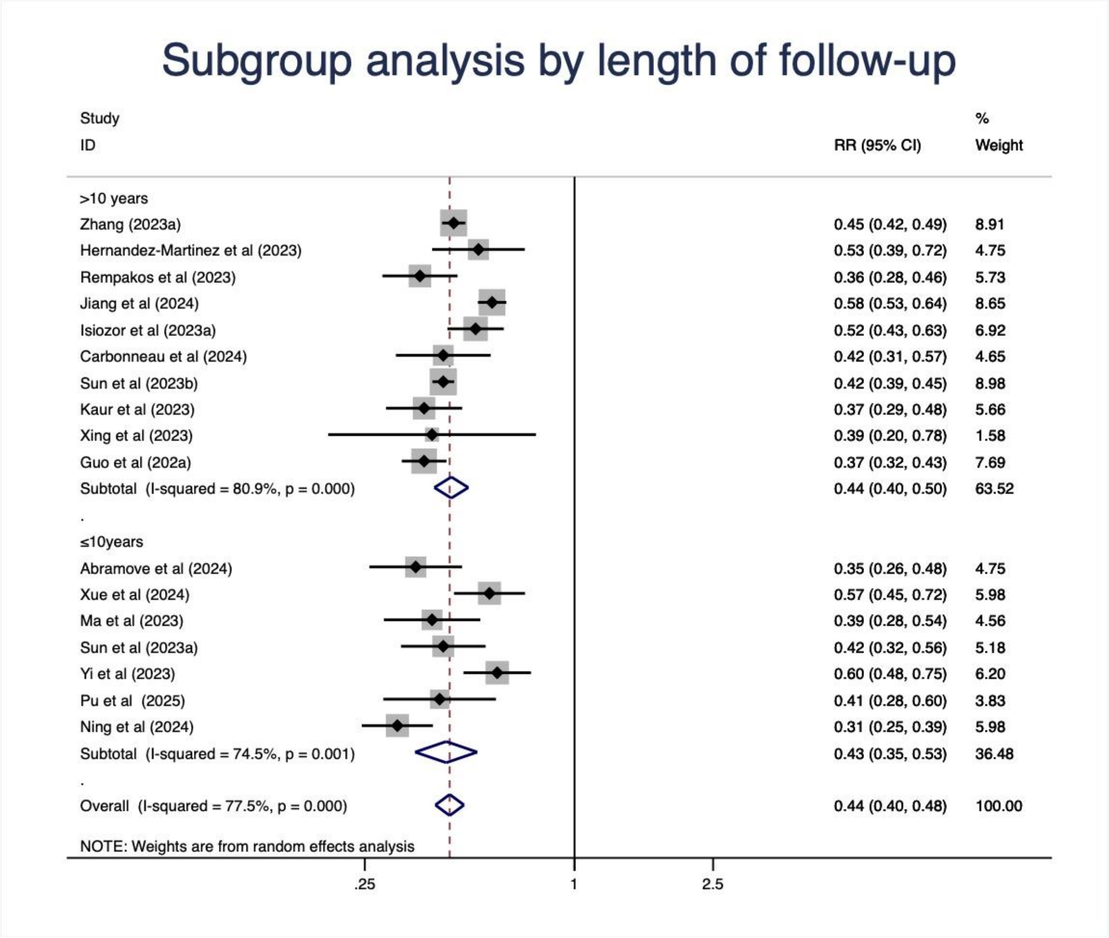

FigureS2. Forest plot of subgroup analysis by length of follow-up for the association between low and ideal CVH with all-cause mortality.

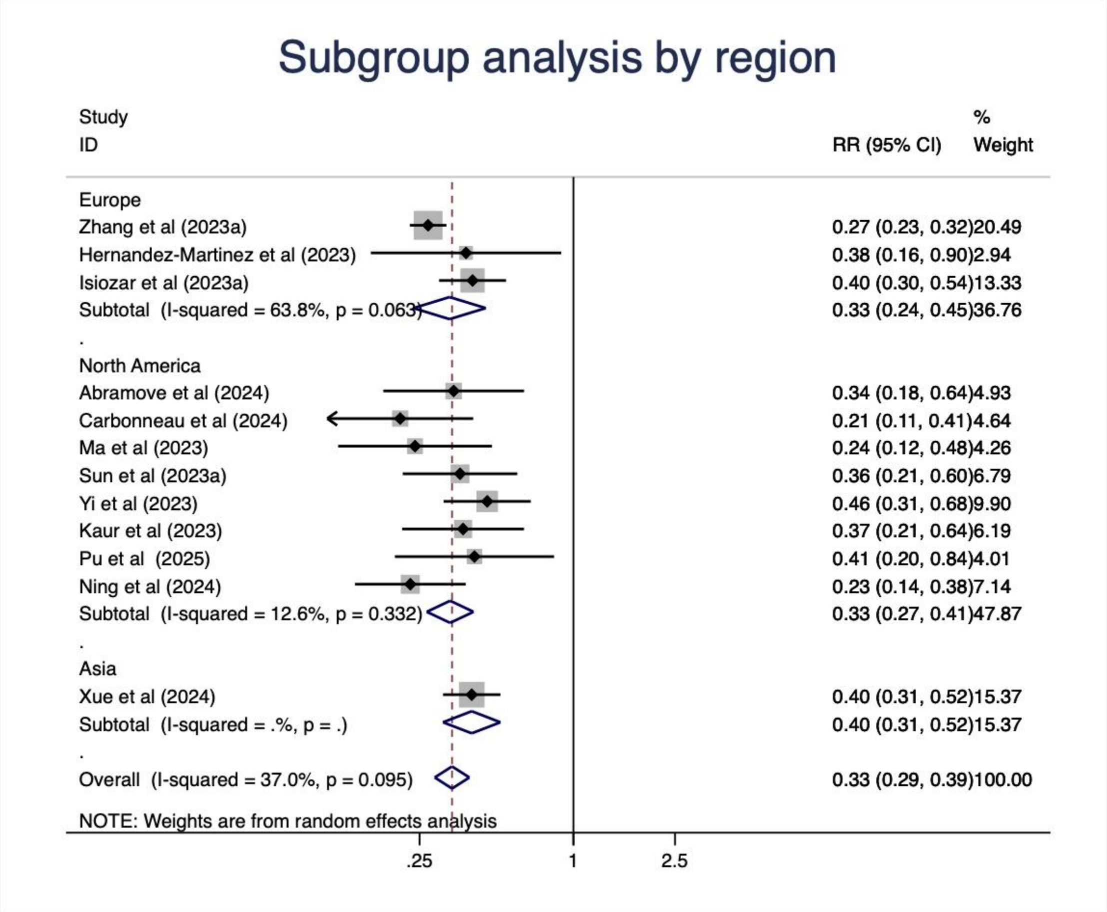

FigureS3. Forest plot of subgroup analysis by region for the association between low and ideal CVH with CVD mortality.

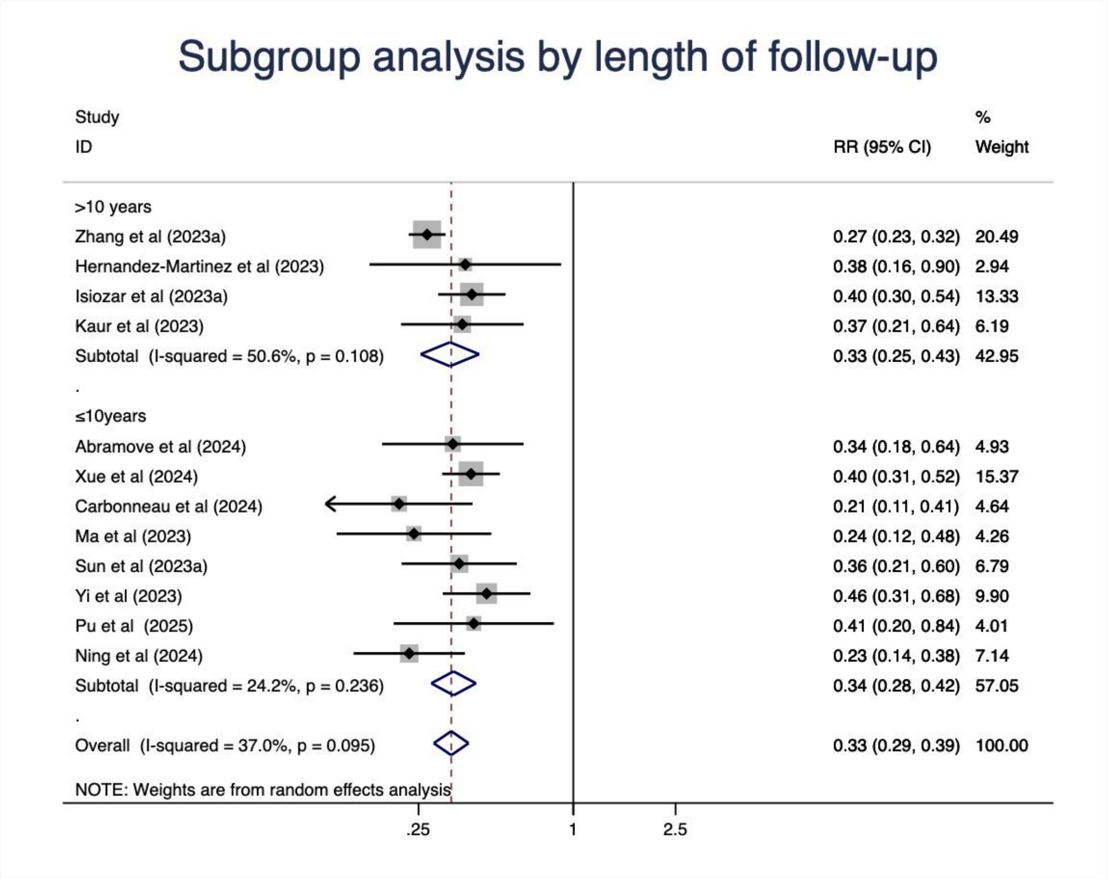

FigureS4. Forest plot of subgroup analysis by length of follow-up for the association between low and ideal CVH with CVD mortality.

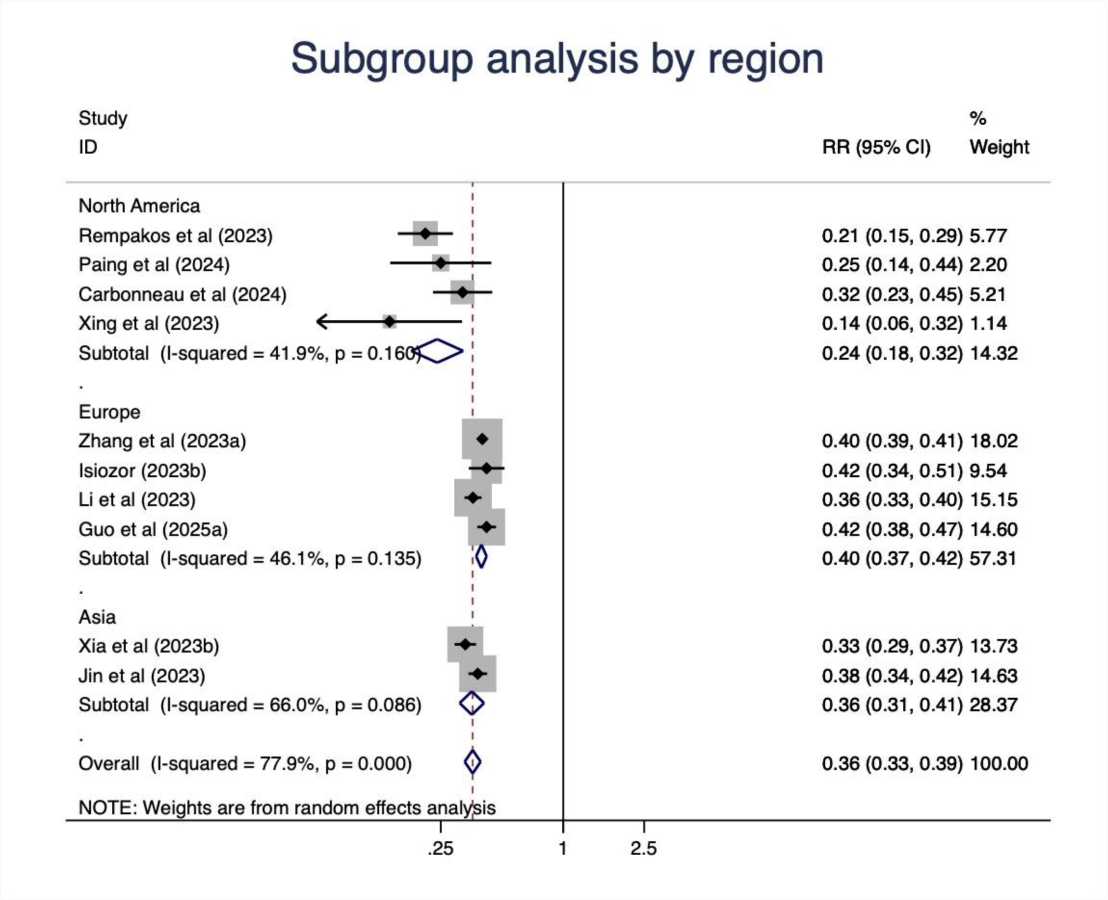

FigureS5. Forest plot of subgroup analysis by region for the association between low and ideal CVH with CVD incidence.

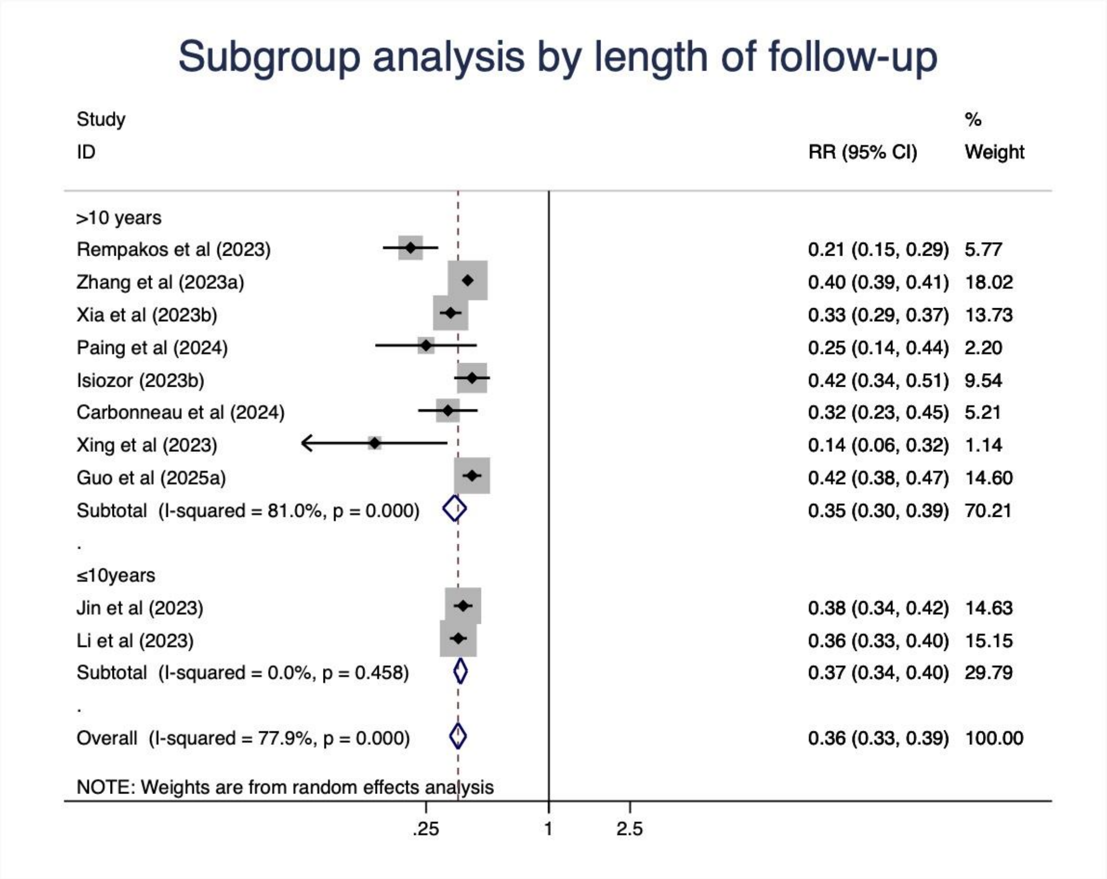

FigureS6. Forest plot of subgroup analysis by length of follow-up for the association between low and ideal CVH with CVD incidence.

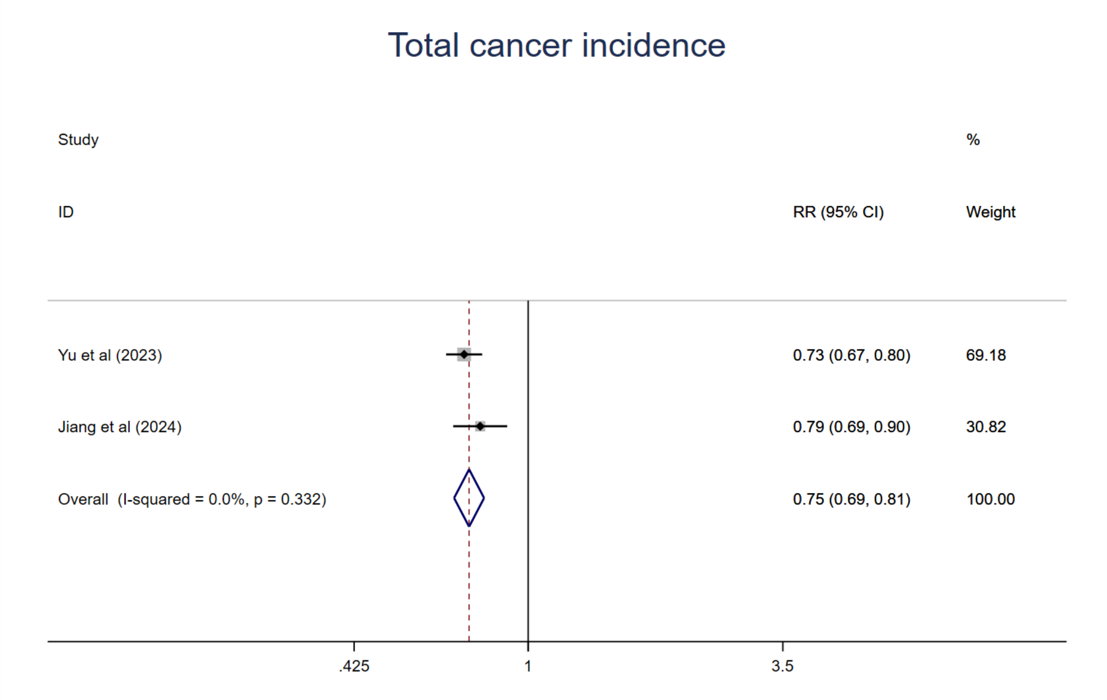

FigureS7. Two-group meta-analysis of the associations between low and ideal CVH

and total cancer.

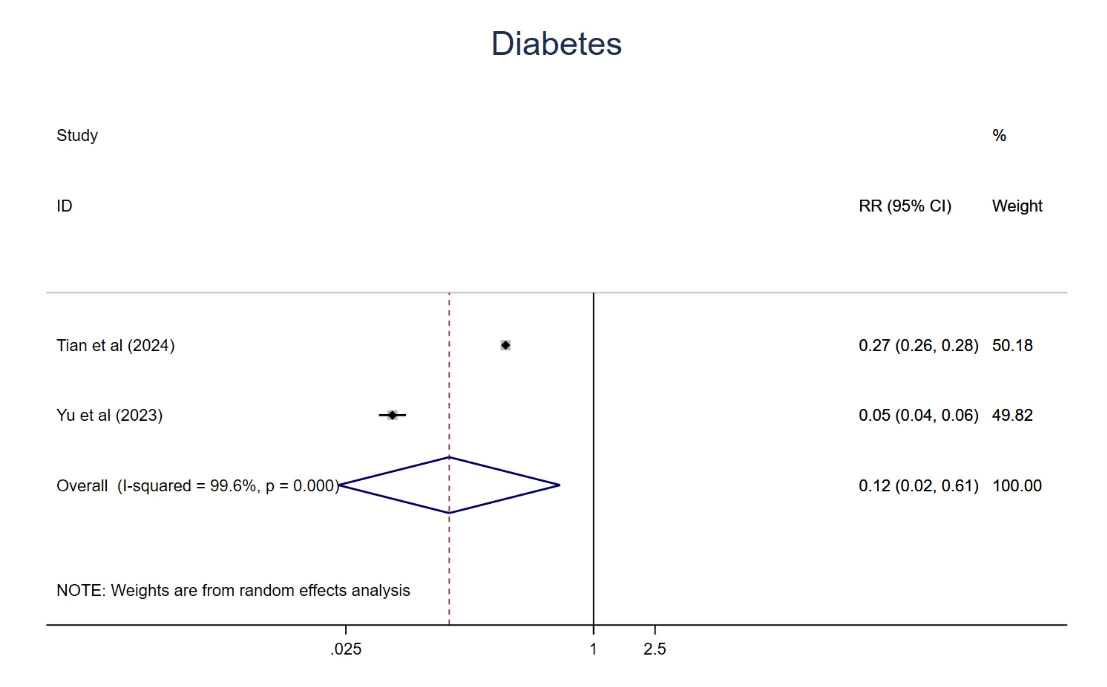

FigureS8. Two-group meta-analysis of the associations between low and ideal CVH and diabetes.

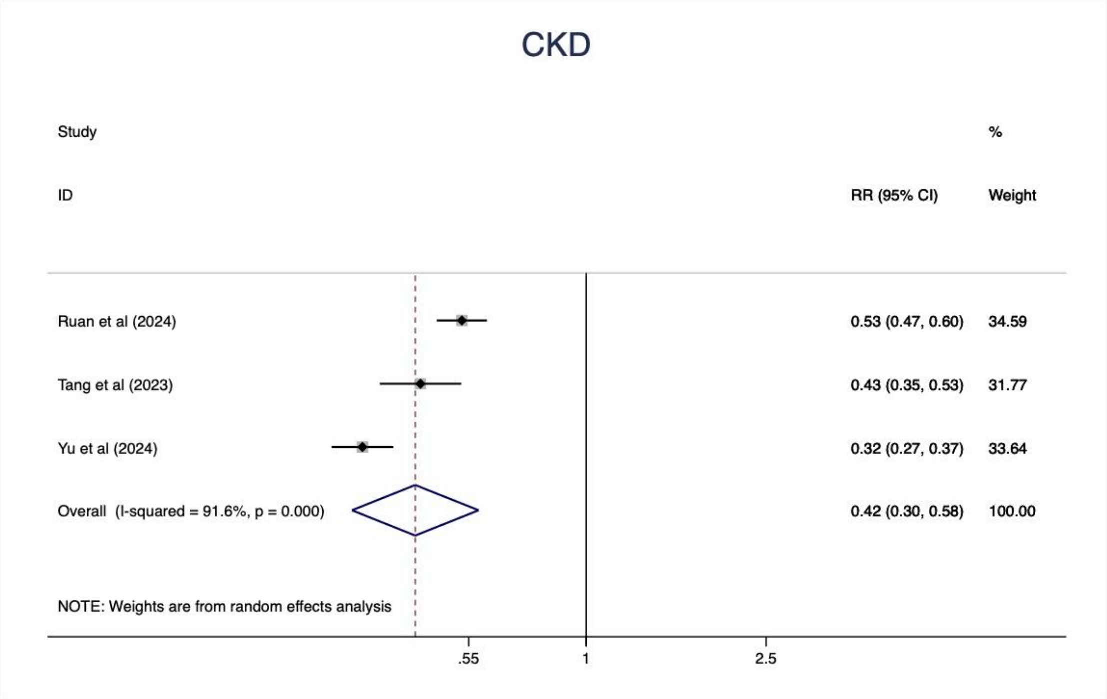

FigureS9. Two-group meta-analysis of the associations between low and ideal CVH with CKD incidence.

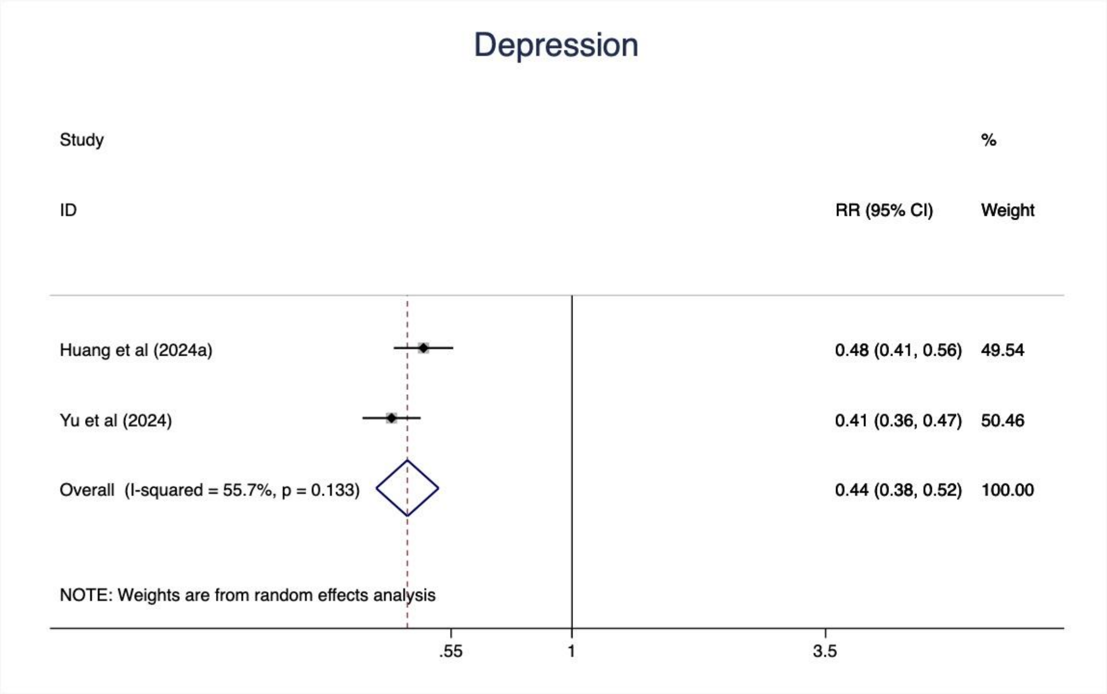

FigureS10. Two-group meta-analysis of the associations between low and ideal CVH with depression incidence.

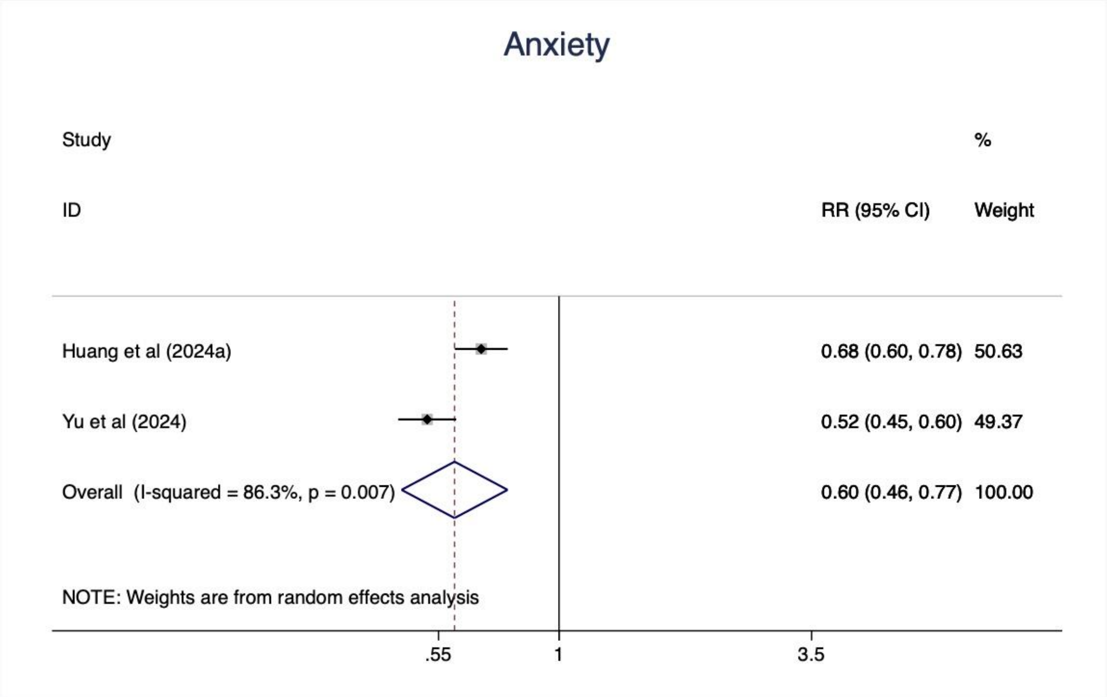

FigureS11. Two-group meta-analysis of the associations between low and ideal CVH with anxiety.

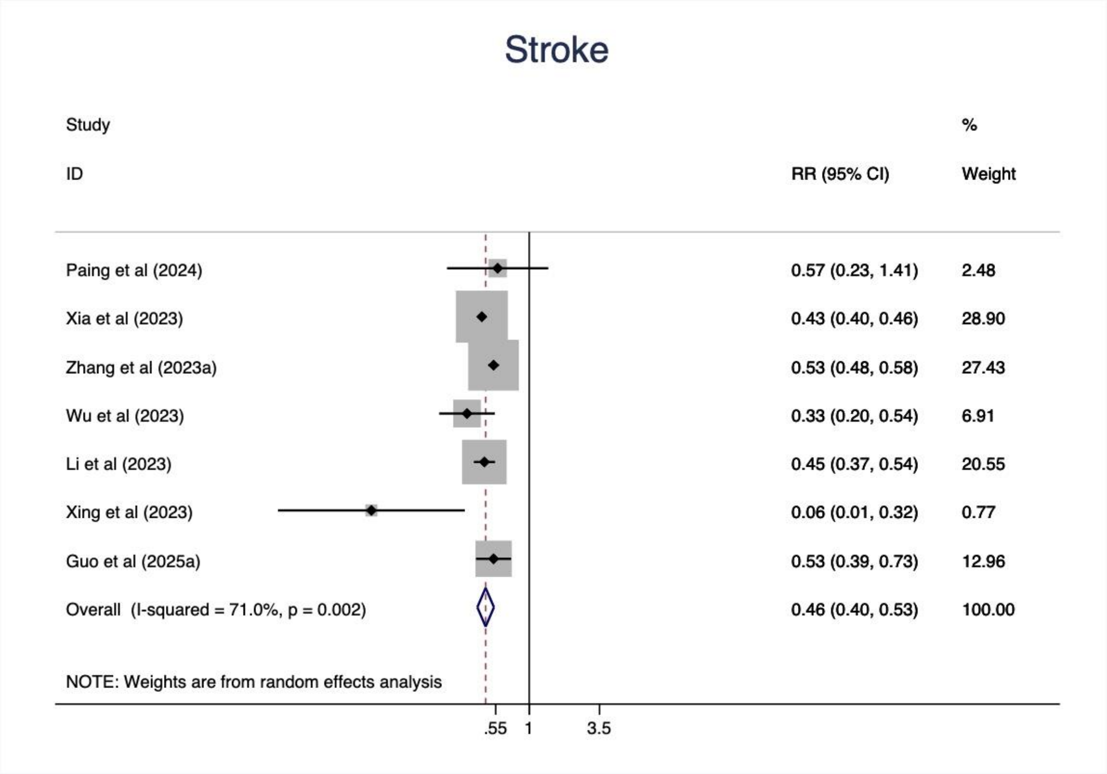

FigureS12. Two-group meta-analysis of the associations between low and ideal CVH with stroke incidence.

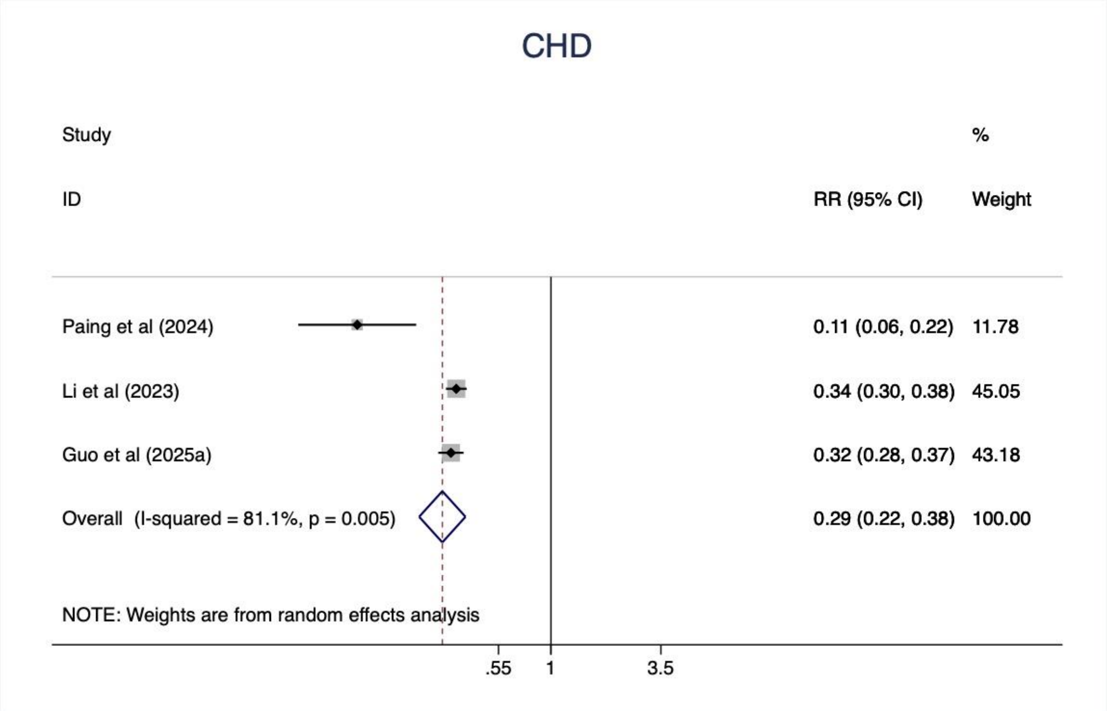

FigureS13. Two-group meta-analysis of the associations between low and ideal CVH with CHD incidence.

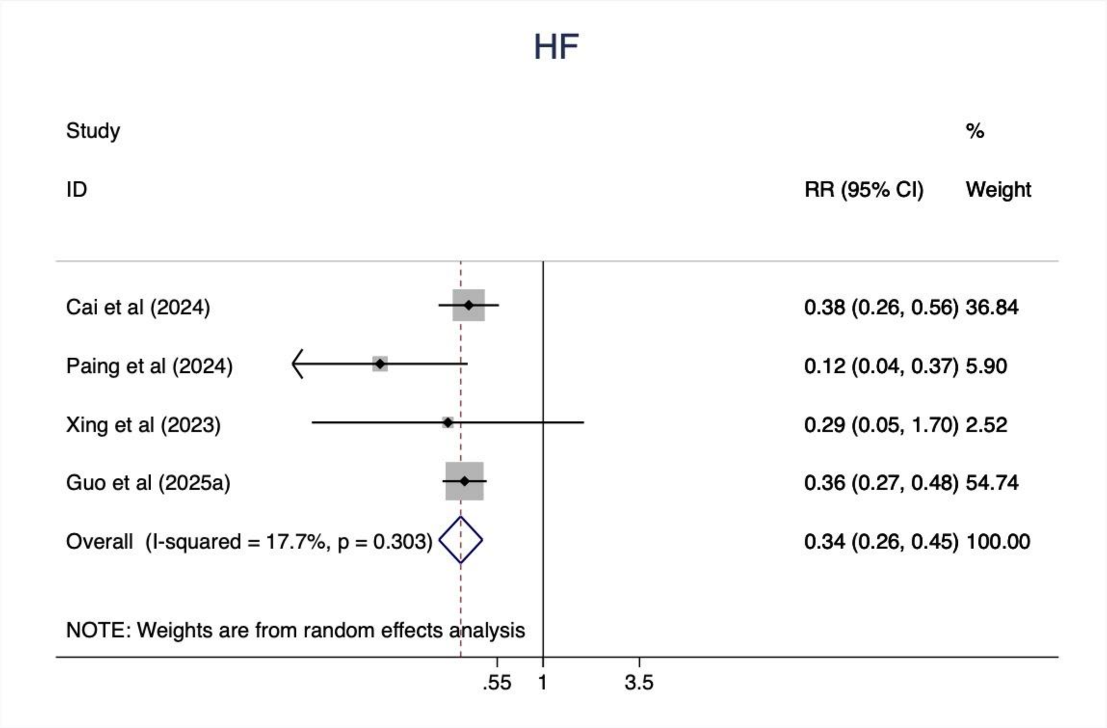

FigureS14. Two-group meta-analysis of the associations between low and ideal CVH with heart failure incidence.

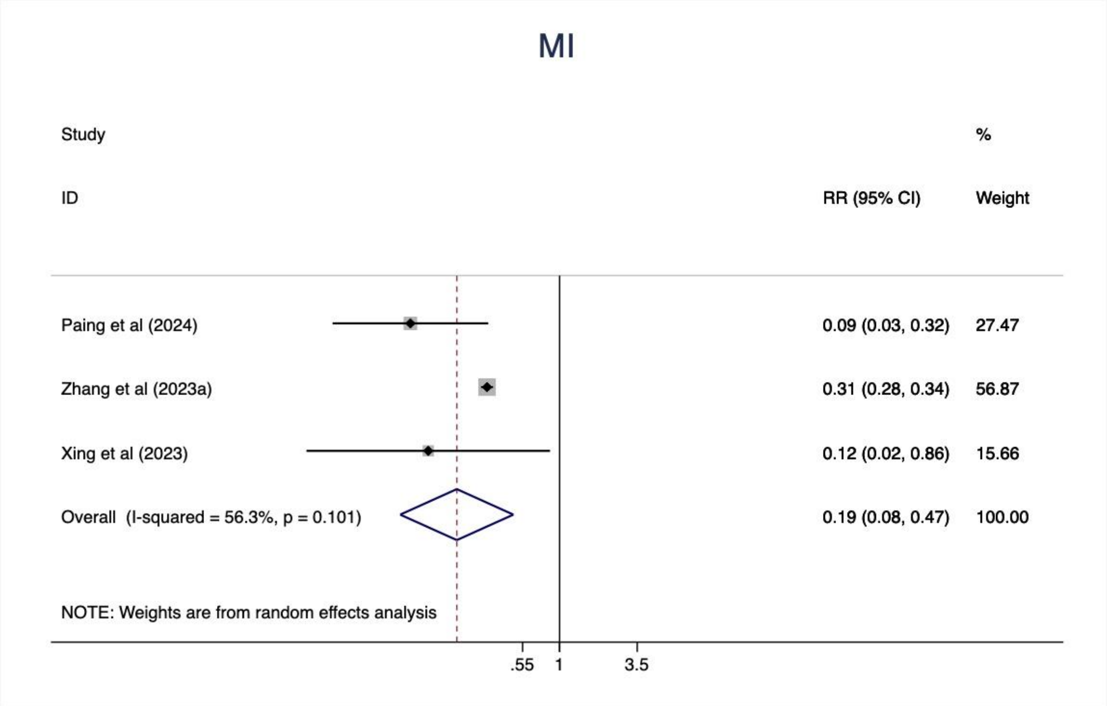

FigureS15. Two-group meta-analysis of the associations between low and ideal CVH with myocardial infarction incidence.

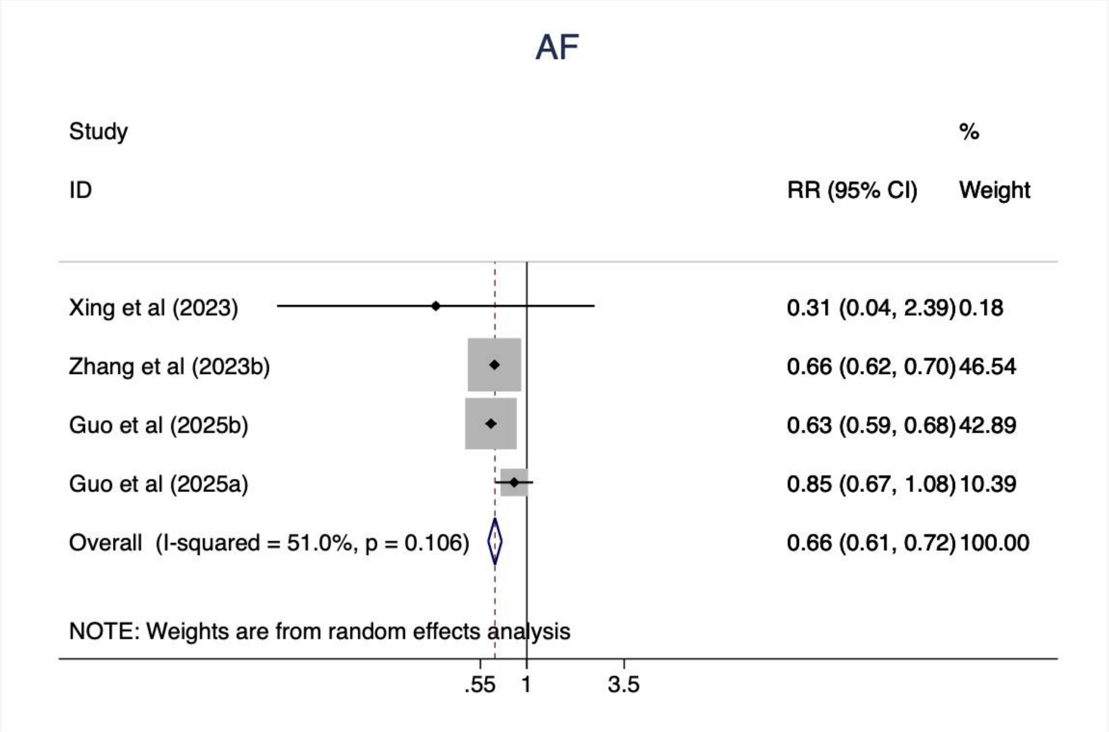

FigureS16. Two-group meta-analysis of the associations between low and ideal CVH with atrial fibrillation incidence.

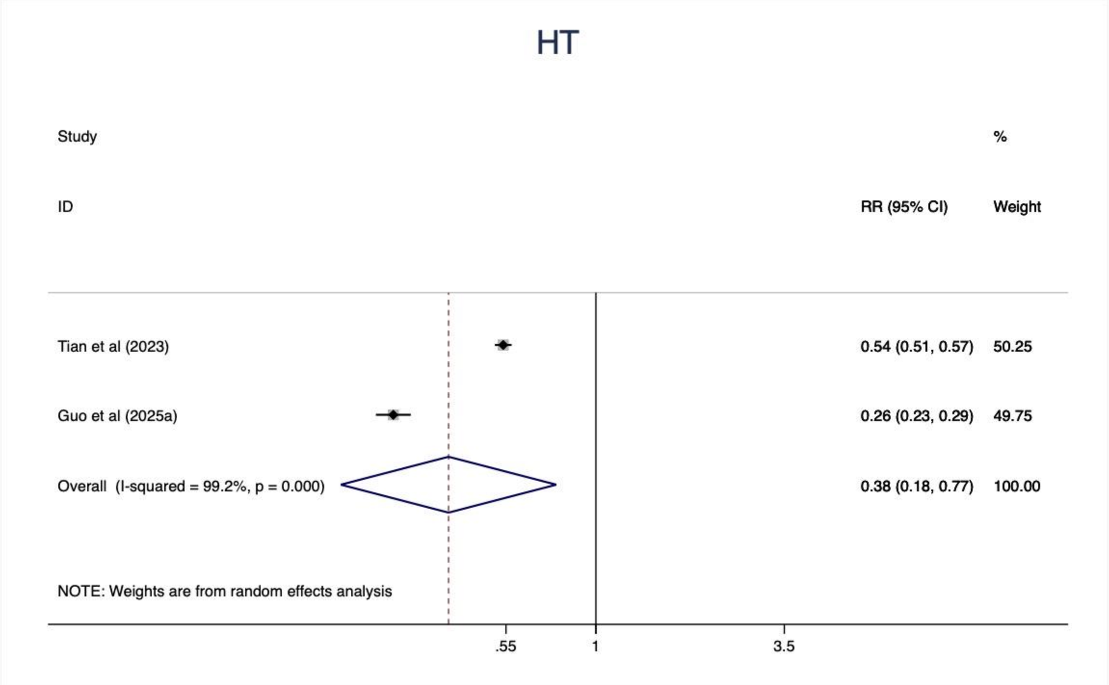

FigureS17. Two-group meta-analysis of the associations between low and ideal CVH with hypertension incidence.

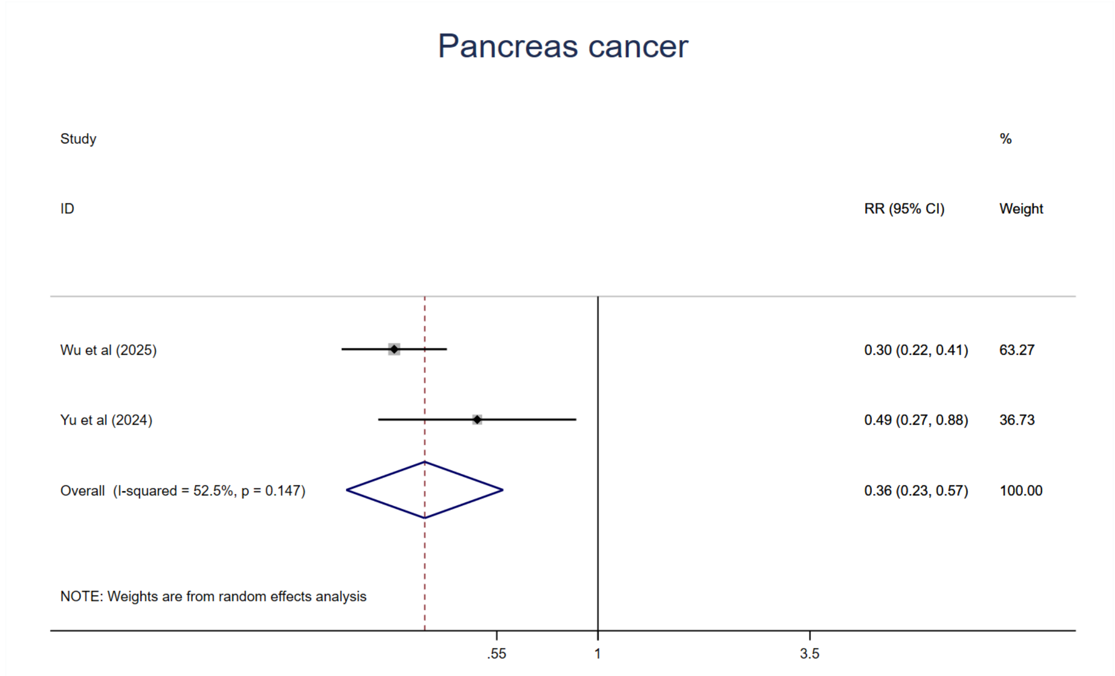

FigureS18. Two-group meta-analysis of the associations between low and ideal CVH with Pancreas cancer incidence.

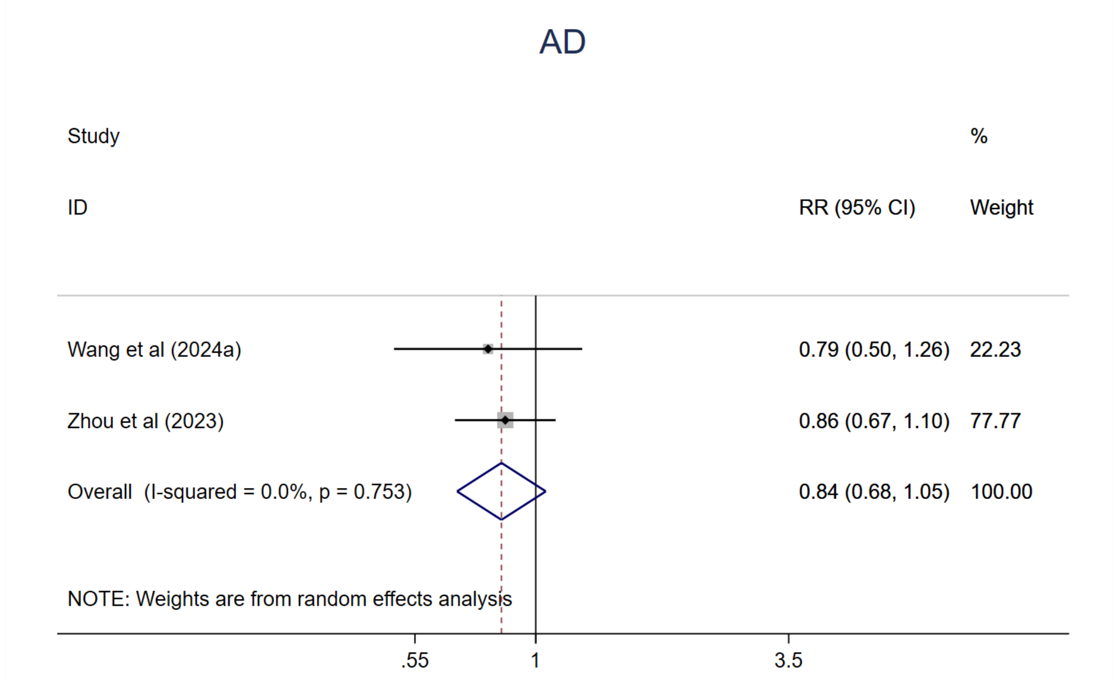

FigureS19. Two-group meta-analysis of the associations between low and ideal CVH with Alzheimer’s disease incidence.

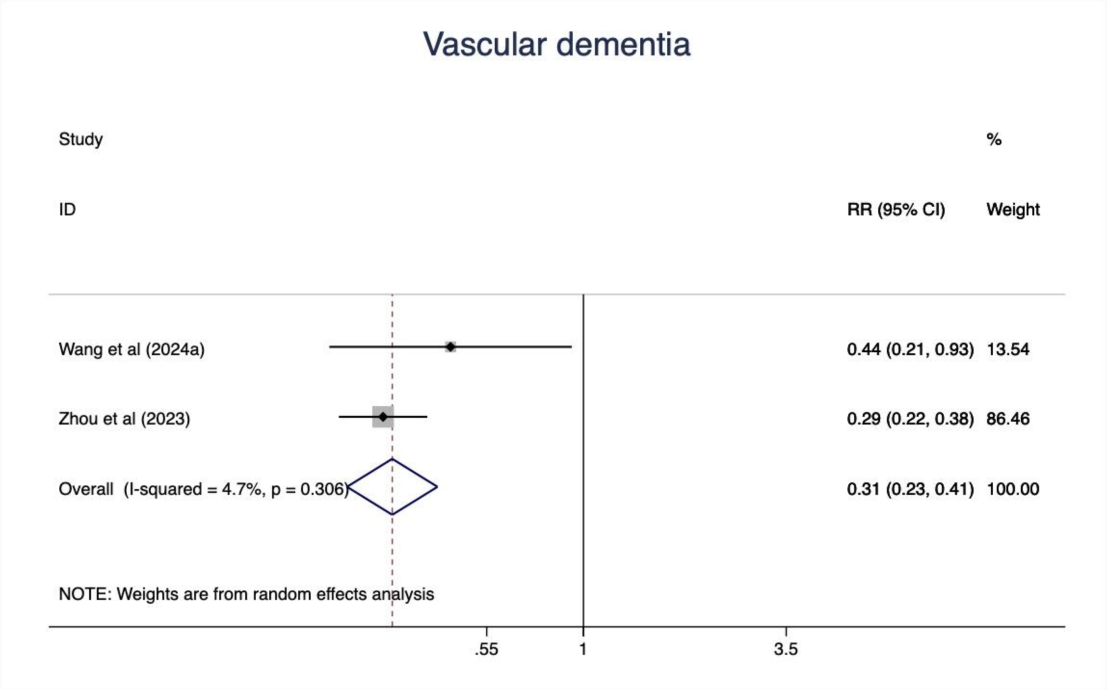

FigureS20. Two-group meta-analysis of the associations between low and ideal CVH with vascular dementia incidence.

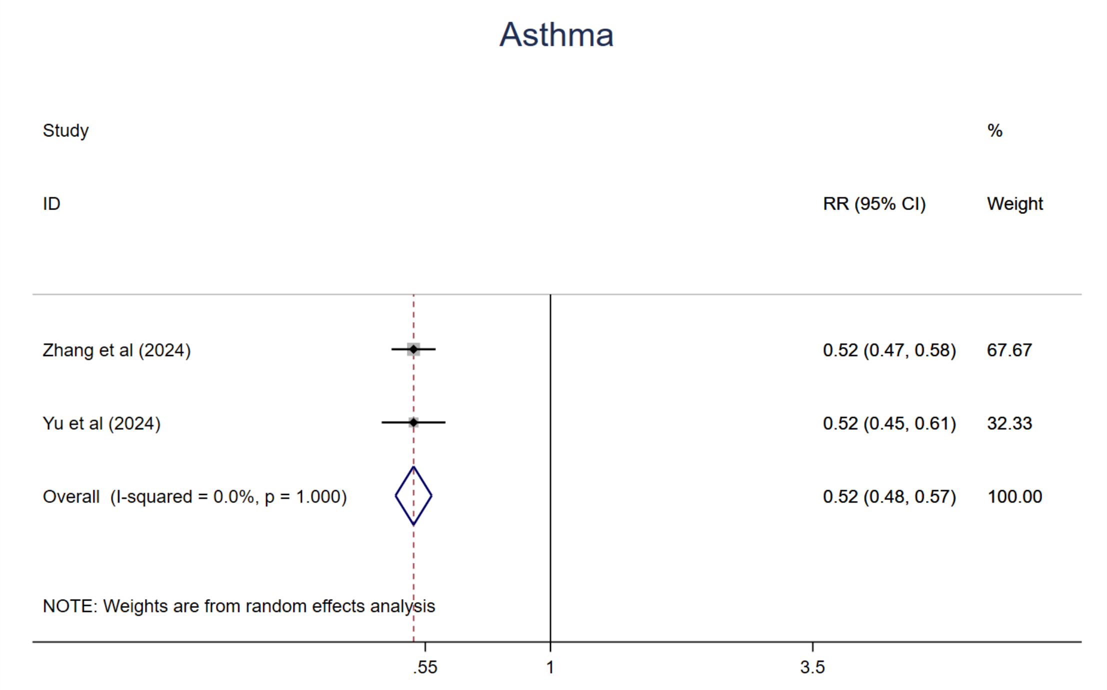

FigureS21. Two-group meta-analysis of the associations between low and ideal CVH with asthma incidence.

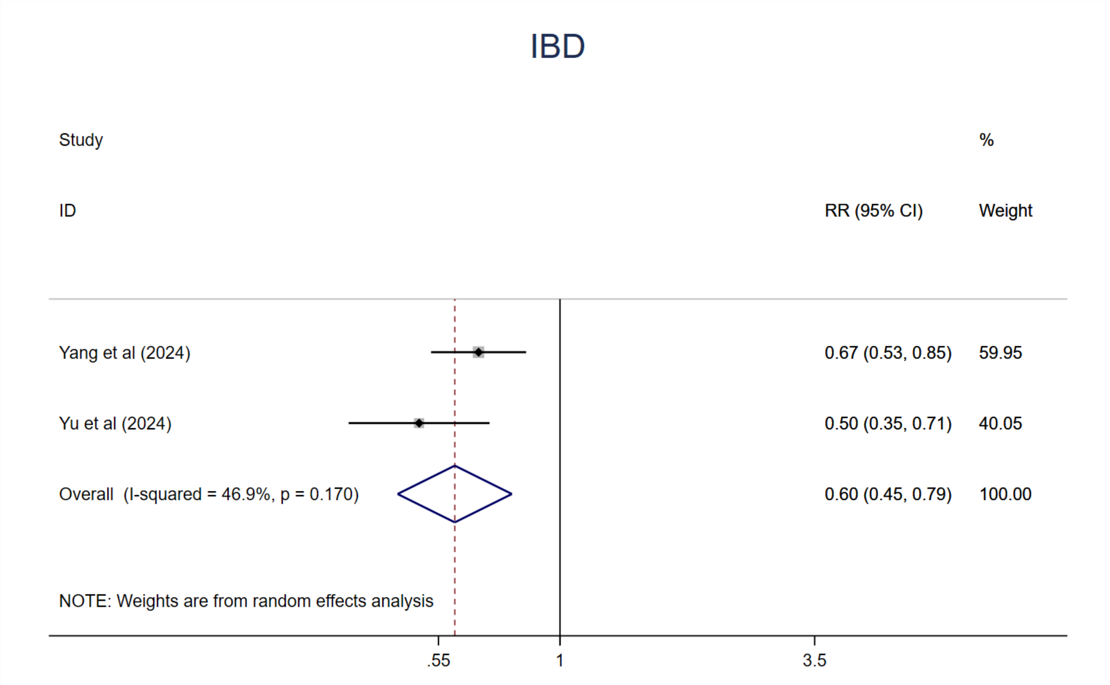

FigureS22. Two-group meta-analysis of the associations between low and ideal CVH with inflammatory bowel disease incidence.

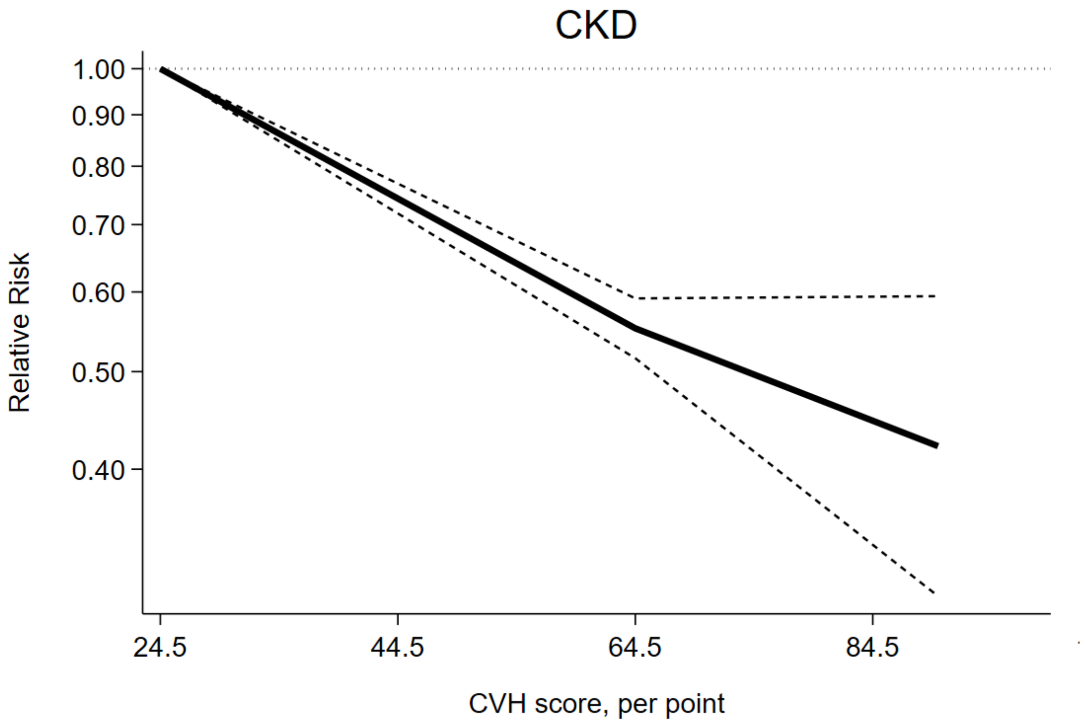

FigureS23. Non-linear dose-response meta-analysis of the association between CVH and CKD

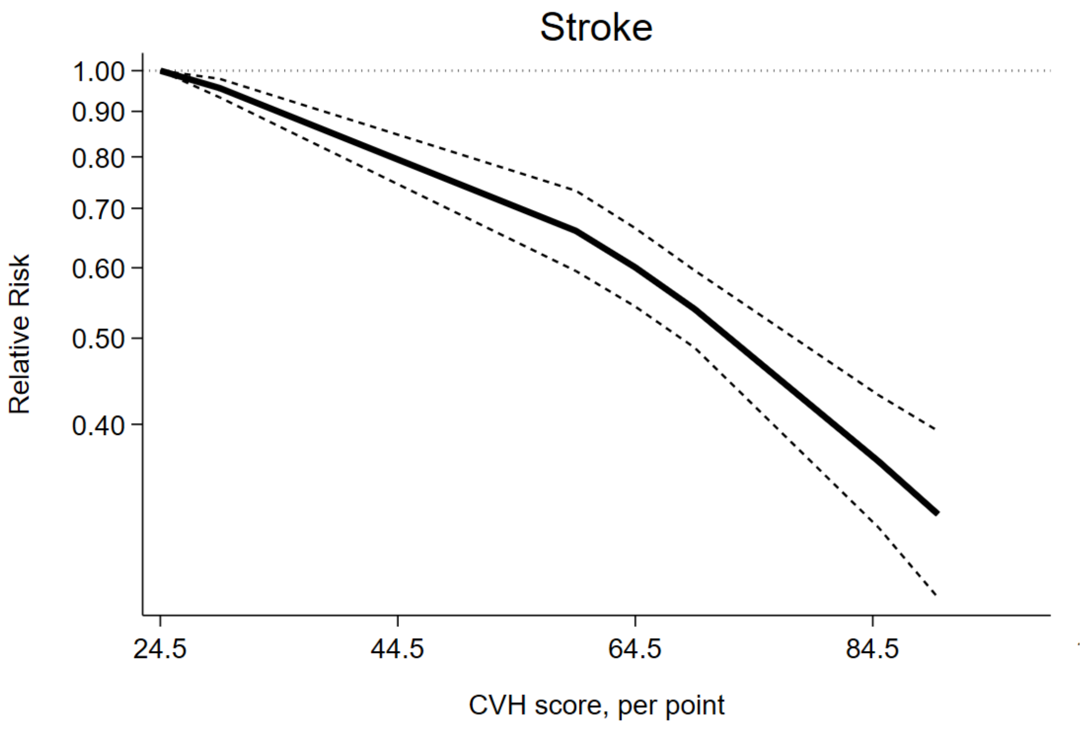

FigureS24. Non-linear dose-response meta-analysis of the association between CVH and stroke

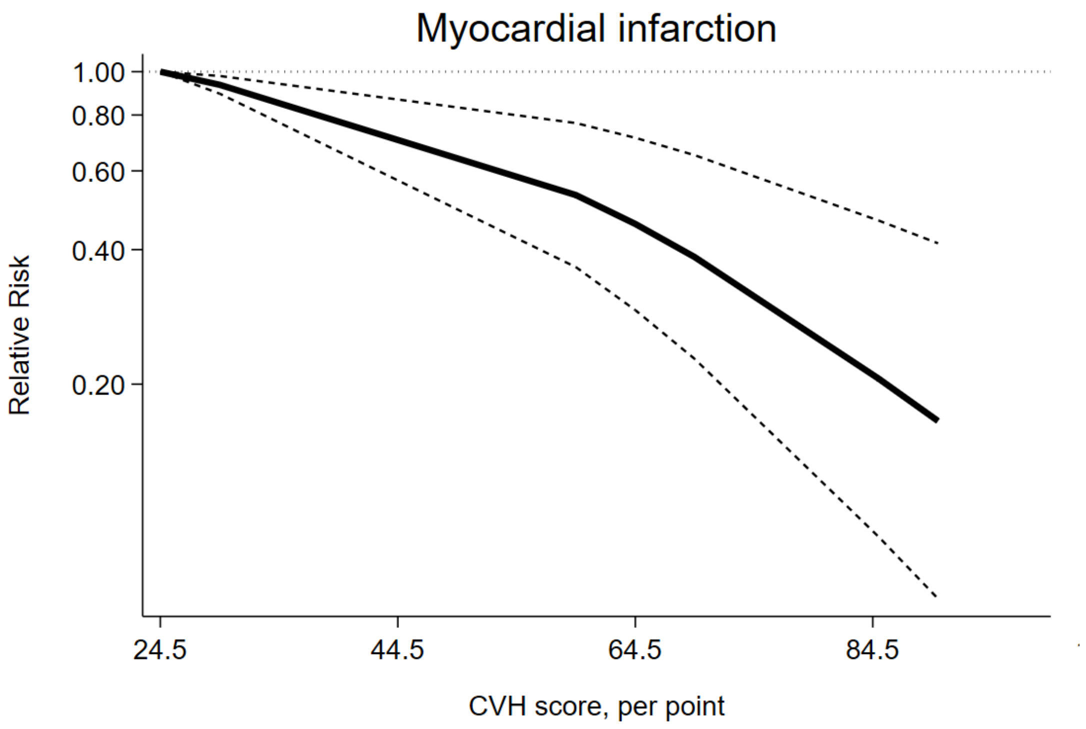

FigureS25. Non-linear dose-response meta-analysis of the association between CVH and myocardial infarction

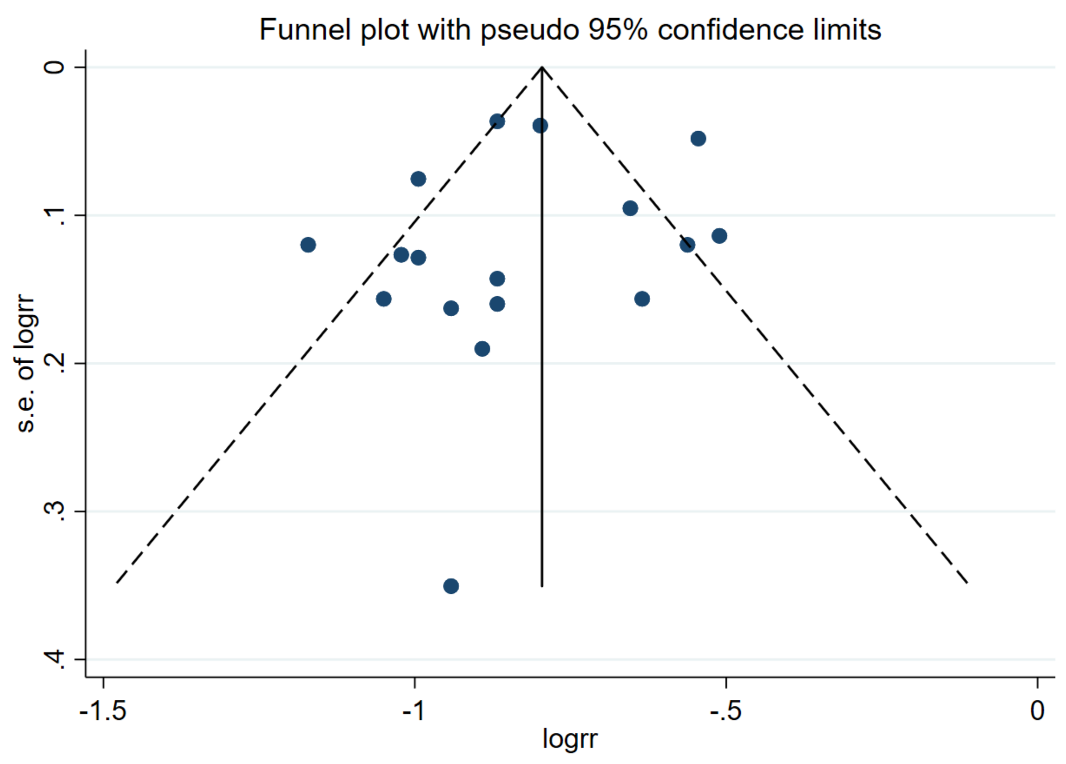

FigureS26. Funnel plot of the association between CVH and all-cause mortality

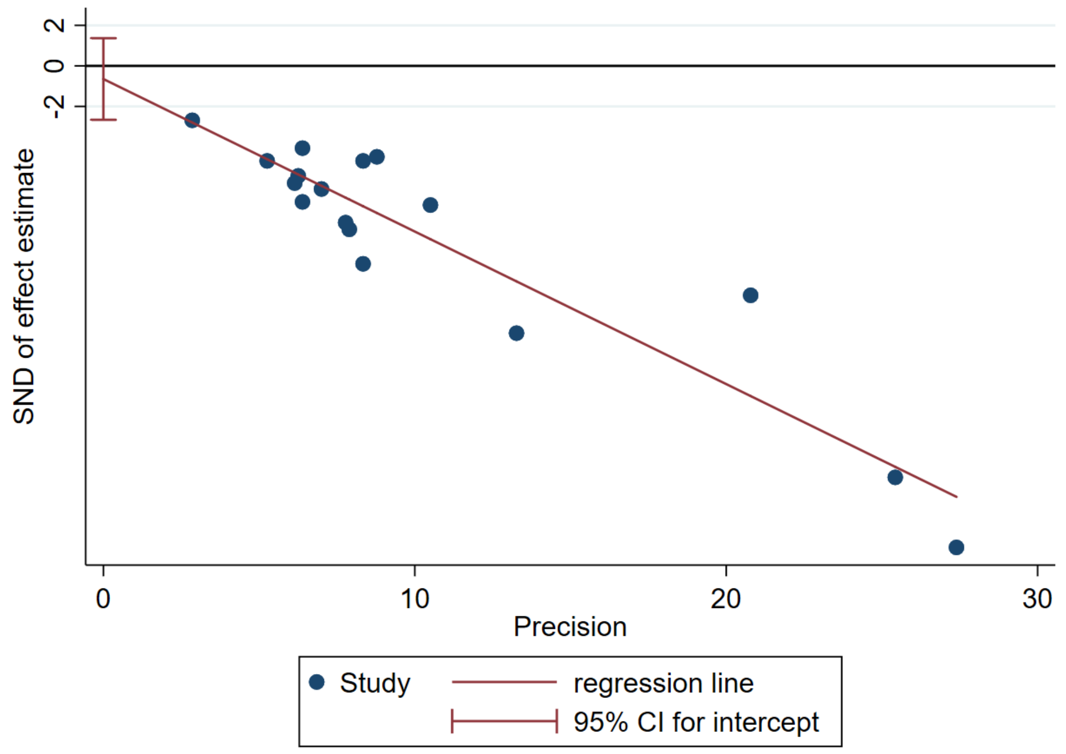

FigureS27. Publication bias analysis of the association between CVH and all-cause mortality

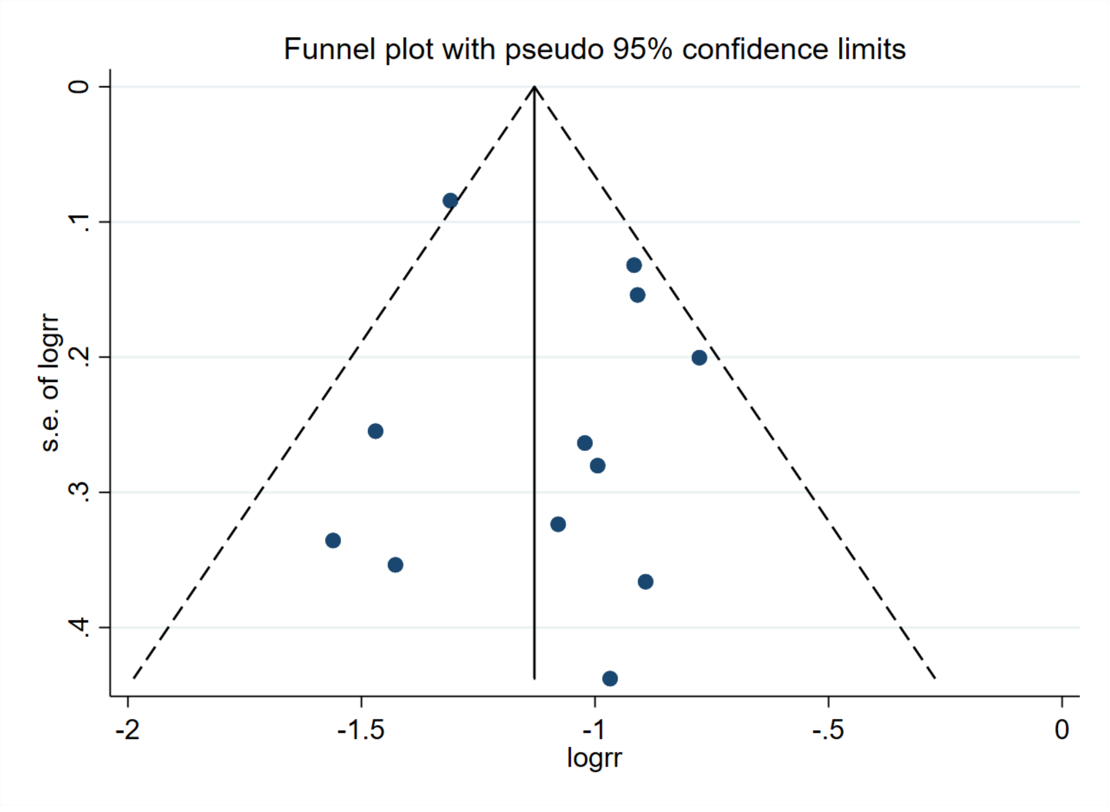

FigureS28. Funnel plot of the association between CVH and CVD mortality

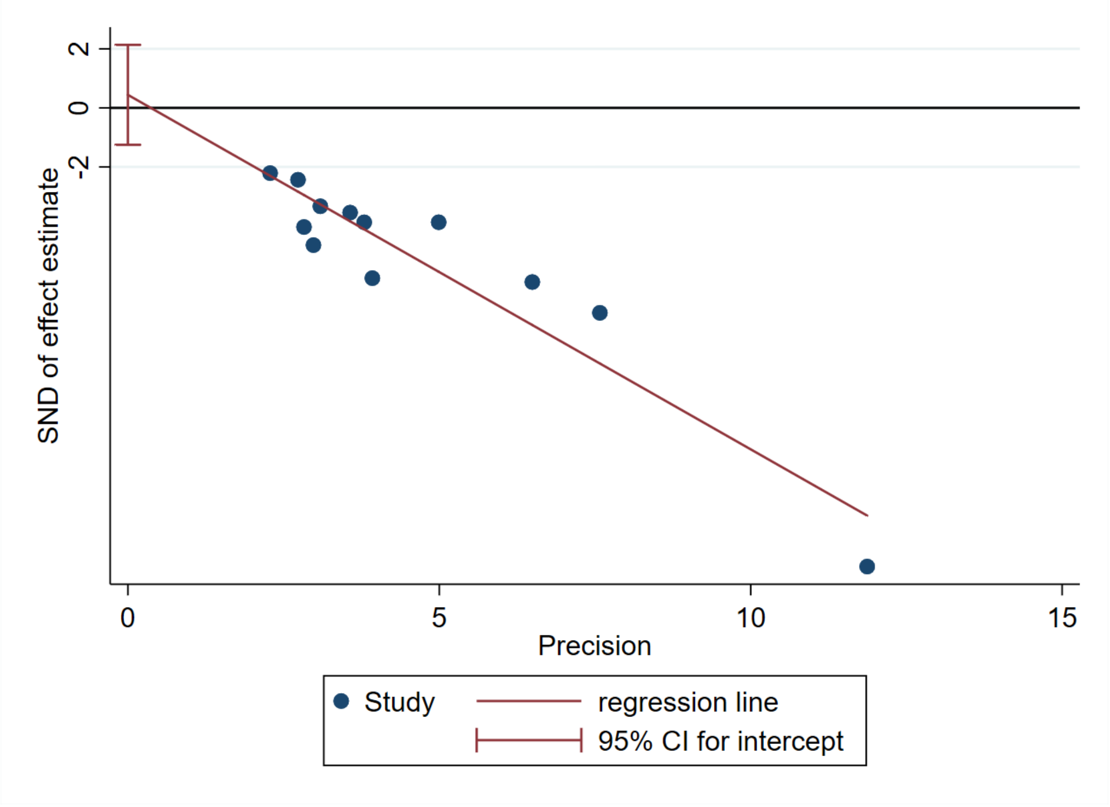

FigureS29. Publication bias analysis of the association between CVH CVD mortality

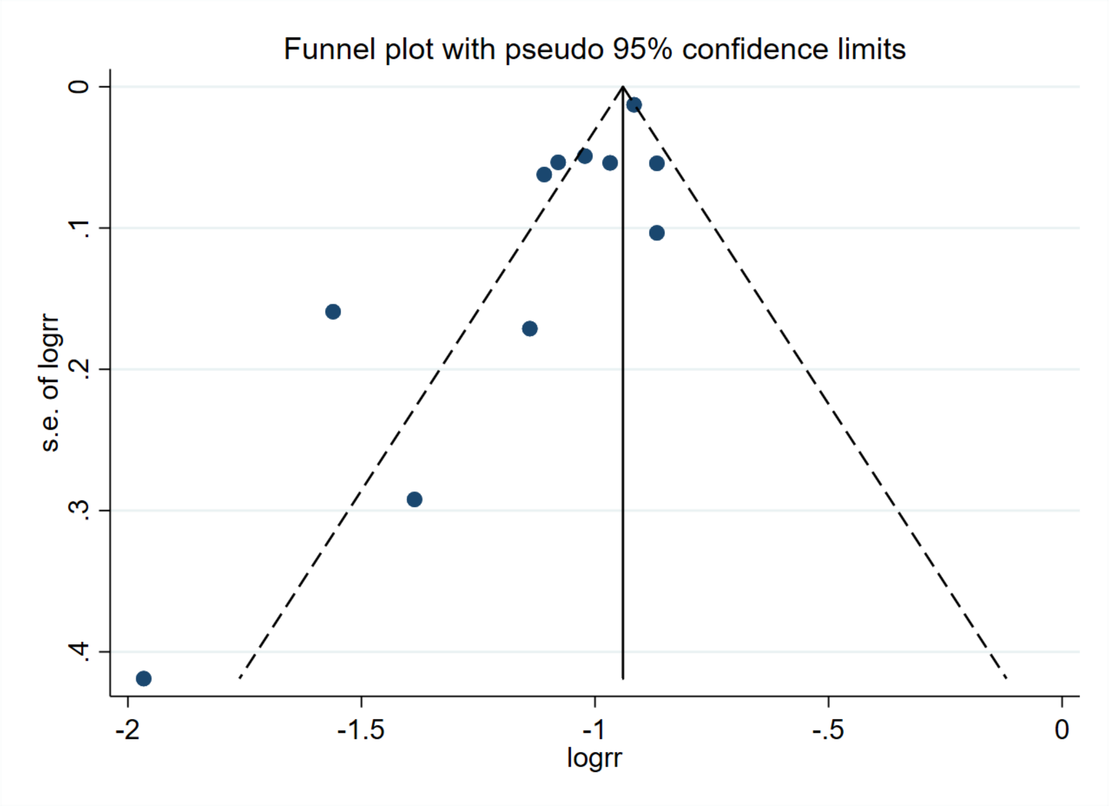

FigureS30. Funnel plot of the association between CVH and CVD incidence

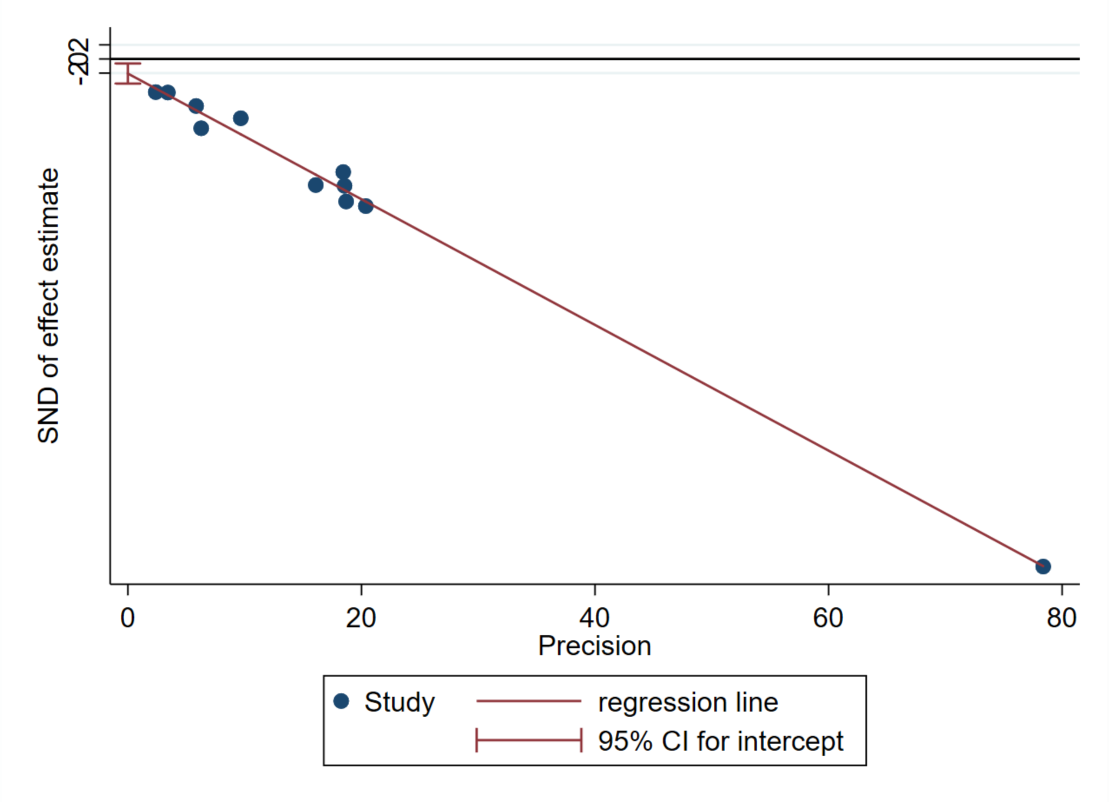

FigureS31. Publication bias analysis of the association between CVH CVD incidence

Refenrences

[1] Zhang J, Chen G, Habudele Z, et al. Relation of Life's Essential 8 to the genetic predisposition for cardiovascular outcomes and all-cause mortality: results from a national prospective cohort[J]. European Journal of Preventive Cardiology. 2023, 30(15): 1676-1685.

[2] Hernández-Martínez A, Duarte-Junior M A, Sotos-Prieto M, et al. Cardiovascular health in Spain based on the Life's Essential 8 and its association with all-cause and cardiovascular mortality: the ENRICA cohort[J]. Revista Espanola de Cardiologia. 2024, 77(5): 372-380.

[3] Abramov D, Kobo O, Mamas M A. Association of Cardiovascular Health Metrics and Mortality Among Individuals With and Without Cancer[J]. Journal of the American Heart Association. 2024, 13(5).

[4] Rempakos A, Prescott B, Xanthakis V, et al. Association of Life’s Essential 8 With Cardiovascular Disease and Mortality: The Framingham Heart Study[J]. Journal of the American Heart Association. 2023, 12(23).

[5] Jiang J, Ning N, Liu Y, et al. Association of Life's Essential 8 with all-cause mortality and risk of cancer: a prospective cohort study[J]. BMC Public Health. 2024, 24(1).

[6] Isiozor N M, Kunutsor S K, Voutilainen A, et al. Life’s Essential 8 and the risk of cardiovascular disease death and all-cause mortality in Finnish men[J]. European Journal of Preventive Cardiology. 2023, 30(8): 658-667.

[7] Xue T, Wang L, Zhang X, et al. Ambient fine particulate matter and Life's essential 8 and mortality in adults in China: A Nationwide retrospective cohort study[J]. Prev Med. 2024, 186: 108094.

[8] Carbonneau M, Li Y, Prescott B, et al. Epigenetic Age Mediates the Association of Life's Essential 8 With Cardiovascular Disease and Mortality[J]. J Am Heart Assoc. 2024, 13(11): e32743.

[9] Ma H, Wang X, Xue Q, et al. Cardiovascular Health and Life Expectancy Among Adults in the United States[J]. Circulation. 2023, 147(15): 1137-1146.

[10] Sun J, Li Y, Zhao M, et al. Association of the American Heart Association’s new “Life’s Essential 8” with all-cause and cardiovascular disease-specific mortality: prospective cohort study[J]. BMC Medicine. 2023, 21(1).

[11] Sun Y, Yu Y, Zhang K, et al. Association between Life's Essential 8 score and risk of premature mortality in people with and without type 2 diabetes: A prospective cohort study[J]. Diabetes/Metabolism Research and Reviews. 2023, 39(5).

[12] Yi J, Wang L, Guo X, et al. Association of Life's Essential 8 with all-cause and cardiovascular mortality among US adults: A prospective cohort study from the NHANES 2005－2014[J]. Nutrition, Metabolism and Cardiovascular Diseases. 2023, 33(6): 1134-1143.

[13] Kaur G, Kobo O, Parwani P, et al. Sex differences in Life's Essential Eight and its Association with mortality among US adults without known cardiovascular disease[J]. Am J Prev Cardiol. 2024, 18: 100685.

[14] Xing A, Tian X, Wang Y, et al. 'Life's Essential 8' cardiovascular health with premature cardiovascular disease and all-cause mortality in young adults: the Kailuan prospective cohort study[J]. European Journal of Preventive Cardiology. 2023, 30(7): 593-600.

[15] Guo D C, Chen Z T, Wang X, et al. Life's Essential 8, Genetic Susceptibility, and Incident Cardiac Arrhythmias: A Population-Based Prospective Cohort Study[J]. Canadian Journal of Cardiology. 2025, 41(1): 114-123.

[16] Pu B, Wang W, Lei L, et al. Association of depressive symptoms and cardiovascular health with mortality among U.S. adults[J]. Journal of Psychosomatic Research. 2025, 189.

[17] Ning N, Fan X, Zhang Y, et al. Joint association of cardiovascular health and frailty with all-cause

and cause-specific mortality: a prospective study[J]. Age and Ageing. 2024, 53(7).
[18] Lin L, Hu Y, Lei F, et al. Cardiovascular health and cancer mortality: evidence from US NHANES and UK Biobank cohort studies[J]. BMC Medicine. 2024, 22(1).
[19] Yu Y, Sun Y, Yu Y, et al. Life's Essential 8 and risk of non-communicable chronic diseases: Outcome-wide analyses[J]. Chinese Medical Journal. 2024, 137(13): 1553-1562.
[20] Xia X, Chen S, Tian X, et al. Association of Cardiovascular Health Assessed by the New Life’s Essential 8 Metrics With Years Lived Without Cardiovascular Disease[J]. Journal of the American Heart Association. 2023, 12(11).
[21] Paing P Y, Littman A J, Reese J A, et al. Association of Achievement of the American Heart Association’s Life’s Essential 8 Goals With Incident Cardiovascular Diseases in the SHFS[J]. Journal of the American Heart Association. 2024, 13(6).
[22] Jin C, Li J, Liu F, et al. Life's Essential 8 and 10-Year and Lifetime Risk of Atherosclerotic Cardiovascular Disease in China[J]. American Journal of Preventive Medicine. 2023, 64(6): 927-935.
[23] Isiozor N M, Laukkanen J A, Voutilainen A, et al. Life’s Essential 8 is associated with atherosclerotic cardiovascular disease but not venous thromboembolism in men: a prospective cohort study[J]. Annals of Medicine. 2023, 55(1).
[24] Li X, Ma H, Wang X, et al. Life's Essential 8, Genetic Susceptibility, and Incident Cardiovascular Disease: A Prospective Study[J]. Arteriosclerosis, Thrombosis, and Vascular Biology. 2023, 43(7): 1324-1333.
[25] Guo H, Wang S, Peng H, et al. Life's essential 8 and cardiovascular diseases progression among adults in the United Kingdom[J]. Metabolism: Clinical and Experimental. 2025, 162.
[26] Wu S, Wu Z, Yu D, et al. Life's Essential 8 and Risk of Stroke: A Prospective Community-Based Study[J]. Stroke. 2023, 54(9): 2369-2379.
[27] Cai A, Chen C, Wang J, et al. Life's Essential 8 and risk of incident heart failure in community population without cardiovascular disease: Results of the sub-cohort of China PEACE Million Persons Project[J]. Preventive Medicine. 2024, 178.
[28] Zhang J, Chen G, Xia H, et al. Associations of Life's Essential 8 and fine particulate matter pollution with the incidence of atrial fibrillation[J]. J Hazard Mater. 2023, 459: 132114.
[29] Tian X, Feng J, Chen S, et al. Baseline and longitudinal cardiovascular health using Life’s Essential 8 metrics with the risk of incident hypertension[J]. Clinical and Experimental Hypertension. 2023, 45(1).
[30] Tian X, Chen S, Xu Q, et al. Longitudinal cardiovascular health measured by life's essential 8 metrics with incident diabetes: A 13-year prospective cohort study[J]. Diabetes/Metabolism Research and Reviews. 2024, 40(3).
[31] He P, Zhang Y, Ye Z, et al. A healthy lifestyle, Life's Essential 8 scores and new-onset severe NAFLD: A prospective analysis in UK Biobank[J]. Metabolism: Clinical and Experimental. 2023, 146.
[32] Huang J, Xin Z, Cao Q, et al. Association between updated cardiovascular health construct and risks of non-alcoholic fatty liver disease[J]. Nutrition, Metabolism and Cardiovascular Diseases. 2024, 34(2): 317-325.
[33] Yaqin W, Shuwen D, Ting Y, et al. Cumulative exposure to AHA Life's Essential 8 is associated with nonalcoholic fatty liver disease: a large cohort study[J]. Nutrition and Metabolism. 2024, 21(1).
[34] Ruan Y X, Wu M X, Gao J W, et al. AHA Life’s Essential 8 and new-onset CKD: a prospective cohort study from the UK Biobank[J]. Clinical and Experimental Nephrology. 2024, 28(4): 325-336.
[35] Tang R, Wang X, Li X, et al. Adherence to Life's Essential 8 and incident chronic kidney disease: a prospective study of 147,988 UK Biobank participants[J]. American Journal of Clinical Nutrition. 2023,

118(4): 804-811.
[36] Wang Q, Yu R, Dong C, et al. Association and prediction of Life's Essential 8 score, genetic susceptibility with MCI, dementia, and MRI indices: A prospective cohort study[J]. Journal of Affective Disorders. 2024, 360: 394-402.
[37] Zhou R, Chen H W, Li F R, et al. “Life's Essential 8” Cardiovascular Health and Dementia Risk, Cognition, and Neuroimaging Markers of Brain Health[J]. Journal of the American Medical Directors Association. 2023, 24(11): 1791-1797.
[38] Lu Z, Ke J, Yang H, et al. Cardiovascular health and risks of atrial fibrillation and its prognosis[J]. American Journal of Preventive Cardiology. 2025, 21.
[39] Huang X, Zhang J, Liang J, et al. Association of Cardiovascular Health With Risk of Incident Depression and Anxiety[J]. American Journal of Geriatric Psychiatry. 2024, 32(5): 539-549.
[40] Zhang H, Chang Q, Yang H, et al. Life's Essential 8, genetic predisposition, and risk of incident adult-onset asthma: a prospective cohort study[J]. American Journal of Clinical Nutrition. 2024, 119(1): 100-107.
[41] Yang H, Chang Q, Ji C, et al. Life's essential 8, genetic susceptibility, and risk of inflammatory bowel diseases: a population-based cohort study[J]. INTERNATIONAL JOURNAL OF BEHAVIORAL NUTRITION AND PHYSICAL ACTIVITY. 2024, 21(1).
[42] Wu Z, Zeng L, Fang Z, et al. Life's Essential 8, genetic susceptibility, and risk of incident pancreatic cancer: A prospective cohort study[J]. International Journal of Cancer. 2025, 156(3): 566-574.
[43] Liang B, Zha L, Peng B, et al. Association of Cardiovascular Health with the Incidence of Venous Thromboembolism: A Prospective Study of 275,149 Participants from the UK Biobank[J]. Thrombosis and Haemostasis. 2024.
[44] Zhao Y, Song Y, Li X, et al. Association of Life’s Essential 8 cardiovascular health with breast cancer incidence and mortality according to genetic susceptibility of breast cancer: a prospective cohort study[J]. Breast Cancer Research. 2024, 26(1).
[45] Gao J, Liu Y, Ning N, et al. Better Life’s Essential 8 Is Associated With Lower Risk of Diabetic Kidney Disease: A Community-Based Study[J]. Journal of the American Heart Association. 2023, 12(17).
[46] Hou X, Zhang N, Chen S, et al. Association of life's essential 8 and risk of fragility fractures: A large cohort study[J]. Journal of Orthopaedic Research. 2024, 42(4): 798-805.
[47] Huang J, Xu X, Zhou Y, et al. Age-specific difference in the temporal relationships between updated cardiovascular health construct and arterial stiffness in middle-aged and older adults[J]. GEROSCIENCE. 2024, 46(2): 2051-2062.
[48] Lei Y, Zhang L, Shan Z, et al. Poor healthy lifestyle and life's essential 8 are associated with higher risk of new-onset migraine: a prospective cohort study[J]. JOURNAL OF HEADACHE AND PAIN. 2024, 25(1).
[49] Huang X, Liang J, Zhang J, et al. Association of life’s essential 8 with chronic cardiovascular-kidney disorder: a prospective cohort study[J]. BMC Public Health. 2024, 24(1).
[50] Isiozor N M, Kunutsor S K, Voutilainen A, et al. Cardiovascular health metrics and risk of heart failure in a Finnish population: a prospective cohort study[J]. ESC Heart Failure. 2023, 10(2): 1222-1230.
